# Supplementary material for: Impact of metabolic syndrome and its components on bone remodeling in adolescents
Source: PLoS One. 2021 Jul 1;16(7):e0253892. doi: 10.1371/journal.pone.0253892 (PMC8248706; doi:10.1371/journal.pone.0253892)
Supplement: Attachment — Submitted filename: PONE-D-20-24612_R1_response.pdf [file pone.0253892.s003.pdf]

PLOS ONE

Impact of metabolic syndrome and its components on bone remodeling in adolescents

--Manuscript Draft--

**Manuscript Number:**	PONE-D-20-24612R1
**Article Type:**	Research Article
**Full Title:**	Impact of metabolic syndrome and its components on bone remodeling in adolescents
**Short Title:**	Metabolic syndrome and bone remodeling
**Corresponding Author:**	Tamara Beres Lederer Goldberg, M.D., Ph.D.<br>Universidade Estadual Paulista Julio de Mesquita Filho<br>Botucatu, São Paulo BRAZIL
**Keywords:**	adolescents; Bone; metabolic syndrome
**Abstract:**	Abstract<br>Osteoporosis and metabolic syndrome (MetS) are diseases that have serious public health consequences, reducing the quality of life of patients and increasing morbidity and mortality, with substantial healthcare expenditures.<br>Objective<br>To evaluate the impact of MetS on bone mineral density (BMD) and biochemical markers of bone formation and resorption in adolescents with excess weight.<br>Method<br>A descriptive and analytical cross-sectional study that evaluated 271 adolescents of both sexes (10 to 16 years). From the total sample, 42 adolescents with excess weight and the presence of MetS (14%) were selected. A further 42 adolescents with excess weight and without MetS were selected and matched for chronological age, bone age, and pubertal developmental criteria to those with MetS, for each sex. The adolescents were submitted to anthropometry, blood pressure measurement, and biochemical tests, as well as evaluation of BMD and the bone biomarkers osteocalcin (OC), bone alkaline phosphatase (BAP), and carboxy-terminal telopeptide (S-CTx).<br>Results<br>The adolescents with excess weight and MetS exhibited a significant decrease in BMD and concentrations of BAP, OC, and S-CTx compared to the matched group, except for OC in boys. A negative and significant correlation was observed between total body BMD and BAP (r = -0.55568; p= 0.005), OC (r = -0.81760; p= <.000), and S-CTx (r = -0.53838; p= 0.011) in girls.<br>Conclusion<br>The presence of MetS is detrimental to the bone mineral density (BMD) and biochemical markers of bone formation and resorption in adolescents with excess weight .
**Order of Authors:**	Valéria Nóbrega da Silva
	Tamara Beres Lederer Goldberg, M.D., Ph.D.
	Carla Cristiane Silva
	Cilmery Suemi Kurokawa
	Luciana Nunes Mosca Fiorelli
	Anapaula da Conceição Bisi Rizzo
	José Eduardo Corrente
**Response to Reviewers:**	To Benjamin Udoka Nwosu, MD<br>Academic Editor<br>PLOS ONE<br><br>Dear Dr. Udoka Nwosu<br>First, we are grateful that you considered the topic of this manuscript to be an interesting area of exploration in pediatric bone metabolism and for sending your personal comments and the excellent comments from the reviewers regarding our article "Impact of metabolic syndrome and its components on bone remodeling in

subjected?

too strong of the word for conclusion

Powered by Editorial Manager® and ProduXion Manager® from Aries Systems Corporation

adolescents". We have carefully read and reread the comments to ensure that we have effectively responded to the suggestions and questions raised. Below are the comments made by the reviewers and, in sequence, our answers, highlighted in blue. The passages added to the original article are also highlighted in blue.
We would like to take this opportunity to thank you for the possibility of re-presenting the study to PLOS One with the incorporation of the comments made by the reviewers.

Comments:
1.The manuscript is quite voluminous and some of the sections such as the Introduction and Discussion should be edited and shortened considerably for clarity.
Answer: We have reduced the two sections as suggested.

2.Authors should attempt to offer an explanation why all the bone turnover markers were decreased in the cohort with MetS.
Answer: An explanation has been added to the discussion.

3.The authors should also endeavor to explain the reason for the negative correlation of all bone markers (both bone formation and bone resorption markers) with BMD.
Answer: Dear Editor, in previous studies from our group with eutrophic adolescents, we observed negative correlations between bone markers and bone mineral density in females. We have written:
“Bone remodeling biomarkers (BAP, OC, and S-CTx) presented a significant negative correlation with CA, BA, and pubertal stage. This finding demonstrated that the more mature the participants, the higher their BMD values. Through analyses of biomarkers, that study demonstrates the changes in bone remodeling occurring in the second decade of life, revealing high marker concentrations in the early adolescence years and significantly reduced concentrations in late adolescence. These analyses correlate with the BMD values, which represent bone mass incorporation, and indicate an inversely proportional behavior showing the highest BMD values associated with the lowest concentrations of formation and resorption of biomarkers (Fortes et al., 2014).”
Thus, this negative correlation represents the expected evolution in eutrophic adolescents and, possibly, in those with MetS. However, we highlight that the concentrations of the markers were even lower in the group with MetS and there were stronger correlations between biomarkers and bone mineral density, as shown in the manuscript. Although the increase in BMD was less than expected (Nóbrega da Silva et al., 2014), a negative correlation was found only in girls with bone markers. Similar results were found in a previous study (Yilmaz et al., 2005) which reported: “A negative correlation of BMD at two sites with bone formation markers was significant in girls ($p < 0.001$), but no correlation could be detected in boys ($p > 0.05$)”.
References:
Fortes CM, Goldberg TB, Kurokawa CS, Silva CC, Moretto MR, Biason TP, Teixeira AS, Nunes HR. Relationship between chronological and bone ages and pubertal stage of breasts with bone biomarkers and bone mineral density in adolescents. J Pediatr (Rio J). 2014;90(6):624-31.

Nóbrega da Silva V, Goldberg TBL, Mosca LN, Rizzo ACB, Teixeira AS, Corrente JE. Metabolic syndrome reduces bone mineral density in overweight adolescents. Bone. 2014; 66:1-7.

Yilmaz, D., Ersoy, B., Bilgin, E. et al. Bone mineral density in girls and boys at different pubertal stages: relation with gonadal steroids, bone formation markers, and growth parameters. J Bone Miner Metab.2005; 23:476–482.

4.The finding of reduced bone formation markers and BMD in MetS subjects suggests that the underlying mechanism or the reduced biomarkers and BMD is most likely due to increased systemic inflammation which was not explored in this study. To establish this, however, will require a study of the patients’ pro-inflammatory and anti-inflammatory cytokines which was not done in this study. Therefore, this should be discussed in the section on limitations of the study.
Answer: You are correct. We have removed the text that introduced this possibility about the inflammatory state and presented it as one of the limitations of the present study.
5.It was not clear why the authors did not evaluate vitamin D status in their subjects which could have shed some light on their findings. This is a significant limitation of the

study and should be discussed in the section on limitations.
Answer: We agree that this is another limiting factor in the study because 25-hydroxyvitamin D [25 (OH) D] was not dosed. However, as all the adolescents included and evaluated were overweight and the groups were paired, we believe that both groups had Vitamin D insufficiency/deficiency, whether or not they had MetS.

6.Table 1 should be completely redone. Table 1should present the comparison between Mets patients and non-Mets patients for anthropometry and biochemical parameters.
Answer: Dear Editor, Table 1 corresponded to the IDF criteria for the classification of Metabolic Syndrome and has been removed, and the description of the components of Metabolic Syndrome has been added to the manuscript.
The current table 1 is entitled : Comparison of adolescents with and without metabolic syndrome according to sex, bone age, tanner stage, anthropometric measures, and body mass index, and BMD and transformed BMD of both sexes have been added .
The bone markers are included in table 2, entitled: Comparison of metabolic syndrome components and bone biomarkers between adolescents with and without MetS according to sex.

7.Please review the comments from the reviewers below and provide the anthropometric and radiological data in z-scores where appropriate.
Answer: Dear Editor, each Reviewer's comment has been carefully analyzed and we have provided detailed answers below.

Review Comments to the Author

1.Reviewer #1: Comment 1: The study had a decent cohort size of adolescents matched by age, bone age, and puberty. In addition, it was stratified by sex. The authors compared adolescents with MetS with adolescents without MetS as controls. It would also be interesting how these findings relate to normal weight adolescents. Though evaluation of obesity and MetS and BMD in pediatrics has been published, the authors took this further to attempt to look at affects of MetS in adolescents with excess weight on bone remodeling markers, which is a very interesting and important concepts.
Answer: Dear Reviewer, we appreciate your encouraging comments and your careful analysis of our study. We have tried to answer each question in order to improve the manuscript.
2. The conclusion, objective in introduction worded strongly. BMD and bone mass used interchangeably in the conclusion. BMD data and analyses not prov[illegible] groups.
Answer: You are absolutely right, we included a table without the re[illegible] evaluated in the various analyzed sites. We have changed table 1 a[illegible] BMD means, as well as the transformed BMDs.

too strong of a conclusion. the study had correlations but no causations.

The conclusion has been changed to: The presence of MetS is detrimental to the bone mineral density (BMD) and biochemical markers of bone formation and resorption in adolescents with excess weight.

3. BMD data was provided in tables showing r correlations with bone resorption markers only. Can the authors show BMD data and analysis done to draw the main discussion and conclusion points?
Answer: We have added the means and SD of the BMD to Table 1. Tables 3 and 4 show BMD and Biomarker values (OC, BAP, and S-CTx) for boys and girls respectively.

4.Was DXA measurements adjusted for height? please explain why or why not.
Answer: Densitometry was not adjusted for height, but for weight. This is the same technique that we used in a manuscript that we previously published in BONE (Nóbrega da Silva et al., 2014) citing the study of Hill et al. (2011). As the groups were matched for CA, BA, and pubertal development, they did not differ in height, as can be seen in Table 1. Were the groups matched by height?

References:
Nóbrega da Silva V, Goldberg TB, Mosca LN, Bisi Rizzo Ada C, Teixeira Ados S, Corrente JE. Metabolic syndrome reduces bone mineral density in overweight

Powered by Editorial Manager® and ProduXion Manager® from Aries Systems Corporation

adolescents. Bone. 2014;66:1-7.

Hill KM, Braun MM, Egan KA, Martin BR, McCabe LD, Peacock M, et al. Obesity augments calcium-induced increases in skeletal calcium retention in adolescents. J Clin Endocrinol Metab 2011 ;96:2171–7.

5. Authors report BMI, but no BMI %iles or SDS. In pediatric data report the latter to categorize the cohort.
Answer: You are absolutely right about the percentile assessment. This analysis was performed for each of the individuals included in the study, as we explain in the methods. However, due to the number of words, we did not include it in the text or in a table, as we understood that we would have a lot of data and that this was not the focus of the study, since we only included overweight individuals. We present the nutritional classification according to percentiles of the groups paired by CA, BA with MeTs - and MeTS + only for information, remembering that adolescents with a BMI between the 85th and 95th percentile were classified as overweight, between the 95th and 99th percentile as obese (Kuczmarski, Ogden, Guo, 2002), and above the 99th percentile as extremely obese (Barlow, 2007).

Nutritional evaluation of adolescents matched and included in the study sample.
Female (n=48)Male (n=36)
MetS (-)MetS (+)MetS (-)MetS (+)
Overweight11 (45.9%)4 (16.7%)8 (44.4%)3 (16.7%)
Obese11 (45.9%)14 (58.3%)8 (44.4%)5 (27.8%)
Severe Obese2 (8.3%)6 (25.0%)2 (11.1%)10 (55.6%)

References:
Kuczmarski RJ, Ogden CL, Guo SS, Grummer-Strawn LM, Flegal KM, Mei Z, Wei R, Curtin LR, Roche AF, Jhonson CL 2000. CDC Growth Charts for the United States: Methods and Development. Vital Health Stat. 2002; 11 246: 1-190.

Barlow SE, Expert Committee. Expert committee recommendations regarding the prevention, assessment, and treatment of child and adolescent overweight and obesity: summary report. Pediatrics. 2007; 120: Suppl 4:S164-92.

6. In the lines 296-298, the authors state that the study showed a significant reduction in BMD at different sites. It is not clear what the data were. The authors should clarify and show BMD data to support the statement.
Answer: We apologize and have changed Table 1 to present the data.

7. Lines 312-319: The authors have an interesting finding pertaining to the sex differences, they speculate that it may be attributable to the differences in biochemical and anthropometric characteristics. It is not clear what biochemical characteristics are being referred to. Similarly, line 322-323, states males were much more compromised metabolically, however, it is not clear in which sense and what metabolic derangements this assessment is being referred to.
Answer: Dear Reviewer, we are referring to each of the nutritional and biochemical criteria used for the diagnosis of MeTS, in this case among the biochemicals HDLc, triglycerides, and glycemia. Among girls without MeTS, we were able to match with a group in which few biochemical/metabolic alterations were detected in 42% of them.

In the group without the presence of Metabolic Syndrome, 42% of the patients presented no biochemical alterations, and 45.9% demonstrated weight (kg) and BMI (kg/m²) between ≥85th percentile and <95th percentile when compared to those with the presence of MetS where only 16.7% were between these values.

In contrast, for the boys:
On the other hand, in the group of adolescent males, it was observed, even among those without MetS, that 83% presented biochemical alterations (HDLc, triglycerides, and glycemia) and weight (kg) and BMI (kg/m2) higher or equal to the 95th percentile were observed in 55.5% of them. This confirms that even those without MeTS were much more compromised metabolically and nutritionally than the female adolescents.

8. In the conclusion session, authors mention the reduction in BMD and its effects on

Powered by Editorial Manager® and ProduXion Manager® from Aries Systems Corporation

peak bone mass, however, there are no data presented on BMD values or Z-scores in this cohort.
Answer: We apologize again that we did not present the data. We have now added the missing information to Table 1.

9.MetS increases bone resorption, however, CTX was lower in MetS group indicating lower bone resorption. It is eluded that most likely due to decrease bone remodeling. However, in chronic inflammation states of MetS and obesity osteoclast and bone resorption activities are significantly elevated as been shown in other adults studies and sites by the authors. In addition, authors showed that BMD negatively correlated with CTX. Can authors elaborate further on these rather unexpected findings?
Answer: We also believed that bone resorption should have increased, reflected by an increase in S-CTX, as documented in studies that evaluated adults. However, we should remember that adolescence is a time for increased bone mass and peak bone mass, as close to 42% of bone mass is added during the second decade of life. Therefore, bone formation should overcome resorption. Herein, we did not observe evolution as shown in adults. There was a reduction in the concentrations of all bone markers analyzed in both sexes among adolescents with MetS, suggesting an ? aggression to bone formation and resorption, possibly indicating a reduction in remodeling.

10.Table 4 appears to have a small shift in the values, which should be an easy edit.
Answer: Thank you, we have corrected this.

11.Table 5 is difficult to read, p values are missing in the comparison of Lumbar spine BMD with the bone makers; instead, r values are provided twice.
Answer: We appreciate your careful evaluation, and have made the corrections.

12.Table 6 is difficult to read and follow, recommend revision. There are multiple comparisons in the study, especially in table 6. It is not clear if correction for multiple comparisons was done. Can authors comment?
Answer: Dear reviewer, this is now Table 5, and it is possible to observe the linear correlations (Pearson) between bone biomarkers and the components of the Syndrome. The correlations demonstrate that each Mets component in isolation does not have a significant correlation with bone biomarkers. Thus, the few significant p values have now been highlighted in bold and with an asterisk.

13.Comment 3: Besides tables and results in the manuscript no additional data were provided. BMD data were not provided.
Answer: Thank you, we have made the corrections.

14.Comment 4: Discussion had comprehensive overview and review of available data on this and relevant topics. Many sentences are too long and difficult to follow. Discussion should be revised and edited for clarity.
Answer: We appreciate the considerations and the discussion has been revised and edited.

Line 59: "non-definitively demonstrated" - consider revision
Answer: We appreciate the comment. In the restructuring of the introduction this paragraph was removed.

14. Sentence in lines 261-268 is not clear and seems ambiguous. Please revise the point.
Answer: We accept your suggestion about ambiguity and have restructured the text.

15.In line 268 authors use BMTs, I am assuming it's a typo and they mean BMD. This should be clarified and corrected. There was another area where BMTs were used in place of intended BMD.
Answer: We apologize, but BMTs were used as an abbreviation for Bone Turnover Markers. However, in the current version the problem does not arise.

16.Lines 303-305: the sentence is incomplete. this needs a grammar check.
Answer: You are correct, we have corrected the paragraph.

Powered by Editorial Manager® and ProduXion Manager® from Aries Systems Corporation

17.The statements in lines 332-338 need references. The sentence in lines 417-424 is confusing and very long, needs revision.
Answer: Dear reviewer, we have added the respective references to lines 320-322 (Fernandes, Gonçalves, Brito, 2017; Cheung et al., 2013). In the other section, lines 352-355 have been reformulated: "However, in the present study there was a reduction in the concentrations of all bone markers analyzed in both sexes among adolescents with MetS, suggesting an aggression to bone formation and resorption, possibly indicating a reduction in remodeling".

aggression? what are the authors trying to say..

References:

Fernandes TAP, Gonçalves LML, Brito JAA. Relationships between Bone Turnover and Energy Metabolism. Journal of Diabetes Research.2017; 27: 1.

Cheung CL, Tan KC, Lam KS, Cheung BM. The relationship between glucose metabolism, metabolic syndrome, and bone-specific alkaline phosphatase: a structural equation modeling approach. J Clin Endocrinol Metab. 2013.9:3856-63.

Reviewer #2: General Comments

18.This appears to be a sub-study of a larger study looking at the impact of metabolic syndrome and its components on bone remodeling in adolescents. The authors present relevant data and the use of biochemical markers of bone turnover is appreciated given the age group and lack of research using bone markers in adolescents particularly in those who are classified as overweight or obese. This data would be a welcome addition to the literature.
Answer: Dear Reviewer, we appreciate your positive comments regarding our study.

20.There are number of general and specific comments that need to be addressed with respect to the interpretation of the bone remodeling data, editing of wording, and the discussion as it is currently written. For these reasons, a major revision is recommended.
As well, the Authors have a paper published in Bone (2014) examining MetS and its effects on bone mass, similarly using 271 participants and study design. Although that paper did not measure bone markers, it is not included in the current reference list even though sections of the methods could refer to it and make it clear if the 271 participants are similar between both papers. It appears the present study is a subset or additional analysis of a larger one that may have already been published. Are the current 84 participants from the original 271 participants or have any of them been additionally recruited? Can the authors provide any information or discussion on the use of or lack thereof a control group (normal weight non-MetS).
Answer: We apologize if it seemed that we had given prominence to our study, published in Bone (2014), as it appears among other references, in the Introduction (Nóbrega da Silva et al., 2014). There are few studies that address the theme in this age group and the current work is the subset of a larger work previously published, where we only analyzed the involvement of bone mineral density among adolescents with MetS. With respect to the inclusion of a control group with normal weight, it did not seem appropriate to conduct this study, considering the factors that could influence bone markers, when individuals are overweight and have MetS. In a previous publication (Mosca et al., 2017), we used a control group with adequate weight, when analyzing bone markers in obese individuals, without classifying them as MetS + or MetS –.

References:

Nóbrega da Silva V, Goldberg TBL, Mosca LN, Rizzo ACB, Teixeira AS, Corrente JE. Metabolic syndrome reduces bone mineral density in overweight adolescents. Bone. 2014; 66:1-7.

Mosca LN, Goldberg TBL, Silva VN, Kurokawa CS, Rizzo ACB, da Silva CC, Teixeira AS, Corrente JE. The impact of excess body fat on bone remodeling in adolescentes. Osteoporos Int. 2017. 28:1053–1062.

21.Much of the discussion is spent on studies and finding relating to OC, however that is not the only bone marker that was measured in the current study. CTx was mentioned briefly and there was no discussion provided to BAP. In addition, the

	authors provide meaningful information on types of diet, inflammation and cytokines in the discussion, but fail to use this information to support and make connections to the results of their study. Given that inflammatory cytokines or RANKL were not measured in the current study, I would recommend the authors not include these sections, unless they can connect it with the variables they did in fact measure. I would suggest speaking more to the specific MetS components, the number of them present and addressing S-CTx and BAP in the discussion.<br>Answer: We appreciate your suggestions, and have made adjustments to the text.<br><br>Specific Comments Abstract<br>21.1. Numerical values or results in terms of r- or p-values should be presented in the abstract.<br>Answer: Dear reviewer, we accept your suggestion and the abstract now contains the r and p-values.<br><br>Introduction<br>22.1. Define MetS, the conditions it can include, risk factors and how this relates to and presents in adolescents. How many MetS components do adolescents need to be classified as having MetS?<br>Answer: Dear Reviewer, the concept of MeTS and the recommendations proposed by the IDF, now read as follows in the methods (lines 160 to 165): "MetS was defined according to the criteria proposed by the IDF [Zimmet et al., 2007]. A subject was classified as having MetS if he/she presented central obesity, defined by a large waist circumference, and at least two of the four criteria shown in Table 1." In the new version, Table 1 was excluded (on request) and the text containing the explanations has been added to the manuscript.<br><br>Reference:<br>Zimmet P, Alberti KG, Kaufman F, Tajima N, Silink M, Arslanian S, Wong G, Bennett P, Shaw P, Caprio S, IDF CONSENSUS GROUP (2007). The metabolic syndrome in children and adolescents–an IDF consensus report. Pediatr Diabetes 8:299-306.<br><br>23.2. In the second paragraph of the introduction (lines 62-69), make reference to bone development papers. Only one reference is used at the end of the paragraph.<br>Answer: You are absolutely right. In the new version of the manuscript a wide review of the introduction has been carried out and some paragraphs have been adjusted with inclusions and removals.<br><br>24.3. Line 66, the use of the word "intense" to describe the imbalance between bone formation vs resorption due to growth should be rephrased.<br>Answer: Thank you for the suggestion, the alterations have been made.<br><br>25.4. Line 67-68, "under normal physiological conditions" and "dependent phenomena", is this in reference to growth in adolescents or a healthy adult? How bone remodeling is occurring during adolescence as a result of growth and maturation needs to be considered when describing this process.<br>Answer: Thank you for the suggestion, the alterations have been made.<br><br>26.5. Line 74 mentions a systematic review, please provide reference.<br>Answer: We have added the reference [3], thank you.<br><br>Reference:<br>Silva VND, Fiorelli LNM, Silva CC, Kurokawa CS, Goldberg TBL. Do Metabolic syndrome and its components have an impact on bone mineral density in adolescents? Nutrition Metabolism (Lond). 2017; 14:1-7.<br><br>27.6. Line 85, what is meant by "specialized literature"?<br>Answer: Thank you for the suggestion. We have rewritten the sentence to add clarity.<br><br>28.7. It is mentioned, line 80, that components of MetS are negatively correlated with BMD in adolescents. It may be worth including and referencing literature that has demonstrate negative impacts of excess weight in adolescents on BMD, BMC and/or bone strength, in addition to the incidence of increased fracture. There is some literature that has demonstrated negative correlations between BMI or excess weight

on marks of bone turnover. This can and should be used to support some of the findings related to MetS and bone properties.
Answer: Dear reviewer, we appreciate your comment. Our group is dedicated to understanding bone markers during adolescence and their relationship with excess weight, which has resulted in some publications.

References:
Nóbrega da Silva V, Goldberg TBL, Mosca LN, Rizzo ACB, Teixeira AS, Corrente JE. Metabolic syndrome reduces bone mineral density in overweight adolescents. Bone. 2014; 66:1-7.
Mosca LN, Goldberg TBL, Silva VN, Kurokawa CS, Rizzo ACB, da Silva CC, Teixeira AS, Corrente JE. The impact of excess body fat on bone remodeling in adolescentes. Osteoporos Int. 2017. 28:1053–1062.

Silva VND, Fiorelli LNM, Silva CC, Kurokawa CS, Goldberg TBL. Do Metabolic syndrome and its components have an impact on bone mineral density in adolescents? Nutrition Metabolism (Lond). 2017; 14:1-7.

The impact of excess body fat on bone remodeling was evaluated in overweight, obese, and extremely obese adolescents. In adolescents with excess weight, it was observed that the higher the bone mineral content and bone mineral density values, the lower the levels of the biomarkers. Nutritional imbalances through excess had a negative effect on bone formation in this stage of life. In girls with excess weight, the biomarkers were higher in the 10 to 13-year age group and no significant differences were observed between groups according to nutritional status. In boys, the levels were higher in those aged 13 to 15 years. According to nutritional status, significant differences were only observed in mean S-CTx for the age groups of 10–15 years, with higher levels between overweight and obese adolescents aged 10–12 years and between obese and extremely obese adolescents aged 13–15 years. In girls, significant negative correlations were observed between lean mass, fat mass, and fat percentage and each of the three bone markers studied. There was no correlation between lean mass or fat mass and the three biomarkers in boys. The biomarker trends demonstrated across the age groups follow the age trends for growth velocity. In conclusion, the higher the fat percentage and fat mass in girls, the lower the levels of the biomarkers, indicating that excess body fat has a negative effect on the evolution of these markers during adolescence. (Mosca et al., 2017).

Our research group has been investigating explanations about biomarkers and bone remodeling in adolescents with and without MetS, especially considering the lack of data in the literature on this population of adolescents.

29.1. Please refer to general comment as it pertains to previously published paper that could be referenced in this section of the document.
Answer: Dear reviewer, we have added our previous studies, thank you.

References:
Nóbrega da Silva V, Goldberg TBL, Mosca LN, Rizzo ACB, Teixeira AS, Corrente JE. Metabolic syndrome reduces bone mineral density in overweight adolescents. Bone. 2014; 66:1-7.

Mosca LN, Goldberg TBL, Silva VN, Kurokawa CS, Rizzo ACB, da Silva CC, Teixeira AS, Corrente JE. The impact of excess body fat on bone remodeling in adolescentes. Osteoporos Int. 2017. 28:1053–1062.

Silva VND, Fiorelli LNM, Silva CC, Kurokawa CS, Goldberg TBL. Do Metabolic syndrome and its components have an impact on bone mineral density in adolescents? Nutrition Metabolism (Lond). 2017; 14:1-7.

30.2. Final paragraph of section 2.1 (lines 132-136) describe how participants were matched and compare to the 271 subjects. Consider moving this to the first paragraph of section 2.1 to make it clear earlier in the paper how participants were matched for the study analysis.
Answer: Dear reviewer, we appreciate your suggestion, which was fully accepted.

31.3. Why did the authors choose to match participants by chronological age, bone age, and pubertal stage? Please provide reference or justification for using these matching criteria and perhaps for not including anthropometric measures.
Answer: Dear reviewer, the choice to match individuals by these variables resulted from the great differences observed between pubescent children in different stages of growth and physical maturity (early, middle, and late puberty). In addition, regarding the definition of metabolic syndrome in adolescents according to International Diabetes Federation criteria, pubescents aged 10 to 16 are analyzed as a single group, with only the percentile of waist circumference varying and the cutoff points for the other biochemical markers being similar for all criteria. In addition, from a statistical point of view, non-matching could generate bias in the explanations about the MetS outcome itself if the sample was not minimally homogenized.

32.4. Are the authors aware and able to report how many of the female participants were pre vs. post-menarcheal, or had all female participants reached menarche?
Answer: Dear reviewer, each individual among these 271 adolescents was enrolled and followed up at our Adolescence Service. So we can assure you that, as explained in the text, they were included in the sample with care.
As more than 60% of the adolescents, both those in the MetS + and MetS- groups, were in late puberty, we can suggest that they had already menstruated (see Table 1 in the new version). However, we do not have these data to confirm our assumption, as it would be necessary to ask each individual included.

33.5. It is not clear how the information that was used for the inclusion criteria was collected. Was this done by interview, survey, or a specific questionnaire. Specifically, how was the nutritional information collected?
Answer: Dear Reviewer, I believe that the answer to the previous question will resolve any doubts. In the text we have written (line 94): “on the occasion of their first visit to the Adolescent Unit of the Botucatu Medical School University Hospital (SP, Brazil), between 2011 and 2013”.

34.6. Line 141, potential editing error in sentence – “utilized to classify nutritional status”.
Answer: Dear reviewer, we accept your suggestion and have edited the sentence.

35.7. Like 143 makes refence to growth curves. Are these based on WHO or CDC curves?
Answer: They are based on CDC growth charts.

Reference:
Kuczmarski RJ, Ogden CL, Guo SS, Grummer-475 Strawn LM, Flegal KM, Mei Z, Wei R, Curtin LR, Roche AF, Jhonson CL 2000. CDC Growth Charts for the United States: Methods and Development. Vital Health Stat. 2002; 11 246: 1-190.

Statistical Analysis/Results

36.1. Given that DXA was used, is it possible for the authors to provide data on lean and fat mass, or as a percentage, for the participants. Is there BMC data that can be reported?
Answer: Dear Reviewer, we have all these data in our parent spreadsheet. However, BMC data was also not presented in the study published in Bone (Nóbrega da Silva et al., 2014), as we have already commented to Reviewer 1. In that study, the analysis included all 271 adolescents, as shown in the table below.

Regarding fat mass and percentage, we have already published the association with bone markers, identifying a negative association, indicating that the greater the fat mass or its percentage, the lower the results of the concentrations of the markers. In the conclusion of this paper we have written: The higher the fat percentage and fat mass in girls, the lower the levels of the biomarkers, indicating that excess body fat has a negative effect on the evolution of these markers during adolescence.

References:
Nóbrega da Silva V, Goldberg TBL, Mosca LN, Rizzo ACB, Teixeira AS, Corrente JE. Metabolic syndrome reduces bone mineral density in overweight adolescents. Bone.

2014; 66:1-7.

Mosca LN, Goldberg TBL, Silva VN, Kurokawa CS, Rizzo ACB, da Silva CC, Teixeira AS, Corrente JE. The impact of excess body fat on bone remodeling in adolescents. Osteoporos Int. 2017. 28:1053–1062.

37.2. Table 2, the n-values for the Tanner stages do not add up to be the n-values for the respective group.
Answer: You are right, in some teenagers we did not obtain Tanner stages, however, we obtained bone age from all of them and we know that there is a strong correlation between these events.

38.3. It is not clear what Tanner stages the authors are using to define participants as early, mid or late puberty. Please define this in the maturation section of the methods.
Answer: We used the classification proposed by Marshall & Tanner, however, for the number of adolescents in each group the following were adopted, Breast 1- 2 considered early puberty, Breast 3 mid puberty, and Breast 4 and 5 late puberty, as well as in genitals in male adolescents (Table 1 in this new version of the manuscript).

Reference:
Finkelstein JW. The endocrinology of the adolescent. Symposium of adolescent medicine. Pediatrics Clinic of North America.1980; 27:53-69.

39.4. Statistical analysis section states (Line 215-218) than an ANOVA was conducted to compare bone markers with the number of MetS components, however results are not presented in the document.
Answer: Dear reviewer, you are absolutely right, we have removed this analysis from our study and present it in the new version of the manuscript.

40.5. In terms of analysis, the age range (10-16yrs) in addition to the inclusion of all tanner stages into the analysis may be problematic and contributing to some of the correlations and findings.
Answer: Dear reviewer, we agree and also considered this a problematic situation when we outlined the study. However, the IDF uses this group of individuals to perform the classification of those with or without MetS, in addition to the matching that took into account bone and chronological age and the pubertal stages.

41. A) Bone marker concentrations change with maturity and there have been studies to show varying levels by tanner stage. I would expect to see negative correlations since BMD increases and bone marker concentrations decrease with age/maturation. It is not clear from the data and subsequent discussion why this relationship is significant in females and more so in those that have MetS. Is the number of MetS components, or a specific component, or perhaps their excess weight (percent body fat) a factor? Is it related to their maturation? Looking at the data more females are in late puberty compared to boys. Is the menarcheal status between the female MetS and non-MetS participants similar?
Answer: Dear Reviewer, all your questions are highly thought-provoking and we reflected on a number of them while writing our discussion. Our research group started on the topic by analyzing bone density and mineral content and bone markers in eutrophic adolescents, male and female. Next, we analyzed the effects of obesity on bone density and bone markers in adolescents. Thus, we are aware of this negative relationship that the reviewer so adequately points out (references already presented). However, in order to avoid doubts that the age effect alone was responsible for the reduction in bone markers evidenced in the current study, we present in the discussion the following extract (line 261- 276):
"Researchers have highlighted the importance of understanding the evolution of BMD and alterations in the levels of bone biomarkers during adolescence (Silva et al., 2012; Fortes et al., 2014). Fortes et al. (2014), evaluating BMD and its relationship with bone markers in a sample of 101 eutrophic female adolescents of 9 to 20 incomplete years, observed an expressive increase in BMD according to the progression of chronological and bone age. With respect to indicators of bone metabolism, higher mean biomarker levels (BAP=110.12 U/L, OC=26.49ng/mL, S-CTx=1.58 ng/mL) were found when the adolescents were in stage 3 of breast development and in early adolescence (11 to 12 years). The levels of bone biomarkers were lower at the end of adolescence

Powered by Editorial Manager® and ProduXion Manager® from Aries Systems Corporation

(BAP=31.70 U/L, OC=4.98ng/mL, S-CTx=0.44 ng/mL). Similar results were recently published with the follow-up of 2,416 children and adolescents, from 3 months to 17 years of age, analyzing bone markers Osteocalcin, PINP, and CTX-I, 317 with obesity and the other eutrophic (Geserick et al., 2020). During puberty, the authors demonstrated that the highest levels of bone markers occurred from 10 to 11 years (girls, Tanner 3) and 13 years (boys, Tanner 3–4). Results very close to those presented by Fortes et al. (2014) regarding age and pubertal stage.
In addition, our current group of teenagers included were between 10-16 years old, with an average CA of around 12 years for girls and 13 years for boys and BA of around 13 years for both.
(Line: 277 – 289): "In male adolescents, Silva et al. (2012) studied a sample of 87 eutrophic adolescents aged 10 to 18 years and concluded that serum biomarker levels differed significantly among age groups. The highest concentration of the biomarkers was observed between 13 and 15 years of age (BAP = 155.50U/L, OC = 41.63 ng/mL, S-CTx = 2.09 ng/mL) and the lowest concentration between 16 and 18 years (BAP= 79.80U/L, OC = 27.80 ng/mL, S-CTx = 1.60 ng/mL). Within this context, proportionally higher levels of bone biomarkers would be expected in the adolescents in this present study, since the mean CA of girls with or without MetS was around 12 years of age and BA, around 13 years of age, while in boys both CA and BA were around 13 years of age. The concentrations obtained among adolescents without MetS in the present study were close to those observed among the eutrophic individuals analyzed in the studies cited (Silva et al., 2012; Fortes et al., 2014). However, a significant reduction in both biomarkers of bone formation and resorption was observed in the group of adolescents with MetS compared to the matched group without MetS".

With respect to the negative correlations, this was part of one of the responses to the Editor (Comment 3):

References:
Silva CC, Kurokawa CS, Nga HS, Moretto MR, Dalmas JC, Goldberg TBL. Bone metabolism biomarkers, body weight, and bone age in healthy Brazilian male adolescents. J Pediatr Endocrinol Metab. 2012; 25: 479-484.

Fortes CMT, Goldberg TBL, Kurokawa CS, Silva CC, Moretto MR, Biason TP, Teixeira AS, Nunes HRC. Relação entre as idades cronológica e óssea e o estágio puberal das mamas com os biomarcadores ósseos e a densidade mineral óssea em adolescentes. J Pediatr (Rio J). 2014; 90:624-631.

Geserick M, Vogel M, Eckelt F, Schlingmann M, Hiemisch A, Baber R, Thiery J, Korner A, Kiess W, Kratzsch J. Children and adolescents with obesity have reduced serum bone turnover markers and 25-hydroxyvitamin D but increased parathyroid hormone concentrations - Results derived from new pediatric reference ranges. Bone. 2020;132:115-124.

42. B) Because of the changing bone marker concentrations with maturation it is not uncommon for studies to separate participants into groups based on tanner staging. Have the authors considered creating 2 tanner groups to help minimize maturation effects that come with having all tanners 1-5 grouped together?
Answer: Dear reviewer, we understand your concern and believe that our comments on the previous questions demonstrate why we did not perform the analysis based on pubertal stages. In addition, there is no recommendation for the classification of MetS by pubertal stages in adolescents, the 10-16 age group being considered as a single group (IDF, 2007).

Reference:
Zimmet P, Alberti KG, Kaufman F, Tajima N, Silink M, Arslanian S, Wong G, Bennett P, Shaw P, Caprio S, IDF CONSENSUS GROUP (2007). The metabolic syndrome in children and adolescents–an IDF consensus report. Pediatr Diabetes 8:299-306.
43.C) As well, given that BMD is affected by body size, have the authors considering or tried adjusting their analysis by controlling for various anthropometric measures (i.e. height)?
Answer: Dear reviewer, reviewer 1 asked a similar question. We ask you to please consider the answer to question 4.

Powered by Editorial Manager® and ProduXion Manager® from Aries Systems Corporation

Discussion
44.1. Line 256 – in reference to the “behavior”’ of bone biomarkers. Please rephrase to be clearer as to what the results were.
Answer: Dear reviewer, we have made the adjustments in the new version of the manuscript.

45.2. Line 259 – states that BMD was negatively correlated with all bone markers in adolescents with MetS. However, this was only the case with females and not males. Please correct this statement.
Answer: Dear reviewer, you are right, we have added that the correlations were negative only in girls (First paragraph of the discussion).

46.3. Lines 261-268 – authors discuss collinearity of the bone markers. What is the purpose of this finding and how does it relate to the literature and data?
Answer: Dear reviewer, you are right, we have deleted this paragraph.

47. 4. Line 268 – what is BMT?
Answer: Dear reviewer, Bone markers of turnover (BMT). We are sorry we did not write this in full.

48. 5. Provide reference at end of sentence (line 271)
Answer: Dear reviewer, we have added the reference [30] to the end of the sentence in this new version of the manuscript.

Reference:
Weaver CM, Gordon CM, Janz KF, Kalkwarf HJ, Lappe JM, Lewis R, Okarma M, Wallace TC, Zemel BS. The national osteoporosis foundation's position statement on peak bone mass development and lifestyle factors: a systematic review and implementation recommendations. Osteoporos Int. 2016; 27:1281-1386.

49. 6. Paragraph (Line 277-295) – Authors discuss bone marker levels by age and tanner stage. Please be specific if you are referring to males vs. females in those studies. And which of these studies used participants with MetS. As well, how does these studies and their grouping compare given that the current study includes all tanner stages into one group. Please refer to comment 5b in previous section.
Answer: Dear reviewer, the answers to this comment can be found in other questions (41A and 42B). We emphasize that the studies cited were developed for eutrophic girls and boys, independently. We did not detect articles that evaluate bone turnover markers in adolescents with MetS in the literature. Some authors have analyzed Osteocalcin and its relationship with some MetS components, and these are mentioned in the discussion.

50.7. Line 307 – Unclear what the results of Tan et al were and how they compare to the current findings. Please elaborate.
Answer: Dear reviewer, this paragraph has been reviewed and adjusted, highlighting the negative correlation between OC and MetS that occurred for males. Tan et al. (2011) evaluated 2,344 Chinese men between 20 and 69 years of age and found a negative association between OC levels and the presence of MetS. These results agree with our findings, however, in our results the reductions in OC were significant and negative only in female adolescents with MetS.

Reference:
Tan A, Gao Y, Yang X, Zhang H, Qin X, Mo L, Peng T, Ning X, Mo Z. Low serum osteocalcin level is a potential marker for metabolic syndrome: results from a Chinese male population survey. Metabolism. 2011; 60:1186-1192.

51. 8. Line 318 – what do the authors mean by “biochemical alterations”?
Answer: We ask the Reviewer to please note the response to Comment 7 to Reviewer 1.

52.9. Paragraph (lines 324-327) – small paragraph talking about OC. Consider reorganizing and placing it within previous paragraph where authors discuss OC. As well, please provide clearer discussion of findings when comparing current study results with study using eutrophic children, when such a group was not used in the

	current study.<br>Answer: Dear Reviewer, we appreciate your suggestion and have deleted the paragraph, as the comparison was actually made with eutrophic children.<br><br>53.10. Lines 334-335 – please provide references<br>Answer: Dear Reviewer, we agree and have reorganized the entire paragraph including references.<br><br>54.11. It is not clear the connection the authors are trying to make between different types of OC and various other physiological markers, and how this relates to the finding of the current study.<br>Answer: Dear Reviewer, we agree and have reorganized the text of the discussion in this new version of the manuscript.<br><br>55.12. Please provide discussion on limitations for the current study. Please include some methodological limitations of using DXA measurement with children and adolescents.<br>Answer: Dear Reviewer, we have included the study limitations at the end of the discussion section. Details of the evaluation of bone mineral density are highlighted in the methods section.<br><br>56.13. Line 426 and 427 – the authors mention "raising new hypotheses" and suggesting possible "design for future studies", although hypotheses were not presented within the document. Can the authors please include some examples for potential future studies or considerations.<br>Answer: Dear Reviewer, we have included this information in the new version of the manuscript. Thank you again for the detail of the review and we hope to have met your expectations.
**Additional Information:**	
**Question**	**Response**
**Financial Disclosure**<br><br>Enter a financial disclosure statement that describes the sources of funding for the work included in this submission. Review the submission guidelines for detailed requirements. View published research articles from *PLOS ONE* for specific examples.<br><br>This statement is required for submission and **will appear in the published article** if the submission is accepted. Please make sure it is accurate.	Supported by FAPESP (Fundação de Amparo à Pesquisa do Estado de São Paulo) – Grants (07/07731-0, 2011/05991-0 and 2015/04040-2) and the Pro-Rector for Research of UNESP.

Powered by Editorial Manager® and ProduXion Manager® from Aries Systems Corporation

**Unfunded studies**<br>Enter: *The author(s) received no specific funding for this work.*<br><br>**Funded studies**<br>Enter a statement with the following details:<br>• Initials of the authors who received each award<br>• Grant numbers awarded to each author<br>• The full name of each funder<br>• URL of each funder website<br>• Did the sponsors or funders play any role in the study design, data collection and analysis, decision to publish, or preparation of the manuscript?<br>• **NO** - Include this sentence at the end of your statement: *The funders had no role in study design, data collection and analysis, decision to publish, or preparation of the manuscript.*<br>• **YES** - Specify the role(s) played.<br><br>* typeset	
**Competing Interests**<br><br>Use the instructions below to enter a competing interest statement for this submission. On behalf of all authors, disclose any competing interests that could be perceived to bias this work—acknowledging all financial support and any other relevant financial or non-financial competing interests.<br><br>This statement **will appear in the published article** if the submission is accepted. Please make sure it is accurate. View published research articles from *PLOS ONE* for specific examples.	The authors have declared that no competing interests exist.

**NO authors have competing interests**<br><br>Enter: *The authors have declared that no competing interests exist.*<br><br>**Authors with competing interests**<br><br>Enter competing interest details beginning with this statement:<br><br>*I have read the journal's policy and the authors of this manuscript have the following competing interests: [insert competing interests here]*<br><br>* typeset	
**Ethics Statement**<br><br>Enter an ethics statement for this submission. This statement is required if the study involved:<br><br>• Human participants<br>• Human specimens or tissue<br>• Vertebrate animals or cephalopods<br>• Vertebrate embryos or tissues<br>• Field research<br><br>Write "N/A" if the submission does not require an ethics statement.<br><br>General guidance is provided below. Consult the submission guidelines for detailed instructions. **Make sure that all information entered here is included in the Methods section of the manuscript.**	The study was approved by the Ethics Committee of the Botucatu Medical School (FMB-UNESP) (Approval No. 1.399.709 /2016).

Powered by Editorial Manager® and ProduXion Manager® from Aries Systems Corporation

**Format for specific study types**<br><br>**Human Subject Research (involving human participants and/or tissue)**<br>• Give the name of the institutional review board or ethics committee that approved the study<br>• Include the approval number and/or a statement indicating approval of this research<br>• Indicate the form of consent obtained (written/oral) or the reason that consent was not obtained (e.g. the data were analyzed anonymously)<br><br>**Animal Research (involving vertebrate animals, embryos or tissues)**<br>• Provide the name of the Institutional Animal Care and Use Committee (IACUC) or other relevant ethics board that reviewed the study protocol, and indicate whether they approved this research or granted a formal waiver of ethical approval<br>• Include an approval number if one was obtained<br>• If the study involved *non-human primates*, add *additional details* about animal welfare and steps taken to ameliorate suffering<br>• If anesthesia, euthanasia, or any kind of animal sacrifice is part of the study, include briefly which substances and/or methods were applied<br><br>**Field Research**<br><br>Include the following details if this study involves the collection of plant, animal, or other materials from a natural setting:<br>• Field permit number<br>• Name of the institution or relevant body that granted permission	
**Data Availability**<br><br>Authors are required to make all data underlying the findings described fully available, without restriction, and from the time of publication. PLOS allows rare exceptions to address legal and ethical concerns. See the PLOS Data Policy and FAQ for detailed information.	Yes - all data are fully available without restriction

A Data Availability Statement describing where the data can be found is required at submission. Your answers to this question constitute the Data Availability Statement and **will be published in the article**, if accepted.<br><br>**Important:** Stating 'data available on request from the author' is not sufficient. If your data are only available upon request, select 'No' for the first question and explain your exceptional situation in the text box.<br><br>Do the authors confirm that all data underlying the findings described in their manuscript are fully available without restriction?	
**Describe where the data may be found in full sentences. If you are copying our sample text, replace any instances of XXX with the appropriate details.**<br><br>• If the data are **held or will be held in a public repository**, include URLs, accession numbers or DOIs. If this information will only be available after acceptance, indicate this by ticking the box below. For example: *All XXX files are available from the XXX database (accession number(s) XXX, XXX.*).<br>• If the data are all contained **within the manuscript and/or Supporting Information files**, enter the following: *All relevant data are within the manuscript and its Supporting Information files.*<br>• If neither of these applies but you are able to provide **details of access elsewhere**, with or without limitations, please do so. For example:<br><br>*Data cannot be shared publicly because of [XXX]. Data are available from the XXX Institutional Data Access / Ethics Committee (contact via XXX) for researchers who meet the criteria for access to confidential data.*<br><br>*The data underlying the results presented in the study are available from (include the name of the third party*	All relevant data are within the manuscript and its Supporting Information files.

and contact information or URL).<br>• This text is appropriate if the data are owned by a third party and authors do not have permission to share the data.<br><br>* typeset	
Additional data availability information:	Tick here if the URLs/accession numbers/DOIs will be available only after acceptance of the manuscript for publication so that we can ensure their inclusion before publication.

Powered by Editorial Manager® and ProduXion Manager® from Aries Systems Corporation

Manuscript Click here to access/download;Manuscript;Manuscript_Goldberg et al.

Impact of metabolic syndrome and its components on bone remodeling in adolescents

Metabolic syndrome and bone remodeling

Valéria Nóbrega da Silva[a], Tamara Beres Lederer Goldberg[a*], Carla Cristiane Silva[b], Cilmery Suemi Kurokawa[c], Luciana Nunes Mosca Fiorelli[a], Anapaula da Conceição Bisi Rizzo[a], José Eduardo Corrente[d]

[a]Postgraduate Program in Gynecology, Obstetrics, and Mastology, Discipline of Adolescent Medicine, Department of Pediatrics, Botucatu Medical School, UNESP, São Paulo State University, Botucatu, SP, 18618-970, Brazil; E-mail addresses: valerianutry@gmail.com; tamara.goldberg@unesp.br; lunutricionista@yahoo.com.br; anapaula.rizzo@uol.com.br

[b]Department of Physical Education, University of North Paraná, Jacarezinho, Paraná, Brazil; E-mail address: ccsilva@uenp.edu.br

[c]Clinical and Experimental Pediatric Research Center, Department of Pediatrics, Botucatu Medical School, UNESP, São Paulo State University, Botucatu, SP, Brazil; E-mail address: kurokawa@fmb.unesp.br

[d] Department of Statistics, Biosciences, UNESP, São Paulo State University, Botucatu, SP, Brazil; E-mail address: jecorren@ibb.unesp.br

**Corresponding author:*

Department of Pediatrics, Botucatu Medical School, UNESP, São Paulo State University, Brazil. (Tel.: +55-14-3811-6274/+55-14-3811-6083)

e-mail. tamara.goldberg@unesp.br

Abstract

Osteoporosis and metabolic syndrome (MetS) are diseases that have serious public health consequences, reducing the quality of life of patients and increasing morbidity and mortality, with substantial healthcare expenditures.

Objective

To evaluate the impact of MetS on bone mineral density (BMD) and biochemical markers of bone formation and resorption in adolescents with excess weight.

Method

A descriptive and analytical cross-sectional study that evaluated 271 adolescents of both sexes (10 to 16 years). From the total sample, 42 adolescents with excess weight and the presence of MetS (14%) were selected. A further 42 adolescents with excess weight and without MetS were selected and matched for chronological age, bone age, and pubertal developmental criteria to those with MetS, for each sex. The adolescents were submitted to anthropometry, blood pressure measurement, and biochemical tests, as well as evaluation of BMD and the bone biomarkers osteocalcin (OC), bone alkaline phosphatase (BAP), and carboxy-terminal telopeptide (S-CTx).

Results

The adolescents with excess weight and MetS exhibited a significant decrease in BMD and concentrations of BAP, OC, and S-CTx compared to the matched group, except for OC in boys. A negative and significant correlation was observed between total body BMD and BAP ($r = -0.55568$; $p = 0.005$), OC ($r = -0.81760$; $p = <.000$), and S-CTx ($r = -0.53838$; $p = 0.011$) in girls.

Conclusion

The presence of MetS is detrimental to the bone mineral density (BMD) and biochemical markers of bone formation and resorption in adolescents with excess weight.

Keywords: Adolescents; Bone, Metabolic Syndrome

Introduction

Osteoporosis and metabolic syndrome (MetS) are diseases that have serious public health consequences, reducing the quality of life of patients and increasing morbidity and mortality, with substantial healthcare expenditures [1]. The occurrence of MetS in adolescents is associated with an increased risk of cardiovascular diseases, psychosocial problems, abnormal glucose metabolism, nonalcoholic fatty liver disease, polycystic ovary syndrome, obstructive sleep apnea, mental disorders, orthopedic complications, and disorders of motor development [2]. In addition, to these conditions, although a possible relationship between this syndrome and bone mass has been demonstrated, the number of studies addressing this topic in adolescents is still small [3]. Despite the paucity of evidence, the results of some studies reveal that MetS is negatively correlated with bone mass and that some components of MetS are negatively correlated with BMD in adolescents [4-8].

In adolescence, the period of pubertal development is important for the acquisition of bone mineral content. Especially in this phase of life, which is dominated by physical growth, there is an imbalance between bone formation and resorption, and the biochemical formation markers are correlated with the rate of growth [9]. Under normal physiological conditions, bone resorption and formation are dependent phenomena and the predominance of one over the other may lead to the gain or loss of bone mass [10].

The relationship of bone mass with the occurrence of MetS in children and adolescents has been a subject of interest to some researchers [3- 6, 11], since impairment in bone acquisition during this period can lead to “suboptimal” peak bone mass and increase the risk of developing osteopenia/osteoporosis and fractures in old age [7]. A recent systematic review performed a critical analysis of studies that focused on the period of adolescence, evaluating the influence of MetS and its components on bone mineral density (BMD) in this age group [3].

In adolescents, the relationship between MetS and markers of bone formation and resorption is not fully known. However, some lines of evidence indicate a negative correlation between serum levels of the bone marker osteocalcin and metabolic risk factors in obese children and adolescents [12]. In view of the results already published [3, 5,6], the objective of this study was to evaluate the impact of MetS and each of its components on BMD and on biochemical markers of bone formation and resorption in adolescents.

2. Material and methods

2.1 Study Design and Participants

This was a descriptive and analytical cross-sectional study involving a sample of 271 adolescents with excess weight [5]. For methodological reasons, the final sample consisted of only 84 adolescents because of the need to analyze two matched groups with and without MetS. The groups were matched for chronological age, bone age, and pubertal stage for each sex. Our sample of 84 adolescents did not differ from that of the 271 initially included subjects. The sample of adolescents of both sexes with and without MetS and with excess weight was selected based on the availability of care for new cases

at the Adolescent Outpatient Clinic of the University Hospital, Botucatu Medical School (SP, Brazil) on the occasion of their first visit. Data were collected from March 2011 to March 2013. The adolescents were invited to participate in the study at the time of their visit to the outpatient clinic and a written informed consent form was signed by the adolescents and by their parents or legal guardians. The study was approved by the Research Ethics Committee of the Botucatu Medical School (Approval N°. 1.399.709 /2016).

The inclusion criteria were as follows: 10 to 16 years of age, overweight (BMI ≥85th percentile for sex and age), and between the 5th and 95th age- and sex-appropriate height percentiles [13]. Adolescents were excluded from this study for the following reasons: weight exceeding 100 kg (densitometry was not feasible for these potential subjects), a history of prematurity, a history of fracture in the previous two years, prolonged therapy with corticosteroids, use of calcium and/or iron supplements in the 12 months preceding this research, presence of chronic diseases, use of drugs that negatively affect bone metabolism such as medroxyprogesterone acetate, anticonvulsants, or antacids containing aluminum, consumption of an exclusive high-fiber diet containing above age-appropriate recommendations (>26 g/day for female adolescents or >31 g/day for male adolescents (9–13 years) or >38 g/day for male and female adolescents 14–16 years) [14], a vegan or vegetarian diet, caffeine intake >300 mg/day (>3 cups of coffee per day) [15] consumption of more than 500 mL/day of soft drinks [16,17], and lack of daily consumption of dairy products. Furthermore, the adolescents did not smoke or drink alcohol, were not engaged in any extracurricular sporting activities, and participated in physical education classes in their schools for no more than 2 h/week. Other exclusion criteria were the presence of metabolic, endocrine, or genetic diseases (as verified by a

history of current disorders, general and specific physical examinations, laboratory or radiological procedures), any changes in menstrual cycle (oligo/amenorrhea, clinical or biochemical hyperandrogenism) that could suggest the presence of polycystic ovary syndrome, use of combined oral contraceptives, and pregnancy in girls. These exclusion criteria were established in order to prevent other events or situations described in the literature from interfering with the increase in bone mass in these adolescents. If adolescents presented any mental alterations or used medication to treat mental disorders they were not included in the study.

Adolescents of both sexes were excluded if they failed to attend all scheduled appointments for anthropometric measurements or blood sampling or if they did not follow the established sample collection procedures; e.g., if they did not fast for at least 10 h prior to collection.

2.2 Anthropometric Assessment and Blood Pressure Measurement

Anthropometric measures of weight (kg) and height (m) were obtained as recommended by the National Health and Nutrition Examination Survey [17], from which the body mass index was calculated (BMI, kg/m^2), utilized to assess nutrition status. The BMI percentiles were obtained with Epi Info 3.5.1 Software, which uses sex and age growth curves as references [18]. Adolescents with a BMI between the 85th and 95th percentile were classified as overweight, between the 95th and 99th percentile as obese [19], and above the 99th percentile as extremely obese [20]. Waist circumference (cm) was obtained at the midpoint between the last rib and the iliac crest at the end of a regular expiration [21]. Blood pressure was measured with the adolescent in a sitting position after a 5-min rest. The mean of three measurements with 3-min intervals was used.

Measurements were performed on the right arm by auscultation, using a calibrated mercury sphygmomanometer and a cuff appropriate for the arm circumference [22].

2.3 Evaluation of Sexual Maturation and Skeletal Maturation Stages

Sexual maturity was evaluated by an experienced physician through visual breast evaluation (females) or genital observation (males) and the results reported using the Tanner scale [23,24], and grouped according to Finkelstein et al. [25] into early puberty, mid-puberty, and late puberty.

To evaluate skeletal maturation, bone age was obtained by the GP method [26] in which the hand and wrist radiographs were compared to the atlas. This examination was performed under the supervision and guidance of a specialized professional who was unaware of the anthropometric and densitometric characteristics of the adolescents (blind evaluator).

2.4 Biochemical Evaluation

Blood samples were obtained in the morning after a 10-h fast, between 7:00 am and 9:00 am, and all storage precautions were taken. The samples were collected by well-trained laboratory technicians and evaluated at the Central Laboratory. Dry chemistry was used to assess HDL-c, triglycerides, and fasting glucose in a Vitros 950 analyzer (Johnson and Johnson) according to the manufacturer's instructions. Values are expressed as mg/dL. MetS was defined according to the criteria proposed by the IDF [27]. The adolescent aged 10 to 16 years were classified as having MetS if they presented central obesity, defined by a large waist circumference (>90th percentile of WC by sex and age), and at least two of the four criteria: (TG ≥150 mg/dL, HDL <40 mg/dL, SBP ≥130 mmHg

or DBP ≥85 mmHg, and fasting blood glucose ≥100 mg/dL or a type 2 diabetes diagnosis).

any with T2DM/ impaired glucose tolerance?

The blood samples were collected by venous puncture and centrifuged for 15 min at 1500*g* for the separation of serum. The serum samples were stored at -70°C until the time of analysis of the biomarkers (BAP, OC, and S-CTx). BAP and intact OC were measured using an assay from Metra™ Biosystems (San Diego, CA, USA), with intra- and inter-assay coefficients of variation of 8 and 7.6%, respectively. S-CTx was quantified by an electrochemiluminescence immunoassay (ECLIA) using the Elecsys beta-Cross Laps serum assay in an automated Elecsys device (Roche™, Indianapolis, IN, USA). The inter-assay coefficient of variation was 5%.

2.5 Evaluation of Bone Mineral Density

BMD is not height adjusted, though groups didn't differ in height. I would include this in the methods. Also, I would suggest to the authors to include and do analysis of BMD z-scores.

The adolescents underwent bone densitometry through dual-energy X-ray absorptiometry (DXA) (Hologic QDR 4500 Discovery A, Hologic, Inc., Bedford, MA, USA). The bone mass results were analyzed using appropriate pediatric software. Bone mineral content is expressed in g and BMD in g/cm^2. Measurements were obtained from the L1–L4 lumbar spine region, the total proximal femur including the femoral neck and trochanteric and intertrochanteric regions, and total and subtotal body (whole body less head). Whole body densitometry was performed to obtain total bone mineral content, BMD, and whole body composition. The effect of body size on bone mass can lead to misinterpretations when comparing individuals of different heights and body compositions. In the current study, we sought to minimize body size-induced biases in bone mass estimates by using BMD transformed by body weight (g/cm^2/kg body weight) [28]. All assessments were performed by only one technician trained for this purpose who had no knowledge of the

research. In addition, the instructions of the manufacturer of the device and the standards recommended by the International Society for Clinical Densitometry [29] were followed.

The DXA instrument was calibrated by daily scanning of a hydroxyapatite spine phantom. Machine drift was not observed during the study. The coefficient of variation was estimated from repeated measurements (twice) obtained from 30 patients representative of the clinic's patient population for all regions mentioned (lumbar spine and total body) after each patient had been repositioned before scanning. The results demonstrated CVs of 0.6% and 1.3% for the lumbar spine and whole body, respectively. All evaluations were performed by the same blinded, experienced technician, who also performed the densitometry examinations.

2.6 Statistical Analysis

Data were stored in Excel and analyzed using SAS for Windows v.9.2 software. Normality of the quantitative variables was verified by the Shapiro-Wilk test. For characterization of the sample, descriptive analysis of the data was performed by calculating the distribution of frequencies and measures of central tendency and dispersion. The Student t-test was used for the comparison of chronological and bone age,
should this be BMD?
anthropometric variables (weight, height, BMI), BDM and transformed BMD, and bone markers between adolescents with and without MetS and its components, stratified by sex. The frequencies of pubertal stages according to sex were compared between adolescents with and without MetS using the chi-square test.

Pearson's correlation was used to evaluate the correlation between serum levels of the markers and variables related to BMD and MetS components. All analyses were

performed separately for each sex. Statistical significance was accepted for all analyses when p≤0.05.

3. Results

would suggest to include SD

Forty-eight (57%) of the 84 adolescents were female. The mean age was 12 years among girls and 13 years among boys, both with an average bone age advancement of one year. Among the adolescents, 77.4% were in mid- and late puberty. The adolescents with MetS had higher weight and BMI than those without MetS (p≤0.05). No significant differences in chronological age, bone age, height, or sexual maturation stage were observed between the matched groups with and without MetS for each sex (Table 1).

Tanner 1 is pre-pubertal stage. I suggest authors to correct to separate subjects with Tanner stage 1 if there were any

INSERT TABLE 1

Regarding the MetS components, male and female adolescents with MetS exhibited a significant increase in waist circumference, serum triglyceride levels, and systolic and diastolic blood pressure. Serum HDL-c levels were lower and significant in the groups with MetS. There were statistically significantly lower concentrations in the three bone biomarkers in female adolescents with excess weight and MetS compared to the matched group. In male adolescents, the mean concentrations of BAP, OC, and S-CTx were also lower in those with MetS, but a significant difference was only observed for BAP and S-CTx (Table 2).

INSERT TABLE 2

Pearson's correlation coefficients revealed a negative and significant correlation between BMD at all sites and the three bone biomarkers in female adolescents (Table 3). No significant correlation was observed between BMD and the bone biomarkers in male adolescents (Table 4).

elaborate?

elaborate? some contradictory results with multiple correlations.

INSERT TABLE 3 AND 4 NEAR HERE

When the same analysis was performed separately for each MetS component among female adolescents with MetS, the increase in waist circumference was negatively correlated with S-CTx, the increase in blood pressure was negatively correlated with BAP and OC, and the presence of hypertriglyceridemia was positively correlated with BAP and S-CTx. In male adolescents with MetS, the correlation was negative and significant only between increased triglycerides and OC. In adolescents without MetS, a positive and significant correlation was only observed between systolic blood pressure and S-CTx (Table 5).

4. Discussion

Much of the beginning of the discussion is spent on age and puberty affects on bone mass accrual. However, the study's focus is a cross-sectional assessment of MetS on BMD and bone remodeling of age matched groups. Though the groups are heterogeneous incorporating wide age range (10-16 years) and all pubertal stages, there were no clear analysis of affects of age and puberty on the aims of the study in the current study. The first part of the discussion should be revised and shortened to tie in with the findings and the focus of the study.
Also, discussion is focused mainly around bone remodeling markers. There is no discussion of the other findings such as BMD, opposite findings in correlation analyses (Table 3-5), which need to be explained and elaborated on. I suggest major revisions to the discussion session.

Comparis[illegible] were similar in t[illegible] difference in the concentration of the bone biomarkers between the two groups. In this respect, adolescents with MetS exhibited significantly lower concentrations of bone biomarkers (BAP, OC, S-CTx) than those without MetS, except for OC in male adolescents. In addition, BMD in females was negatively correlated with all bone biomarkers in adolescents with MetS.

Bone mass is acquired gradually during childhood. Acceleration in height and weight growth velocities is observed at the beginning of puberty, the so-called pubertal growth spurt, which is also reflected in bone mass acquisition [30]. According to the National Osteoporosis Foundation, the age group in which peak velocity of bone acquisition is achieved is approximately 12.5 ± 0.90 years for female adolescents and 14.1 ± 0.95 years for male adolescents [30]. Following this international

In boys, growth spurt is TS III-IV mid to later puberty. Similarly, bone mass accrual happens later in boys.

which international recommendation?

recommendation, the adolescents in our study are probably at the opportune age for maximum peak velocity of bone acquisition, especially the female adolescents.

Researchers have highlighted the importance of understanding the evolution of BMD and alterations in the levels of bone biomarkers during adolescence [31,32]. Fortes et al. [32], evaluating BMD and its relationship with bone markers in a sample of 101 eutrophic female adolescents of 9 to 20 incomplete years, observed an expressive increase in BMD according to the progression of chronological and bone age. With respect to indicators of bone metabolism, higher mean biomarker levels (BAP=110.12 U/L, OC=26.49ng/mL, S-CTx=1.58 ng/mL) were found when the adolescents were in stage 3 of breast development and in early adolescence (11 to 12 years). The levels of bone biomarkers were lower at the end of adolescence (BAP=31.70 U/L, OC=4.98ng/mL, S-CTx=0.44 ng/mL). Similar results were recently published with the follow-up of 2,416 children and adolescents, from 3 months to 17 years old, analyzing the bone markers Osteocalcin, PINP, and CTX-I. In total, 317 of the participants were obese and the remainder eutrophic [33]. During puberty, the authors demonstrated that the highest levels of bone markers occurred from 10 to 11 years (girls, Tanner 3) and 13 years (boys, Tanner 3–4). These results are very close to those presented by Fortes et al. [32] both in terms of age and pubertal stage.

In male adolescents, Silva et al. [31] studied a sample of 87 eutrophic adolescents aged 10 to 18 years and concluded that serum biomarker levels differed significantly among age groups. The highest concentration of the biomarkers was observed between 13 and 15 years of age (BAP = 155.50U/L, OC = 41.63 ng/mL, S-CTx = 2.09 ng/mL) and the lowest concentration between 16 and 18 years (BAP= 79.80U/L, OC = 27.80 ng/mL, S-CTx = 1.60 ng/mL). Within this context, proportionally higher levels of bone

biomarkers would be expected in the adolescents in this present study, since the mean CA of girls with or without MetS was around 12 years old and BA, around 13 years old, while in boys both CA and BA were around 13 years old. The concentrations obtained among adolescents without MetS in the present study were close to those observed among the eutrophic individuals analyzed in the studies cited [31,32]. However, significant reductions in both biomarkers of bone formation and resorption were observed in the group of adolescents with MetS compared to the matched group without MetS.

Taken together, the results of the present study complement and confirm the findings reported in the literature, showing a significant reduction in BMD at different sites analyzed in adolescents with MetS [5-6].

seems out of place. the discussion is on bone resorption markers.

Studies in adolescents evaluating the impact of MetS on biomarkers of bone formation and resorption are sparse. In recent years, concern has been directed towards the biomarker OC. In the adult population, researchers found that the accumulation of visceral fat, lipid profile alterations, and altered blood pressure, the main components involved in MetS, were correlated with low serum levels of OC [34]. With respect to the relationship between MetS and OC, Tan et al. [35] evaluated 2,344 Chinese men between 20 and 69 years of age and found a negative association between OC levels and the presence of MetS. These results agree with our findings, however, in our results the reductions in OC were significant and negative only in female adolescents with MetS. Thus, analyzing the concentrations of bone biomarkers and the evolution of BMDs according to the sexes, there was a predominance of significant differences for the female sex, with the presence and absence of MetS, which may be related to the nutritional evaluation and biochemical alterations observed between them. In the group of those without the presence of Metabolic Syndrome, 42% of the patients presented no

biochemical alterations (HDL-c, triglycerides, and fasting glucose) and weight (kg) and BMI (kg/m²) (46%) between ≥85th percentile and <95th percentile when compared to those with the presence of MetS. On the other hand, in the group of adolescent males, it was observed, even among those without MetS, that 83% presented biochemical alterations (HDL-c, triglycerides, and fasting glucose) and weight (kg) and BMI (kg/m^2) higher or equal to the 95th percentile were observed in 55.5% of them. This finding possibly influenced the lack of detection of significant differences for the set of biomarkers among male adolescents, when the MetS and absence of MetS groups were compared since, although they were not classified with presence of MetS, they were much more compromised metabolically and nutritionally than the female adolescents.

So, do the authors speculate that they would otherwise have expected to see a difference in males?

The literature states that Osteocalcin, besides being a bone-derived horm[illegible] also associated with energy metabolism. This association is negative (inverse) against hyperglycemia, insulin resistance, hypertriglyceridemia, HDL-c reduction, etc. [35], confirming the connection between bone and energy metabolism [36]. In addition, BAP is also associated with MetS [37], with a strong association with hyperinsulinemia and insulin resistance.

Is it inverse relationship?

Considering the high prevalence of osteoporosis, obesity, and MetS in adults, Laurent et al. [38] evaluated the relationship of MetS with bone turnover in 3,129 men between 40 and 79 years of age. MetS was present in 975 men (31.2%). The men with MetS demonstrated significantly lower levels of all bone biomarkers and higher BMD at the hip, femoral neck, and lumbar spine. When the MetS components were analyzed individually, only hypertriglyceridemia and hyperglycemia were inversely associated with N-terminal propeptide of type I procollagen (PINP) and S-CTx. Osteocalcin was independently and inversely associated only with hyperglycemia. Men with three, four,

or five MetS criteria had lower bone turnover markers. On the other hand, men with one or no criteria presented higher bone turnover markers, indicating the absence of a limit of three criteria above which these markers would be altered. The authors concluded that MetS is associated with lower bone renewal in adult men.

Complementing the small number of publications involving humans, Wong et al. [39] conducted an experimental study to evaluate the effects of MetS induced by a high-carbohydrate high-fat (HCHF) diet on BMD, histomorphometry, and bone markers (OC and S-CTx) in male rats. Twelve Wistar rats (12 weeks old) were randomized into two groups. The normal group received standard chow and the other group received the HCHF diet to induce MetS. There were no significant differences in BMD between normal and HCHF rats ($p>0.05$). The animals fed the HCHF diet had a lower osteoblast surface, higher eroded surface, and lower trabecular bone volume. A significant increase in serum S-CTx levels was observed but no difference in serum OC levels when compared to normal rats. The HCHF diet-induced MetS caused an imbalance in bone remodeling, which led to deterioration of the trabecular bone structure [39].

which components of MetS did the rats have? How do authors explain the difference in human findings with lower resorption markers?

In agreement with the results of the cited studies, in a population of adolescents, it can be stated that MetS alters bone remodeling [3-5] but not all MetS components show similar associations.

Evidence suggests that MetS favors the differentiation of adipocytes and suppresses the differentiation of osteoblasts, reducing bone formation activity. Furthermore, chronic inflammation induced by MetS can increase the formation of osteoclasts and consequently their bone resorption activity [40,41]. However, in the present study there was a reduction in the concentrations of all bone markers analyzed in

both sexes among adolescents with MetS, suggesting an aggression ? to bone formation and resorption, possibly indicating a reduction in remodeling.

With respect to MetS and bone remodeling, studies have reported a chronic inflammatory response caused by inflammatory cytokines (IL-1α, IL-1β, IL-6, and TNF-α) produced by visceral adipose tissue in obese individuals, which was associated with the process of osteoclastogenesis when bone resorption would be higher. This chronic inflammatory response contributes to the reduction in bone mass growth during puberty in obese adolescents [42]. In addition, the presence of insulin resistance, proposed as the essential diagnostic condition of MetS, together with the increase in central adiposity, results in a reduction in adiponectin [43]. Osteoblasts possess receptors for adiponectin, which stimulate the proliferation, differentiation, and mineralization capacity of these cells and inhibit the activity of osteoclasts, consequently reducing bone resorption [44].

Based on animal models of leptin deficiency or deficient leptin signaling, some researchers conclude that this adipokine is a potent inhibitor of bone metabolism [45]. High concentrations of leptin are known to suppress bone formation and stimulate bone resorption. The two leptin-related mechanisms are mediated by RANKL, one related to the action of leptin on hypothalamic receptors, increasing the expression [illegible] consequently bone resorption, and the other through cocaine- and ampheta[illegible] transcription, which results in the inhibition of RANKL [46]. A recent [illegible] negative associations between leptin and OC, suggesting that the inflamm[illegible] negatively influence bone turnover markers in obese adolescents [47].

The authors didn't analyze the effects of inflammation in subjects with MetS on bone remodeling. Thus, I suggest the authors state the limitations of this study not having inflammatory markers evaluation and the presented paragraphs are speculations of the possible mechanisms on bone remodeling. These can be shortened as it has not been tested in the current study.

There is a limited number of prospective trials, either cohort or clinical, and descriptive studies investigating the evolution of bone biomarkers in adolescents with excess weight and with a diagnosis of MetS. To our knowledge, this is the first study

demonstrating the impact of MetS on bone remodeling in adolescents. Thus, in view of the scarce results in the literature, the combined analysis of biochemical markers of bone formation and resorption in a matched sample selected using rigorous inclusion criteria can be considered a strength of the present study. Regarding the cross-sectional design of the study, this prevents the provision of information about the temporal sequence of events and does not permit identification of causal relationships. Previous studies demonstrate an involvement in bone mass between those who are obese and severe/extremely obese and those with the [illegible]

this statement is not clear. suggest authors clarify what involvement in bone mass do they mean.

bone turnover markers quickly reveal, in real time, an accompaniment that is only proven by densitometry over a longer period of time, even if it is accepted as the gold standard in bone mass evaluation. It is clear that the recommendation to perform both procedures in adolescents remains desirable, recommended, and complementary.

unclear. needs revision. what does DXA prove? It is known DXA has many limitations especially in pediatrics.

The current study has some limitations, among which we did not evaluate the pro and anti-inflammatory cytokines of adolescents, which are possibly involved in MetS. In addition, serum levels of 25-hydroxyvitamin D (s25 (OH) D) were not measured, and a deficiency/insufficiency in this biomarker has been described in association with the presence of MetS components in children [49], adolescents, and adults [50]. However, this study contributed by suggesting possible designs for future studies, especially prospective studies, with the inclusion of a larger number of subjects and the possibility of generalizing the results.

In summary, the inclusion and interpretation of this set of bone biomarkers and of others to be incorporated in the future, will complement and broaden knowledge about bone metabolism in adolescents with and without MetS.

5. Conclusion

Our results suggest that adolescents with excess weight and the presence of MetS presented lower concentrations of biomarkers of bone formation and resorption. The presence of MetS could be detrimental to the development of bone mass in adolescents. The reduction in BMD and in the markers responsible for bone formation during this phase of life may compromise the acquisition of peak bone mass, which could increase the current fracture risk and result in bone microarchitecture derangement and bone fragility in the years to come.

Acknowledgements: Supported by FAPESP (Fundação de Amparo à Pesquisa do Estado de São Paulo) – Grants (07/07731-0, 2011/05991-0 and 2015/04040-2) and the Pro-Rector for Research of UNESP.

References

1. Alhazidou E, Pergialiotis V, Chrelias C, Hatziagelaki E, Papantoniou N, Trakakis E. The impact of the metabolic syndrome on bone mass density: a prospective case control study. Horm Mol Biol Clin Investig. 2017; 33:1-6.

2. Al-Hamad D, Raman V. Metabolic syndrome in children and adolescents. Transl Pediatr. 2017; 6:397-407.

3. Silva VND, Fiorelli LNM, Silva CC, Kurokawa CS, Goldberg TBL. Do Metabolic syndrome and its components have an impact on bone mineral density in adolescents? Nutrition Metabolism (Lond). 2017; 14:1-7.

4. Pollock NK, Bernard PJ, Gutin B, Davis CL, Zhu H, Dong YMD. Adolescent obesity, bone mass, and cardiometabolic risk factors. J Pediatr. 2011; 158:727-734.

5. Nóbrega da Silva V, Goldberg TBL, Mosca LN, Rizzo ACB, Teixeira AS, Corrente JE. Metabolic syndrome reduces bone mineral density in overweight adolescents. Bone. 2014; 66:1-7.

6. Hethering-Rauth M, Bea JW, Blew RM, Funk JL, Lee VR, Roe DJ, Sardinha LB, Going SB. Relationship of cardiometabolic risk biomarkers with DXA and pQCT bone health outcomes in young girls. Bone. 2019; 120:452-458.

7. Lee K. Sex-Specific Relationships between Insulin Resistance and Bone Mineral Content in Korean Adolescents. J Bone Miner Metab. 2013; 31: 177–182.

8. Lawlor DA, Sattar N, Sayers A, Tobias JH. The association of fasting insulin, glucose, and lipids with bone mass in adolescents: findings from a cross sectional study. J Clin Endocrinol Metab. 2012; 97: 2068–2076.

9. Naylor K, Eastell R. Bone turnover markers: use in osteoporosis. Nat Rev Rheumatol 2012.8:379-89.

10. Seibel MJ. Bone turnover in nutrition-related disorders. Wien Med Wochenschr. 2007; 157: 582-588.

11. Kim GS, Jekal Y, Kim HS, Im JA, Park JY, Chu V. Reduced serum total osteocalcin is associated with central obesity in korean children. Obes Res Clin Pract. 2014; 8:201-208.

12. Garanty-Bogacka B, Syrenicz M, Rac M, Krupa B, Czaja-bulsa G, Walczak M, Sowinska-Przepier E, Syrenicz. Association between serum osteocalcin, adiposity and metabolic risk in obese children and adolescents. Endokrynol Pol. 2013; 64:346-352.

13. Kuczmarski RJ, Ogden CL, Guo SS, Grummer-Strawn LM, Flegal KM, Mei Z, Wei R, Curtin LR, Roche AF, Jhonson CL 2000. CDC Growth Charts for the United States: Methods and Development. Vital Health Stat. 2002; 11 246: 1-190.

14. Institute of medicine (IOM), Dietary reference intakes for energy, carbohydrate, fiber, fat, fatty acids, cholesterol, protein, and amino acids (macronutrients). Washington DC: National Academies Press, 2005.

15. Rapuri PB, Gallagher JC, Kinyamu HK, Ryschon KL. Caffeine intake increases the rate of bone loss in elderly women and interacts with vitamin d receptor genotypes. Am J Clin Nutr. 2001; 74:694-700.

16. Fitzpatrick L, Heaney RP. Got soda? J Bone Miner Res. 2003; 18:1570–1572.

17. Mcgartland C, Robson PJ, Murray L. Carbonated soft drink consumption and bone mineral density in adolescence: the Northern Ireland young hearts project. J Bone Mineral Res. 2003; 18:1563–1569.

18. Kuczmarski RJ, Ogden CL, Guo SS, Grummer-Strawn LM, Flegal KM, Mei Z, Wei R, Curtin LR, Roche AF, Jhonson CL 2000. CDC Growth Charts for the United States: Methods and Development. Vital Health Stat. 2002; 11 246: 1-190.

19. Barlow SE, Expert Committee. Expert committee recommendations regarding the prevention, assessment, and treatment of child and adolescent overweight and obesity: summary report. Pediatrics. 2007; 120: Suppl 4:S164-92.

20. Xanthakos SA, Inge TH. Extreme pediatric obesity: weighing the health dangers. J Pediatr. 2007; 150:3-5.

21. National Health and Nutrition Examination Survey (NHANES). Anthropometry procedures manual. Examination Survey, 2017.

22. Sinaiko AR, Steinberger J, Moran A, Hong CP, Prineas RJ, Jacobs DR Jr. Influence of insulin resistance and body mass index at age 13 on systolic blood pressure, triglycerides, and high-density lipoprotein cholesterol at age 19. Hypertension. 2006; 48:730-736.

23. Marshall WA, Tanner JM. Variations in pattern of pubertal changes in girls. Arch Dis Child. 1969; 44:291-303.

24. Marshall WA, Tanner JM. Variations in the pattern of pubertal changes in boys. Arch Dis Child. 1970; 45:13-23.

25. Finkelstein JW. The endocrinology of the adolescent. Symposium of adolescent medicine. Pediatrics Clinic of North America.1980; 27:53-69.

26. Greulich W, Pyle S. Radiographic atlas of skeletal development of hand and wrist. 2nd ed. Stanford: Stanford University Press, 1959.

27. Zimmet P, Alberti KG, Kaufman F, Tajima N, Silink M, Arslanian S, Wong G, Bennett P, Shaw P, Caprio S, IDF CONSENSUS GROUP (2007). The metabolic syndrome in children and adolescents–an IDF consensus report. Pediatr Diabetes 8:299-306.

28. Hill KM, Braun MM, Egan KA, Martin BR, McCabe LD, Peacock M, et al. Obesity augments calcium-induced increases in skeletal calcium retention in adolescents. J Clin Endocrinol Metab. 2011; 96:2171-7.

29. Gordon CM, Leonard MB, Zemel BS. 2013 Pediatric Position Development Conference: executive summary and reflections. J Clin Densitom. 2014; 17:219-224.

30. Weaver CM, Gordon CM, Janz KF, Kalkwarf HJ, Lappe JM, Lewis R, Okarma M, Wallace TC, Zemel BS. The national osteoporosis foundation's position statement on peak bone mass development and lifestyle factors: a systematic review and implementation recommendations. Osteoporos Int. 2016; 27:1281-1386.

31. Silva CC, Kurokawa CS, Nga HS, Moretto MR, Dalmas JC, Goldberg TBL. Bone metabolism biomarkers, body weight, and bone age in healthy Brazilian male adolescents. J Pediatr Endocrinol Metab. 2012; 25: 479-484.

32. Fortes CMT, Goldberg TBL, Kurokawa CS, Silva CC, Moretto MR, Biason TP, Teixeira AS, Nunes HRC. Relação entre as idades cronológica e óssea e o estágio puberal das mamas com os biomarcadores ósseos e a densidade mineral óssea em adolescentes. J Pediatr (Rio J). 2014; 90:624-631.

33. Geserick M, Vogel M, Eckelt F, Schlingmann M, Hiemisch A, Baber R, Thiery J, Korner A, Kiess W, Kratzsch J. Children and adolescents with obesity have reduced serum bone turnover markers and 25-hydroxyvitamin D but increased parathyroid hormone concentrations - Results derived from new pediatric reference ranges. Bone. 2020;132:115-124.

34. Bae SJ, Choe JW, Chung YE, Kim BJ, Lee SH, Kim HY, Koh JM, Kim HK, Kim GS. The association between serum osteocalcin levels and metabolic syndrome in Koreans. Osteoporos Int. 2011; 22:2837- 2846.

35. Tan A, Gao Y, Yang X, Zhang H, Qin X, Mo L, Peng T, Ning X, Mo Z. Low serum osteocalcin level is a potential marker for metabolic syndrome: results from a Chinese male population survey. Metabolism. 2011; 60:1186-1192.

36. Fernandes TAP, Gonçalves LML, Brito JAA. Relationships between Bone Turnover and Energy Metabolism. Journal of Diabetes Research.2017; 27: 1.

37. Cheung CL, Tan KC, Lam KS, Cheung BM. The relationship between glucose metabolism, metabolic syndrome, and bone-specific alkaline phosphatase: a structural equation modeling approach. J Clin Endocrinol Metab. 2013.9:3856-63.

38. Laurent MR, Cook MJ, Gielen E, Ward KA, Antonio L, Adams JE, Decallone B, Bartfai G, Casanueva FF, Forti G, Giwercman A, Huhtaniemi IT, Kuka K, Lean MEJ, Lee DM, Pendleton N, Punab M, Claessens F, Wu FCW, Vanderschueren D, Pye SR, O'Neill TW, EMAS GROUP. Lower bone turnover and relative bone deficits in men with metabolic syndrome: a matter of insulin sensitivity? The European Male Ageing Study, Osteoporos Int. 2016; 27:3227–3237.

39. Wong SK, Chin KY, Suhaimi FH, Ahmad F, Ima-Nirwana S. Effects of metabolic syndrome on bone mineral density, histomorphometry and remodeling markers in male rats. PLoS One. 2018; 13:e0192416.

40. Beederman M, Lamplot JD, Nan G, Wang J, Liu X, Yin L, Li R, Shui W, Zhang H, Kim SK, Zhang W, Zhang J, Kong Y, Denduluri S, Rogers MR, Pratt A, Haydon RC, Luu HH, Angeles J, Shi LL, He TC. BMP signaling in mesenchymal stem cell differentiation and bone formation. J Biomed Sci Eng. 2013; 6(8A):32-52.

41. Felice JI, Gangoiti MV, Molinuevo MS, Mccarthy AD, Cortizo AM. Effects of a Metabolic Syndrome induced by a fructose-rich diet on bone metabolism in rats. Metabolism. 2014;63: 296-305.

42. Cao JJ. Effects of obesity on bone metabolism. J Orthop Surg Res. 2011; 30:1-7.

43. Rubin DD, Mcmurray RR, Harrell JS, Hackney AC, Hagg AM. Do surrogate markers for adiposity relate to cytokines in adolescents? J investig Med. 2008; 56:786-792.

44. Yamaguchi T, Sugimoto T. Bone metabolism and fracture risk in type 2 diabetes mellitus. Bonekey Rep. 2012; 1:36.

45. Elefteriou F, Ahn JD, Takeda S, Starbuck M, Yang X, Liu X, Kondo H,. Richards WG, Bannon TW, Noda M, Clement K, Vaisse C, Karsenty G. Leptin regulation of bone resorption by the sympathetic nervous system and CART. Nature. 2005; 434:514-20.

46. Foo JP, Polyzos SA, Anastasilakis AD, Chou S, Mantzoros CS. The effect of leptin replacement on parathyroid hormone, RANKL-osteoprotegerin axis, and Wnt inhibitors in young women with hypothalamic amenorrhea. J Clin Endocrinol Metab. 2014; 99: E2252–2258.

47. Campos RMDS, Masquio DCL, Corgosinho FC, Carvalho-Ferreira JP, Molin Netto BD, Clemente APG, Tock L, Tufik S, Mello MT, Dâmaso AR. Relationship between adiponectin and leptin on osteocalcin in obese adolescents during weight loss therapy. Arch Endocrinol Metab. 2018; 62:275-284.

48. Mosca LN, Goldberg TBL, Silva VN, Kurokawa CS, Rizzo ACB, da Silva CC, Teixeira AS, Corrente JE. The impact of excess body fat on bone remodeling in adolescentes. Osteoporos Int. 2017. 28:1053–1062.

49.Nwosu BU, Maranda L, Cullen K, Ciccarelli C, Lee MM. Vitamin D status is associated with early markers of cardiovascular disease in prepubertal children. J Pediatr Endocrinol Metab. 2013;26(11-12):1067-75. doi: 10.1515/jpem-2013-0086.

50. Fu J, Han L, Zhao Y, Li G, Zhu Y, Li Y, Li M, Gao S, Willi SM. Vitamin D levels are associated with metabolic syndrome in adolescents and young adults: The BCAMS study. Clin Nutr. 2019.38:2161-2167.

Funding: FAPESP (Fundação de Amparo à Pesquisa do Estado de São Paulo) – Grants (07/07731-0, 2011/05991-0 and 2015/04040-2).

Availability of data and materials: Data that support the findings of this study are available from the corresponding author upon reasonable request.

Ethics approval and consent to participate: The study was approved by the Ethics Committee of the Botucatu Medical School (FMB-UNESP) (Approval No. 1.399.709 /2016).

Consent for publication: Prior to data collection, the purpose of the study was explained to the participants and their informed consent was recorded.

Authors' contributions: All authors have read and agreed to the publication of this paper. V.N.S. and T.B.L.G. wrote the first draft and, together with L.N.M.F., C.S.K., C.C.S., and A.C.B.R. contributed equally to this study by providing opinions and constructive criticism regarding the content. José Eduardo Corrente was the statistical consultant.

Competing interests: The authors declare that they have no competing interests.

Table 1. Comparison of adolescents with and without metabolic syndrome according to sex, bone age, tanner stage, anthropometric measures, body mass index, BMD, and transformed BMD of both genders.

**Parameter**		**Female (n=48)**			**Male (n=36)**		
		**MetS (-) (n=24)**	**MetS (+) (n=24)**		**MetS (-) (n=18)**	**MetS (+) (n=18)**	
		**Mean ± SD**		***p* value[a]**	**Mean ± SD**		***p* value[a]**
Age (years) [a]		11.78±1.29	12.58±1.95	0.102	13.03±1.30	13.42±1.23	0.393
Bone age (years)[a]		13.09±1.75	13.40±2.07	0.582	13.73±0.92	13.86±1.88	0.813
Tanner stage [n (%)][b]		***Breast development***			***Genital***		
Early puberty	1<br>2	1 (4)	2 (9)		2 (14)	2 (14)	
Mid-puberty	3	8 (35)	6 (29)	0.690	5 (33)	4 (29)	0.994
Late puberty	4<br>5	14 (61)	13 (62)		8 (53)	8 (57)	
Weight (kg)[a]		58.85±9.46	73.58±13.92	<0.000*	71.32±14.58	81.10±13.44	0.044*
Height (m)[a]		1.54±0.07	1.57±0.07	0.167	1.63±0.09	1.64±0.09	0.728
BMI (kg/m^2)[a]		24.61±2.64	29.32±4.16	0.038*	26.44±4.22	29.61±4.21	0.030*
Lumbar spine BMD (g/cm^2)		0.82±0.15	0.90±0.19	0.123	0.77±0.12	0.85±0.13	0.052
Femoral BMD (g/cm^2)		0.89±0.12	0.95±0.14	0.124	0.95±0.16	1.00±0.14	0.323
Total body BMD (g/cm^2)		0.90±0.09	0.94±0.10	0.162	0.92±0.08	0.94±0.09	0.609
Subtotal body BMD (g/cm^2)		0.81±0.08	0.85±0.09	0.150	0.84±0.09	0.86±0.09	0.560
		BMD transformed for body weight (g/cm^2/kg body weight).					

TS 1 is prepubertal

I suggest authors include BMD z-scores

Lumbar spine BMD	0.0141±0.0021	0.012±0.002	0.005*	0.011±0.002	0.010±0.001	0.416
Femoral BMD	0.0154±0.0024	0.013±0.001	0.001*	0.013±0.002	0.012±0.002	0.153
Total body BMD	0.0156±0.0023	0.013±0.001	<0.000*	0.013±0.002	0.011±0.002	0.042*
Subtotal body BMD	0.0141±0.0020	0.011±0.001	<0.000*	0.012±0.001	0.010±0.002	0.050*

MetS (−): without metabolic syndrome; MetS (+): with metabolic syndrome.
BMI, Body Mass Index.
[a] Student t-test. [b] Chi-square test.

Table 2. Comparison of metabolic syndrome components and bone biomarkers between adolescents with and without MetS according to sex.

**Parameter**	**Female (n=48)**			**Male (n=36)**		
	**MetS (-) (n=24)**	**MetS (+) (n=24)**		**MetS (-) (n=18)**	**MetS (+) (n=18)**	
	Mean ± SD		*p* value[a]	Mean ± SD		*p* value[a]
MetS component						
WC (cm)	83.70±7.86	94.94±7.48	<.000	86.80±7.35	92.68±9.56	0.051
HDL-c (mg/dL)	46.60±10.83	39.62±9.41	0.022	43.77±7.89	36.27±3.21	0.008
SBP (mmHg)	104.70±11.06	117.30±13.64	0.001	116.60±9.84	128.40±13.70	0.005
DBP (mmHg)	63.77±6.79	73.89±8.09	<.000	69.58±5.86	80.79±9.00	0.000
Triglycerides (mg/dL)	88.82±19.80	149.90±52.03	<.000	99.38±41.74	172.60±72.35	0.000
Fasting glucose (mg/dL)	82.78±6.19	86.75±8.96	0.085	87.83±6.90	87.22±7.58	0.891
Bone biomarker						
BAP (U/L)	178.10±46.26	121.50±65.68	0.001	244.10 ±56.96	179.70±77.46	0.008
OC (ng/mL)	39.08±16.10	21.02±12.15	<.000	40.07±12.52	31.03±15.59	0.066
S-CTx (ng/mL)	1.56±0.52	1.22±0.46	0.027	1.93±0.33	1.60±0.53	0.034

(−): MetS components without alteration; (+): MetS components with alteration.
WC, waist circumference; HDL-c, HDL-cholesterol; SBP, systolic blood pressure; DBP, diastolic blood pressure; BAP, bone alkaline phosphatase; OC, osteocalcin; S-CTx, carboxy-terminal telopeptide. [a] Student t-test.

Table 3. Pearson's correlation between bone biomarkers and bone mineral density measured at four sites in female adolescents with and without metabolic syndrome.

Parameter		MetS (-)			MetS (+)		
		**BAP (U/L)**	**OC (ng/mL)**	**S-CTx (ng/mL)**	**BAP (U/L)**	**OC (ng/mL)**	**S-CTx (ng/mL)**
Lumbar Spine BMD (g/cm²)	*r*	-0.04088	-0.09311	0.04937	-0.71742	-0.53505	-0.51012
	p	0.873	0.665	0.818	<.000	0.007	0.018
Femoral BMD (g/cm²)	*r*	-0.03186	-0.07059	0.09377	-0.52271	-0.68411	-0.54997
	p	0.882	0.743	0.663	0.008	0.000	0.009
Total body BMD (g/cm²)	*r*	-0.04088	-0.16939	0.10861	-0.55568	-0.81760	-0.53838
	p	0.8496	0.428	0.613	0.005	<.000	0.011
Subtotal Body BMD (g/cm²)	*r*	-0.04533	-0.18300	0.12481	-0.79215	-0.47089	-0.54338
	p	0.833	0.392	0.561	<.000	0.023	0.010

MetS (−): without metabolic syndrome; MetS (+): with metabolic syndrome.
BMD, bone mineral density; BAP, bone alkaline phosphatase; OC, osteocalcin; S-CTx, carboxy-terminal telopeptide.
Pearson correlation test.

Table 4. Pearson's correlation between bone biomarkers and bone mineral density measured at four sites in male adolescents with and without metabolic syndrome.

Parameter		MetS (-)			MetS (+)		
		**BAP (U/L)**	**OC (ng/mL)**	**S-CTx (ng/mL)**	**BAP (U/L)**	**OC (ng/mL)**	**S-CTx (ng/mL)**
Lumbar spine BMD (g/cm^2)	r	-0.42803	0.02406	-0.07669	-0.09165	0.30822	0.01061
	p	0.076	0.924	0.762	0.726	0.228	0.970
Femoral BMD (g/cm^2)	r	-0.20502	0.2714	0.22064	-0.03393	0.30356	-0.03041
	p	0.414	0.274	0.379	0.897	0.236	0.914
Total body BMD (g/cm^2)	r	-0.31354	0.22305	-0.08190	-0.07856	0.22114	-0.14209
	p	0.205	0.373	0.746	0.764	0.393	0.613
Subtotal body BMD (g/cm^2)	r	-0.30173	0.22308	-0.08440	-0.05501	0.15457	-0.08828
	p	0.223	0.373	0.739	0.8339	0.5536	0.7544

MetS (−): without metabolic syndrome; MetS (+): with metabolic syndrome.
BMD, bone mineral density; BAP, bone alkaline phosphatase; OC, osteocalcin; S-CTx, carboxy-terminal telopeptide.
Pearson correlation test.

Table 5. Pearson's correlation between bone biomarkers and metabolic syndrome components in adolescents of both sexes with and without the syndrome.

MetS component		**MetS (-)**			**MetS (+)**		
		**BAP (U/L)**	**OC (ng/mL)**	**S-CTx (ng/mL)**	**BAP (U/L)**	**OC (ng/mL)**	**S-CTx (ng/mL)**
Female							
WC (cm)	r	-0.29836	-0.07739	-0.24094	-0.32473	-0.24808	-0.46752
	p	0.177	0.732	0.280	0.121	0.242	**0.032***
HDL-c (mg/dL)	r	0.13497	-0.0581	-0.11359	-0.01574	-0.28168	-0.00502
	p	0.539	0.792	0.605	0.941	0.182	0.982
SBP (mmHg)	r	-0.22598	-0.0187	-0.16776	-0.50237	-0.46993	-0.17346
	p	0.311	0.934	0.455	**0.014***	**0.023***	0.464
DBP (mmHg)	r	-0.21424	0.00212	-0.20241	-0.35271	-0.4547	-0.02847
	p	0.338	0.992	0.366	0.098	**0.029***	0.905
Triglycerides (mg/dL)	r	-0.40542	-0.12222	-0.19898	0.46519	0.20343	0.51017
	p	0.055	0.578	0.362	**0.022***	0.340	**0.018***
Fasting glucose (mg/dL)	r	-0.00579	-0.22448	-0.23428	-0.09644	-0.0954	-0.17617
	p	0.979	0.303	0.281	0.654	0.657	0.444
Male							
WC(cm)	r	0.45455	0.19324	0.11532	0.14272	0.39229	0.19134
	p	0.058	0.442	0.648	0.598	0.132	0.512
HDL-c (mg/dL)	r	0.00244	0.29945	0.05388	-0.33351	-0.18099	-0.07285
	p	0.992	0.227	0.831	0.190	0.487	0.796
SBP (mmHg)	r	0.02191	0.30439	0.34550	0.15801	0.20069	0.37223
	p	0.931	0.219	0.160	0.558	0.456	0.190
DBP (mmHg)	r	0.30301	0.05582	0.53446	0.24027	0.14589	0.26202
	p	0.221	0.825	**0.022***	0.370	0.589	0.365
Triglycerides (mg/dL)	r	-0.20649	-0.11482	0.22251	-0.22418	-0.48347	-0.15625
	p	0.411	0.650	0.374	0.387	**0.049***	0.578
Fasting glucose (mg/dL)	r	-0.19083	-0.1420	0.05144	0.17677	0.13868	-0.14624
	p	0.448	0.568	0.839	0.497	0.595	0.603

MetS (−): without metabolic syndrome; MetS (+): with metabolic syndrome.
BAP, bone alkaline phosphatase; OC, osteocalcin; S-CTx, carboxy-terminal telopeptide; WC, waist circumference; HDL-c, [illegible] cholesterol; SBP, systolic blood pressure; DBP, diastolic blood pressure. Pearson correlation test.

Dataset

Click here to access/download
Supporting Information
Dataset of impacto of_.xlsx

Impact of metabolic syndrome and its components on bone remodeling in adolescents

Metabolic syndrome and bone remodeling

Valéria Nóbrega da Silva[a], Tamara Beres Lederer Goldberg[a*], Carla Cristiane Silva[b], Cilmery Suemi Kurokawa[c], Luciana Nunes Mosca Fiorelli[a], Anapaula da Conceição Bisi Rizzo[a], José Eduardo Corrente[d]

[a]Postgraduate Program in Gynecology, Obstetrics, and Mastology, Discipline of Adolescent Medicine, Department of Pediatrics, Botucatu Medical School, UNESP, São Paulo State University, Botucatu, SP, 18618-970, Brazil; E-mail addresses: valerianutry@gmail.com; tamara.goldberg@unesp.br; lunutricionista@yahoo.com.br; anapaula.rizzo@uol.com.br

[b]Department of Physical Education, University of North Paraná, Jacarezinho, Paraná, Brazil; E-mail address: ccsilva@uenp.edu.br

[c]Clinical and Experimental Pediatric Research Center, Department of Pediatrics, Botucatu Medical School, UNESP, São Paulo State University, Botucatu, SP, Brazil; E-mail address: kurokawa@fmb.unesp.br

[d] Department of Statistics, Biosciences, UNESP, São Paulo State University, Botucatu, SP, Brazil; E-mail address: jecorren@ibb.unesp.br

Corresponding author:

Department of Pediatrics, Botucatu Medical School, UNESP, São Paulo State University, Brazil. (Tel.: +55-14-3811-6274/+55-14-3811-6083)

e-mail. tamara.goldberg@unesp.br

Abstract

Osteoporosis and metabolic syndrome (MetS) are diseases that have serious public health consequences, reducing the quality of life of patients and increasing morbidity and mortality, with substantial healthcare expenditures.

Objective

To evaluate the impact of MetS on bone mineral density (BMD) and biochemical markers of bone formation and resorption in adolescents with excess weight.

Method

A descriptive and analytical cross-sectional study that evaluated 271 adolescents of both sexes (10 to 16 years). From the total sample, 42 adolescents with excess weight and the presence of MetS (14%) were selected. A further 42 adolescents with excess weight and without MetS were selected and matched for chronological age, bone age, and pubertal developmental criteria to those with MetS, for each sex. The adolescents were submitted to anthropometry, blood pressure measurement, and biochemical tests, as well as evaluation of BMD and the bone biomarkers osteocalcin (OC), bone alkaline phosphatase (BAP), and carboxy-terminal telopeptide (S-CTx).

Results

The adolescents with excess weight and MetS exhibited a significant decrease in BMD and concentrations of BAP, OC, and S-CTx compared to the matched group, except for OC in boys. A negative and significant correlation was observed between total body BMD and BAP ($r = -0.55568$; $p = 0.005$), OC ($r = -0.81760$; $p = <.000$), and S-CTx ($r = -0.53838$; $p = 0.011$) in girls. ~~Adolescents with three MetS components presented lower BAP and OC levels than those with zero, one, or two components.~~

Conclusion

The presence of MetS is detrimental to the bone mineral density (BMD) and biochemical markers of bone formation and resorption in adolescents with excess weight**.**

Keywords: Adolescents; Bone, Metabolic Syndrome

Introduction

Osteoporosis and metabolic syndrome (MetS) are diseases that have serious public health consequences, reducing the quality of life of patients and increasing morbidity and mortality, with substantial healthcare expenditures [1]. The occurrence of MetS in adolescents is associated with an increased risk of cardiovascular diseases, psychosocial problems, abnormal glucose metabolism, nonalcoholic fatty liver disease, polycystic ovary syndrome, obstructive sleep apnea, mental disorders, orthopedic complications, and disorders of motor development [2]. In addition, to these conditions, although a possible relationship between this syndrome and bone mass has been demonstrated, the number of studies addressing this topic in adolescents is still small [3]. Despite the paucity of evidence, the results of some studies reveal that MetS is negatively correlated with bone mass and that some components of MetS are negatively correlated with BMD in adolescents [4-8].~~ previous studies have non-definitively demonstrated a relationship between the occurrence of MetS and possible impairment in bone mass in young and adult populations [3 - 6].~~

In adolescence, the period of pubertal development is important for the acquisition of bone mineral content. Especially in this phase of life, which is dominated by physical growth, there is an imbalance between bone formation and resorption, and the biochemical formation markers are correlated with the rate of growth [9]. ~~During the process of bone turnover, substances known as biochemical markers of bone metabolism~~

~~are released into the blood. These substances result from the physiologically coupled action between osteoclasts and osteoblasts. However, during this phase of life, bone formation is more intense than resorption.~~Under normal physiological conditions, bone resorption and formation are dependent phenomena and the predominance of one over the other may lead to the gain or loss of bone mass [10].

The relationship of bone mass with the occurrence of MetS in children and adolescents has been a subject of interest to some researchers [3- 6, 11], since impairment in bone acquisition during this period can lead to "suboptimal" peak bone mass and increase the risk of developing osteopenia/osteoporosis and fractures in old age [7]. A recent systematic review performed a critical analysis of studies that focused on the period of adolescence, evaluating the influence of MetS and its components on bone mineral density (BMD) in this age group [3].~~ Although the possible relationship between this syndrome and bone mass has been demonstrated, the number of studies addressing this topic in adolescents is still small [8]. Despite the paucity of evidence, the results of some studies reveal that MetS is negatively correlated with bone mass and that some components of MetS are negatively correlated with BMD in adolescents [3, 5, 6, 9 – 10].~~

In adolescents, the relationship between MetS and markers of bone formation and resorption is not fully known. However, some lines of evidence indicate a negative correlation between serum levels of the bone marker osteocalcin and metabolic risk factors in obese children and adolescents [12]. In view of the results already published [3, 5, 6],~~ it is important to evaluate the behavior of some biomarkers of bone formation (bone alkaline phosphatase – BAP and osteocalcin – OC) and resorption (carboxy-terminal telopeptide – S-CTx) in adolescents with MetS in order to confirm our hypothesis that this syndrome and its components exert a negative impact on bone mass, even in the first~~

~~two decades of life~~. the objective of this study was to evaluate the impact of MetS and each of its components on BMD and on biochemical markers of bone formation and resorption in adolescents.

2. Material and methods

2.1 Study Design and Participants

This was a descriptive and analytical cross-sectional study involving a sample of 271 adolescents with excess weight [5]. For methodological reasons, the final sample consisted of only 84 adolescents because of the need to analyze two matched groups with and without MetS. The groups were matched for chronological age, bone age, and pubertal stage for each sex. Our sample of 84 adolescents did not differ from that of the 271 initially included subjects. The sample of adolescents of both sexes with and without MetS and with excess weight was selected based on the availability of care for new cases at the Adolescent Outpatient Clinic of the University Hospital, Botucatu Medical School (SP, Brazil) on the occasion of their first visit. Data were collected from March 2011 to March 2013. The adolescents were invited to participate in the study at the time of their visit to the outpatient clinic and a written informed consent form was signed by the adolescents and by their parents or legal guardians. The study was approved by the Research Ethics Committee of the Botucatu Medical School (Approval Nº. 1.399.709 /2016).

The inclusion criteria were as follows: 10 to 16 years of age, overweight (BMI ≥85th percentile for sex and age), and between the 5th and 95th age- and sex-appropriate height percentiles [13]. Adolescents were excluded from this study for the following reasons: weight exceeding 100 kg (densitometry was not feasible for these potential

subjects), a history of prematurity, a history of fracture in the previous two years, prolonged therapy with corticosteroids, use of calcium and/or iron supplements in the 12 months preceding this research, presence of chronic diseases, use of drugs that negatively affect bone metabolism such as medroxyprogesterone acetate, anticonvulsants, or antacids containing aluminum, consumption of an exclusive high-fiber diet containing above age-appropriate recommendations (>26 g/day for female adolescents or >31 g/day for male adolescents (9–13 years) or >38 g/day for male and female adolescents 14–16 years) [14], a vegan or vegetarian diet, caffeine intake >300 mg/day (>3 cups of coffee per day) [15] consumption of more than 500 mL/day of soft drinks [16, 17], and lack of daily consumption of dairy products. Furthermore, the adolescents did not smoke or drink alcohol, were not engaged in any extracurricular sporting activities, and participated in physical education classes in their schools for no more than 2 h/week. Other exclusion criteria were the presence of metabolic, endocrine, or genetic diseases (as verified by a history of current disorders, general and specific physical examinations, laboratory or radiological procedures), any changes in menstrual cycle (oligo/amenorrhea, clinical or biochemical hyperandrogenism) that could suggest the presence of polycystic ovary syndrome, use of combined oral contraceptives, and pregnancy in girls. These exclusion criteria were established in order to prevent other events or situations described in the literature from interfering with the increase in bone mass in these adolescents. If adolescents presented any mental alterations or used medication to treat mental disorders they were not included in the study.

Adolescents of both sexes were excluded if they failed to attend all scheduled appointments for anthropometric measurements or blood sampling or if they did not follow the established sample collection procedures; e.g., if they did not fast for at least

10 h prior to collection. Our sample of 84 adolescents did not differ from that of the 271 initially included subjects. For methodological reasons, the final sample consisted of only 84 adolescents because of the need to analyze two matched groups with and without MetS. The groups were matched for chronological age, bone age, and pubertal stage for each sex.

2.2 Anthropometric Assessment and Blood Pressure Measurement

Anthropometric measures of weight (kg) and height (m) were obtained as recommended by the National Health and Nutrition Examination Survey [17], from which the body mass index was calculated (BMI, kg/m^2), utilized to assess nutrition status. The BMI percentiles were obtained with Epi Info 3.5.1 Software, which uses sex and age growth curves as references [18]. Adolescents with a BMI between the 85th and 95th percentile were classified as overweight, between the 95th and 99th percentile as obese [19], and above the 99th percentile as extremely obese [20]. Waist circumference (cm) was obtained at the midpoint between the last rib and the iliac crest at the end of a regular expiration [21]. Blood pressure was measured with the adolescent in a sitting position after a 5-min rest. The mean of three measurements with 3-min intervals was used. Measurements were performed on the right arm by auscultation, using a calibrated mercury sphygmomanometer and a cuff appropriate for the arm circumference [22].

2.3 Evaluation of Sexual Maturation and Skeletal Maturation Stages

Sexual maturity was evaluated by an experienced physician through visual breast evaluation (females) or genital observation (males) and the results reported using the

Tanner scale [23, 24], and grouped according to Finkelstein et al. [25] into early puberty, mid-puberty, and late puberty.

To evaluate skeletal maturation, bone age was obtained by the GP method [26] in which the hand and wrist radiographs were compared to the atlas. This examination was performed under the supervision and guidance of a specialized professional who was unaware of the anthropometric and densitometric characteristics of the adolescents (blind evaluator).

2.4 Biochemical Evaluation

Blood samples were obtained in the morning after a 10-h fast, between 7:00 am and 9:00 am, and all storage precautions were taken. The samples were collected by well-trained laboratory technicians and evaluated at the Central Laboratory. Dry chemistry was used to assess HDL-c, triglycerides, and fasting glucose in a Vitros 950 analyzer (Johnson and Johnson) according to the manufacturer's instructions. Values are expressed as mg/dL. MetS was defined according to the criteria proposed by the IDF [27]. The adolescent aged 10 to 16 years were ~~subject was~~ classified as having MetS if they presented central obesity, defined by a large waist circumference (>90th percentile of WC by sex and age), and at least two of the four criteria: (TG ≥150 mg/dL, HDL <40 mg/dL, SBP ≥130 mmHg or DBP ≥85 mmHg, and fasting blood glucose ≥100 mg/dL or a type 2 diabetes diagnosis).

~~**INSERT TABLE 1**~~

The blood samples were collected by venous puncture and centrifuged for 15 min at 1500*g* for the separation of serum. The serum samples were stored at -70°C until the

time of analysis of the biomarkers (BAP, OC, and S-CTx). BAP and intact OC were measured using an assay from Metra™ Biosystems (San Diego, CA, USA), with intra- and inter-assay coefficients of variation of 8 and 7.6%, respectively. S-CTx was quantified by an electrochemiluminescence immunoassay (ECLIA) using the Elecsys beta-Cross Laps serum assay in an automated Elecsys device (Roche™, Indianapolis, IN, USA). The inter-assay coefficient of variation was 5%.

2.5 Evaluation of Bone Mineral Density

The adolescents underwent bone densitometry through dual-energy X-ray absorptiometry (DXA) (Hologic QDR 4500 Discovery A, Hologic, Inc., Bedford, MA, USA). The bone mass results were analyzed using appropriate pediatric software. Bone mineral content is expressed in g and BMD in g/cm^2. Measurements were obtained from the L1–L4 lumbar spine region, the total proximal femur including the femoral neck and trochanteric and intertrochanteric regions, and total and subtotal body (whole body less head). Whole body densitometry was performed to obtain total bone mineral content, BMD, and whole body composition. The effect of body size on bone mass can lead to misinterpretations when comparing individuals of different heights and body compositions. In the current study, we sought to minimize body size-induced biases in bone mass estimates by using BMD transformed by body weight (g/cm^2/kg body weight) [28]. All assessments were performed by only one technician trained for this purpose who had no knowledge of the research. In addition, the instructions of the manufacturer of the device and the standards recommended by the International Society for Clinical Densitometry [29] were followed.

The DXA instrument was calibrated by daily scanning of a hydroxyapatite spine phantom. Machine drift was not observed during the study. The coefficient of variation was estimated from repeated measurements (twice) obtained from 30 patients representative of the clinic's patient population for all regions mentioned (lumbar spine and total body) after each patient had been repositioned before scanning. The results demonstrated CVs of 0.6% and 1.3% for the lumbar spine and whole body, respectively. All evaluations were performed by the same blinded, experienced technician, who also performed the densitometry examinations.

2.6 Statistical Analysis

Data were stored in Excel and analyzed using SAS for Windows v.9.2 software. Normality of the quantitative variables was verified by the Shapiro-Wilk test. For characterization of the sample, descriptive analysis of the data was performed by calculating the distribution of frequencies and measures of central tendency and dispersion. The Student t-test was used for the comparison of chronological and bone age, anthropometric variables (weight, height, BMI), BDM and transformed BMD, and bone markers between adolescents with and without MetS and its components, stratified by sex. The frequencies of pubertal stages according to sex were compared between adolescents with and without MetS using the chi-square test.

Pearson's correlation was used to evaluate the correlation between serum levels of the markers and variables related to BMD and MetS components. All analyses were performed separately for each sex. ~~Analysis of variance (ANOVA) was applied to compare mean OC, BAP, and S-CTx levels according to the number of MetS components~~

for each sex, followed by Tukey's multiple comparisons test. Statistical significance was accepted for all analyses when p≤0.05.

3. Results

Forty-eight (57%) of the 84 adolescents were female. The mean age was 12 years among girls and 13 years among boys, both with an average bone age advancement of one year. Among the adolescents, 77.4% were in mid- and late puberty. The adolescents with MetS had higher weight and BMI than those without MetS (p≤0.05). No significant differences in chronological age, bone age, height, or sexual maturation stage were observed between the matched groups with and without MetS for each sex (Table 1).

INSERT TABLE 1

Regarding the MetS components, male and female adolescents with MetS exhibited a significant increase in waist circumference, serum triglyceride levels, and systolic and diastolic blood pressure. Serum HDL-c levels were lower and significant in the groups with MetS. There were statistically significantly lower concentrations in the three bone biomarkers in female adolescents with excess weight and MetS compared to the matched group. In male adolescents, the mean concentrations of BAP, OC, and S-CTx were also lower in those with MetS, but a significant difference was only observed for BAP and S-CTx (Table 2).

INSERT TABLE 2

Pearson's correlation coefficients revealed a negative and significant correlation between BMD at all sites and the three bone biomarkers in female adolescents with MetS (Table 3). No significant correlation was observed between BMD and the bone biomarkers in male adolescents (Table 4).

INSERT TABLE 3 AND 4 NEAR HERE

When the same analysis was performed separately for each MetS component among female adolescents with MetS, the increase in waist circumference was negatively correlated with S-CTx, the increase in blood pressure was negatively correlated with BAP and OC, and the presence of hypertriglyceridemia was positively correlated with BAP and S-CTx. In male adolescents with MetS, the correlation was negative and significant only between increased triglycerides and OC. In adolescents without MetS, a positive and significant correlation was only observed between systolic blood pressure and S-CTx (Table 5).

INSERT TABLE 5

4. Discussion

Comparisons of adolescents with excess weight, with and without MetS, who were similar in terms of chronological age, bone age, and pubertal stage, presented a difference in the concentration of the bone biomarkers between the two groups. In this respect, adolescents with MetS exhibited significantly lower concentrations of bone biomarkers (BAP, OC, S-CTx) than those without MetS, except for OC in male adolescents. In addition, BMD in females was negatively correlated with all bone biomarkers in adolescents with MetS.

~~It is important to note that BAP, OC, and S-CTX are explanatory variables and are correlated. After analysis of correlation and collinearity, it was observed in females, with the presence or absence of MetS, that the analyzed bone markers presented significant correlations and collinearity was affirmed. In the case of markers obtained between male adolescents without MetS, the correlations were not significant and there was no collinearity. The same result was obtained for boys with MetS. Analyzing the~~

~~bone markers for the total group of 84 adolescents of both sexes, it can be observed that the correlations between the BMTs are significant and there is collinearity.~~

Bone mass is acquired gradually during childhood. Acceleration in height and weight growth velocities is observed at the beginning of puberty, the so-called pubertal growth spurt, which is also reflected in bone mass acquisition [30]. According to the National Osteoporosis Foundation, the age group in which peak velocity of bone acquisition is achieved is approximately 12.5 ± 0.90 years for female adolescents and 14.1 ± 0.95 years for male adolescents [30]. Following this international recommendation, the adolescents in our study are probably at the opportune age for maximum peak velocity of bone acquisition, especially the female adolescents.

Researchers have highlighted the importance of understanding the evolution of BMD and alterations in the levels of bone biomarkers during adolescence [31, 32]. Fortes et al. [32], evaluating BMD and its relationship with bone markers in a sample of 101 eutrophic female adolescents of 9 to 20 incomplete years, observed an expressive increase in BMD according to the progression of chronological and bone age. With respect to indicators of bone metabolism, higher mean biomarker levels (BAP=110.12 U/L, OC=26.49ng/mL, S-CTx=1.58 ng/mL) were found when the adolescents were in stage 3 of breast development and in early adolescence (11 to 12 years). The levels of bone biomarkers were lower at the end of adolescence (BAP=31.70 U/L, OC=4.98ng/mL, S-CTx=0.44 ng/mL). Similar results were recently published with the follow-up of 2,416 children and adolescents, from 3 months to 17 years old, analyzing the bone markers Osteocalcin, PINP, and CTX-I. In total, 317 of the participants were obese and the remainder eutrophic [33]. During puberty, the authors demonstrated that the highest levels of bone markers occurred from 10 to 11 years (girls, Tanner 3) and 13 years (boys,

Tanner 3–4). These results are very close to those presented by Fortes et al. [32] both in terms of age and pubertal stage.

In male adolescents, Silva et al. [31] studied a sample of 87 eutrophic adolescents aged 10 to 18 years and concluded that serum biomarker levels differed significantly among age groups. The highest concentration of the biomarkers was observed between 13 and 15 years of age (BAP = 155.50U/L, OC = 41.63 ng/mL, S-CTx = 2.09 ng/mL) and the lowest concentration between 16 and 18 years (BAP= 79.80U/L, OC = 27.80 ng/mL, S-CTx = 1.60 ng/mL). Within this context, proportionally higher levels of bone biomarkers would be expected in the adolescents in this present study, since the mean CA of girls with or without MetS was around 12 years old and BA, around 13 years old, while in boys both CA and BA were around 13 years old. The concentrations obtained among adolescents without MetS in the present study were close to those observed among the eutrophic individuals analyzed in the studies cited [31,32]. However, significant reductions in both biomarkers of bone formation and resorption were observed in the group of adolescents with MetS compared to the matched group without MetS.

Taken together, the results of the present study complement and confirm the findings reported in the literature, showing a significant reduction in BMD at different sites analyzed in adolescents with MetS [5, 6].

Studies in adolescents evaluating the impact of MetS on biomarkers of bone formation and resorption are sparse. In recent years, concern has been directed towards the biomarker OC. In the adult population, researchers found that the accumulation of visceral fat, lipid profile alterations, and altered blood pressure, the main components involved in MetS, were correlated with low serum levels of OC [34]. With respect to the relationship between MetS and OC, Tan et al. [35] evaluated 2,344 Chinese men between

20 and 69 years of age and found a negative association between OC levels and the presence of MetS. With respect to the relationship between MetS and OC, negative association between OC levels and the presence of MetS. The serum concentrations of OC were reduced in subjects with hyperglycemia, hypertriglyceridemia, and low HDL-c. Furthermore, OC levels decreased with the increasing presence of risk factors for MetS [30]. These results of Tan et al. [30] agree with our findings, however, in our results the reductions in OC were significant and negative only in female adolescents with MetS. Thus, analyzing the concentrations of bone biomarkers and the evolution of BMDs according to the sexes, there was a predominance of significant differences for the female sex, with the presence and absence of MetS, which may be related to the nutritional evaluation and biochemical alterations observed between them. In the group of those without the presence of Metabolic Syndrome, 42% of the patients presented no biochemical alterations (HDL-c, triglycerides, and fasting glucose) and weight (kg) and BMI (kg/m^2) (46%) between ≥85th percentile and <95th percentile when compared to those with the presence of MetS. On the other hand, in the group of adolescent males, it was observed, even among those without MetS, that 83% presented biochemical alterations (HDL-c, triglycerides, and fasting glucose) and weight (kg) and BMI (kg/m^2) higher or equal to the 95th percentile were observed in 55.5% of them. This finding possibly influenced the lack of detection of significant differences for the set of biomarkers among male adolescents, when the MetS and absence of MetS groups were compared since, although they were not classified with presence of MetS, they were much more compromised metabolically and nutritionally than the female adolescents.

Our data corroborate and complement the findings of Kim et al. [4] who detected significantly lower levels of OC in overweight and obese Korean children compared to

~~the mean levels obtained for eutrophic children. Analysis of the relationship of OC with adiposity revealed a negative correlation.~~

The literature states that Osteocalcin, besides being a bone-derived hormone, is also associated with energy metabolism. This association is negative (inverse) against hyperglycemia, insulin resistance, hypertriglyceridemia, HDL-c reduction, etc. [35], confirming the connection between bone and energy metabolism [36]. In addition, BAP is also associated with MetS [37], with a strong association with hyperinsulinemia and insulin resistance.

~~In several studies performed with adults, with a cross-sectional or longitudinal design, a reduction in OC was either carboxylated or undercarboxylated in those who had MetS independently of BMI. These same findings are observed in adults with Type 2 Diabetes Mellitus, in individuals with elevated BMI. Thus, it is observed that higher levels of [of carboxylated and undercarboxylated] OCs were associated with lower BMI, a lower percentage of body fat, less insulin resistance, lower HbA1c, as well as lower blood glucose levels and lower levels of inflammatory markers. It should be emphasized that other studies have not been able to prove these associations, hence the need to develop further research, which is carried out across the age spectrum and for each of the sexes, since the available results may be subject to confounding factors [31].~~

~~Bone biomarkers provide active information about bone turnover, reflecting the process of bone formation and resorption. In this respect, reduced levels of OC and BAP indicate lower bone formation [32]. In addition, circulating non-carboxylated OC may stimulate the proliferation of pancreatic beta cells and insulin secretion. Non-carboxylated OC also acts on adipocytes, inducing the synthesis of adiponectin, and plays a preponderant role in the development of metabolic disorders [33].~~

Considering the high prevalence of osteoporosis, obesity, and MetS in adults, Laurent et al. [38] evaluated the relationship of MetS with bone turnover in 3,129 men between 40 and 79 years of age. MetS was present in 975 men (31.2%). The men with MetS demonstrated significantly lower levels of all bone biomarkers and higher BMD at the hip, femoral neck, and lumbar spine. When the MetS components were analyzed individually, only hypertriglyceridemia and hyperglycemia were inversely associated with N-terminal propeptide of type I procollagen (PINP) and S-CTx. Osteocalcin was independently and inversely associated only with hyperglycemia. Men with three, four, or five MetS criteria had lower bone turnover markers. On the other hand, men with one or no criteria presented higher bone turnover markers, indicating the absence of a limit of three criteria above which these markers would be altered. The authors concluded that MetS is associated with lower bone renewal in adult men.

Complementing the small number of publications involving humans, Wong et al. [39] conducted an experimental study to evaluate the effects of MetS induced by a high-carbohydrate high-fat (HCHF) diet on BMD, histomorphometry, and bone markers (OC and S-CTx) in male rats. Twelve Wistar rats (12 weeks old) were randomized into two groups. The normal group received standard chow and the other group received the HCHF diet to induce MetS. There were no significant differences in BMD between normal and HCHF rats ($p>0.05$). The animals fed the HCHF diet had a lower osteoblast surface, higher eroded surface, and lower trabecular bone volume. A significant increase in serum S-CTx levels was observed but no difference in serum OC levels when compared to normal rats. The HCHF diet-induced MetS caused an imbalance in bone remodeling, which led to deterioration of the trabecular bone structure [39].

In agreement with the results of the cited studies, in a population of adolescents, it can be stated that MetS alters bone remodeling [3-5] but not all MetS components show similar associations.

Evidence suggests that MetS favors the differentiation of adipocytes and suppresses the differentiation of osteoblasts, reducing bone formation activity. Furthermore, chronic inflammation induced by MetS can increase the formation of osteoclasts and consequently their bone resorption activity [40, 41]. However, in the present study there was a reduction in the concentrations of all bone markers analyzed in both sexes among adolescents with MetS, suggesting an aggression to bone formation and resorption, possibly indicating a reduction in remodeling.

~~Understanding the physiopathology of MetS is complex and suggests that the interaction between central obesity, insulin resistance, and inflammatory state plays an important role in the genesis of this disease [2]. The increase in adipose tissue is responsible for a proinflammatory state characterized by the increased release of cytokines (interleukins and tumor necrosis factor alpha – TNF-α) and decreased production of adiponectin, reducing insulin sensitivity. In an attempt to confirm these hypotheses, Kurokawa et al. [39] showed that adolescents with excess weight and MetS have higher levels of IL-1 beta, IL-6, and IL-10 than adolescents with excess weight without MetS [39].~~

With respect to MetS and bone remodeling, studies have reported a chronic inflammatory response caused by inflammatory cytokines (IL-1α, IL-1β, IL-6, and TNF-α) produced by visceral adipose tissue in obese individuals, which was associated with the process of osteoclastogenesis when bone resorption would be higher. This chronic inflammatory response contributes to the reduction in bone mass growth during puberty

in obese adolescents [42]. In addition, the presence of insulin resistance, proposed as the essential diagnostic condition of MetS, together with the increase in central adiposity, results in a reduction in adiponectin [43]. Osteoblasts possess receptors for adiponectin, which stimulate the proliferation, differentiation, and mineralization capacity of these cells and inhibit the activity of osteoclasts, consequently reducing bone resorption [44].

Based on animal models of leptin deficiency or deficient leptin signaling, some researchers conclude that this adipokine is a potent inhibitor of bone metabolism [45]. High concentrations of leptin are known to suppress bone formation and stimulate bone resorption. The two leptin-related mechanisms are mediated by RANKL, one related to the action of leptin on hypothalamic receptors, increasing the expression of RANKL and consequently bone resorption, and the other through cocaine- and amphetamine-regulated transcription, which results in the inhibition of RANKL [46]. A recent study indicated negative associations between leptin and OC, suggesting that the inflammatory state may negatively influence bone turnover markers in obese adolescents [47].

There is a limited number of prospective trials, either cohort or clinical, and descriptive studies investigating the evolution of bone biomarkers in adolescents with excess weight and with a diagnosis of MetS. To our knowledge, this is the first study demonstrating the impact of MetS on bone remodeling in adolescents. Thus, in view of the scarce results in the literature, the combined analysis of biochemical markers of bone formation and resorption in a matched sample selected using rigorous inclusion criteria can be considered a strength of the present study. Regarding the cross-sectional design of the study, this prevents the provision of information about the temporal sequence of events and does not permit identification of causal relationships. ~~However, given the evidenced associations and knowledge incorporated in many years of monitoring~~

adolescents with excess weight, obese, with Metabolic Syndrome, analyzing their bone mass by performing densitometry in several places [5]. Previous studies demonstrate an involvement in bone mass between those who are obese and severe/extremely obese and those with the presence of MetS [5, 48], thus we propose that bone turnover markers quickly reveal, in real time, an accompaniment that is only proven by densitometry over a longer period of time, even if it is accepted as the gold standard in bone mass evaluation. It is clear that the recommendation to perform both procedures in adolescents remains desirable, recommended, and complementary.

The current study has some limitations, among which we did not evaluate the pro and anti-inflammatory cytokines of adolescents, which are possibly involved in MetS. In addition, serum levels of 25-hydroxyvitamin D (s25 (OH) D) were not measured, and a deficiency/insufficiency in this biomarker has been described in association with the presence of MetS components in children [49], adolescents, and adults [50]. However, this study contributed by suggesting possible designs for future studies, especially prospective studies, with the inclusion of a larger number of subjects and the possibility of generalizing the results.

In summary, the inclusion and interpretation of this set of bone biomarkers and of others to be incorporated in the future, will complement and broaden knowledge about bone metabolism in adolescents with and without MetS.

5. Conclusion

Our results suggest that adolescents with excess weight and the presence of MetS presented lower concentrations of biomarkers of bone formation and resorption. The presence of MetS could be detrimental to the development of bone mass in adolescents.

The reduction in BMD and in the markers responsible for bone formation during this phase of life may compromise the acquisition of peak bone mass, which could increase the current fracture risk and result in bone microarchitecture derangement and bone fragility in the years to come.

Acknowledgements: Supported by FAPESP (Fundação de Amparo à Pesquisa do Estado de São Paulo) – Grants (07/07731-0, 2011/05991-0 and 2015/04040-2) and the Pro-Rector for Research of UNESP.

References

1. Alhazidou E, Pergialiotis V, Chrelias C, Hatziagelaki E, Papantoniou N, Trakakis E. The impact of the metabolic syndrome on bone mass density: a prospective case control study. Horm Mol Biol Clin Investig. 2017; 33:1-6.

2. Al-Hamad D, Raman V. Metabolic syndrome in children and adolescents. Transl Pediatr. 2017; 6:397-407.

3. Silva VND, Fiorelli LNM, Silva CC, Kurokawa CS, Goldberg TBL. Do Metabolic syndrome and its components have an impact on bone mineral density in adolescents? Nutrition Metabolism (Lond). 2017; 14:1-7.

4. Pollock NK, Bernard PJ, Gutin B, Davis CL, Zhu H, Dong YMD. Adolescent obesity, bone mass, and cardiometabolic risk factors. J Pediatr. 2011; 158:727-734.

5. Nóbrega da Silva V, Goldberg TBL, Mosca LN, Rizzo ACB, Teixeira AS, Corrente JE. Metabolic syndrome reduces bone mineral density in overweight adolescents. Bone. 2014; 66:1-7.

6. Hethering-Rauth M, Bea JW, Blew RM, Funk JL, Lee VR, Roe DJ, Sardinha LB, Going SB. Relationship of cardiometabolic risk biomarkers with DXA and pQCT bone health outcomes in young girls. Bone. 2019; 120:452-458.

7. Lee K. Sex-Specific Relationships between Insulin Resistance and Bone Mineral Content in Korean Adolescents. J Bone Miner Metab. 2013; 31: 177–182.

8. Lawlor DA, Sattar N, Sayers A, Tobias JH. The association of fasting insulin, glucose, and lipids with bone mass in adolescents: findings from a cross sectional study. J Clin Endocrinol Metab. 2012; 97: 2068–2076.

9. Naylor K, Eastell R. Bone turnover markers: use in osteoporosis. Nat Rev Rheumatol 2012.8:379-89.

10. Seibel MJ. Bone turnover in nutrition-related disorders. Wien Med Wochenschr. 2007; 157: 582-588.

11. Kim GS, Jekal Y, Kim HS, Im JA, Park JY, Chu V. Reduced serum total osteocalcin is associated with central obesity in korean children. Obes Res Clin Pract. 2014; 8:201-208.

12. Garanty-Bogacka B, Syrenicz M, Rac M, Krupa B, Czaja-bulsa G, Walczak M, Sowinska-Przepier E, Syrenicz. Association between serum osteocalcin, adiposity and metabolic risk in obese children and adolescents. Endokrynol Pol. 2013; 64:346-352.

13. Kuczmarski RJ, Ogden CL, Guo SS, Grummer-Strawn LM, Flegal KM, Mei Z, Wei R, Curtin LR, Roche AF, Jhonson CL 2000. CDC Growth Charts for the United States: Methods and Development. Vital Health Stat. 2002; 11 246: 1-190.

14. Institute of medicine (IOM), Dietary reference intakes for energy, carbohydrate, fiber, fat, fatty acids, cholesterol, protein, and amino acids (macronutrients). Washington DC: National Academies Press, 2005.

15. Rapuri PB, Gallagher JC, Kinyamu HK, Ryschon KL. Caffeine intake increases the rate of bone loss in elderly women and interacts with vitamin d receptor genotypes. Am J Clin Nutr. 2001; 74:694-700.

16. Fitzpatrick L, Heaney RP. Got soda? J Bone Miner Res. 2003; 18:1570–1572.

17. Mcgartland C, Robson PJ, Murray L. Carbonated soft drink consumption and bone mineral density in adolescence: the Northern Ireland young hearts project. J Bone Mineral Res. 2003; 18:1563–1569.

18. Kuczmarski RJ, Ogden CL, Guo SS, Grummer-Strawn LM, Flegal KM, Mei Z, Wei R, Curtin LR, Roche AF, Jhonson CL 2000. CDC Growth Charts for the United States: Methods and Development. Vital Health Stat. 2002; 11 246: 1-190.

19. Barlow SE, Expert Committee. Expert committee recommendations regarding the prevention, assessment, and treatment of child and adolescent overweight and obesity: summary report. Pediatrics. 2007; 120: Suppl 4:S164-92.

20. Xanthakos SA, Inge TH. Extreme pediatric obesity: weighing the health dangers. J Pediatr. 2007; 150:3-5.

21. National Health and Nutrition Examination Survey (NHANES). Anthropometry procedures manual. Examination Survey, 2017.

22. Sinaiko AR, Steinberger J, Moran A, Hong CP, Prineas RJ, Jacobs DR Jr. Influence of insulin resistance and body mass index at age 13 on systolic blood pressure,

triglycerides, and high-density lipoprotein cholesterol at age 19. Hypertension. 2006; 48:730-736.

23. Marshall WA, Tanner JM. Variations in pattern of pubertal changes in girls. Arch Dis Child. 1969; 44:291-303.

24. Marshall WA, Tanner JM. Variations in the pattern of pubertal changes in boys. Arch Dis Child. 1970; 45:13-23.

25. Finkelstein JW. The endocrinology of the adolescent. Symposium of adolescent medicine. Pediatrics Clinic of North America.1980; 27:53-69.

26. Greulich W, Pyle S. Radiographic atlas of skeletal development of hand and wrist. 2nd ed. Stanford: Stanford University Press, 1959.

27. Zimmet P, Alberti KG, Kaufman F, Tajima N, Silink M, Arslanian S, Wong G, Bennett P, Shaw P, Caprio S, IDF CONSENSUS GROUP (2007). The metabolic syndrome in children and adolescents–an IDF consensus report. Pediatr Diabetes 8:299-306.

28. Hill KM, Braun MM, Egan KA, Martin BR, McCabe LD, Peacock M, et al. Obesity augments calcium-induced increases in skeletal calcium retention in adolescents. J Clin Endocrinol Metab. 2011; 96:2171-7.

29. Gordon CM, Leonard MB, Zemel BS. 2013 Pediatric Position Development Conference: executive summary and reflections. J Clin Densitom. 2014; 17:219-224.

30. Weaver CM, Gordon CM, Janz KF, Kalkwarf HJ, Lappe JM, Lewis R, Okarma M, Wallace TC, Zemel BS. The national osteoporosis foundation's position statement on

peak bone mass development and lifestyle factors: a systematic review and implementation recommendations. Osteoporos Int. 2016; 27:1281-1386.

31. Silva CC, Kurokawa CS, Nga HS, Moretto MR, Dalmas JC, Goldberg TBL. Bone metabolism biomarkers, body weight, and bone age in healthy Brazilian male adolescents. J Pediatr Endocrinol Metab. 2012; 25: 479-484.

32. Fortes CMT, Goldberg TBL, Kurokawa CS, Silva CC, Moretto MR, Biason TP, Teixeira AS, Nunes HRC. Relação entre as idades cronológica e óssea e o estágio puberal das mamas com os biomarcadores ósseos e a densidade mineral óssea em adolescentes. J Pediatr (Rio J). 2014; 90:624-631.

33. Geserick M, Vogel M, Eckelt F, Schlingmann M, Hiemisch A, Baber R, Thiery J, Korner A, Kiess W, Kratzsch J. Children and adolescents with obesity have reduced serum bone turnover markers and 25-hydroxyvitamin D but increased parathyroid hormone concentrations - Results derived from new pediatric reference ranges. Bone. 2020;132:115-124.

34. Bae SJ, Choe JW, Chung YE, Kim BJ, Lee SH, Kim HY, Koh JM, Kim HK, Kim GS. The association between serum osteocalcin levels and metabolic syndrome in Koreans. Osteoporos Int. 2011; 22:2837- 2846.

35. Tan A, Gao Y, Yang X, Zhang H, Qin X, Mo L, Peng T, Ning X, Mo Z. Low serum osteocalcin level is a potential marker for metabolic syndrome: results from a Chinese male population survey. Metabolism. 2011; 60:1186-1192.

36. Fernandes TAP, Gonçalves LML, Brito JAA. Relationships between Bone Turnover and Energy Metabolism. Journal of Diabetes Research.2017; 27: 1.

37. Cheung CL, Tan KC, Lam KS, Cheung BM. The relationship between glucose metabolism, metabolic syndrome, and bone-specific alkaline phosphatase: a structural equation modeling approach. J Clin Endocrinol Metab. 2013.9:3856-63.

38. Laurent MR, Cook MJ, Gielen E, Ward KA, Antonio L, Adams JE, Decallone B, Bartfai G, Casanueva FF, Forti G, Giwercman A, Huhtaniemi IT, Kuka K, Lean MEJ, Lee DM, Pendleton N, Punab M, Claessens F, Wu FCW, Vanderschueren D, Pye SR, O'Neill TW, EMAS GROUP. Lower bone turnover and relative bone deficits in men with metabolic syndrome: a matter of insulin sensitivity? The European Male Ageing Study, Osteoporos Int. 2016; 27:3227–3237.

39. Wong SK, Chin KY, Suhaimi FH, Ahmad F, Ima-Nirwana S. Effects of metabolic syndrome on bone mineral density, histomorphometry and remodeling markers in male rats. PLoS One. 2018; 13:e0192416.

40. Beederman M, Lamplot JD, Nan G, Wang J, Liu X, Yin L, Li R, Shui W, Zhang H, Kim SK, Zhang W, Zhang J, Kong Y, Denduluri S, Rogers MR, Pratt A, Haydon RC, Luu HH, Angeles J, Shi LL, He TC. BMP signaling in mesenchymal stem cell differentiation and bone formation. J Biomed Sci Eng. 2013; 6(8A):32-52.

41. Felice JI, Gangoiti MV, Molinuevo MS, Mccarthy AD, Cortizo AM. Effects of a Metabolic Syndrome induced by a fructose-rich diet on bone metabolism in rats. Metabolism. 2014;63: 296-305.

42. Cao JJ. Effects of obesity on bone metabolism. J Orthop Surg Res. 2011; 30:1-7.

43. Rubin DD, Mcmurray RR, Harrell JS, Hackney AC, Hagg AM. Do surrogate markers for adiposity relate to cytokines in adolescents? J investig Med. 2008; 56:786-792.

44. Yamaguchi T, Sugimoto T. Bone metabolism and fracture risk in type 2 diabetes mellitus. Bonekey Rep. 2012; 1:36.

45. Elefteriou F, Ahn JD, Takeda S, Starbuck M, Yang X, Liu X, Kondo H,. Richards WG, Bannon TW, Noda M, Clement K, Vaisse C, Karsenty G. Leptin regulation of bone resorption by the sympathetic nervous system and CART. Nature. 2005; 434:514-20.

46. Foo JP, Polyzos SA, Anastasilakis AD, Chou S, Mantzoros CS. The effect of leptin replacement on parathyroid hormone, RANKL-osteoprotegerin axis, and Wnt inhibitors in young women with hypothalamic amenorrhea. J Clin Endocrinol Metab. 2014; 99: E2252–2258.

47. Campos RMDS, Masquio DCL, Corgosinho FC, Carvalho-Ferreira JP, Molin Netto BD, Clemente APG, Tock L, Tufik S, Mello MT, Dâmaso AR. Relationship between adiponectin and leptin on osteocalcin in obese adolescents during weight loss therapy. Arch Endocrinol Metab. 2018; 62:275-284.

48. Mosca LN, Goldberg TBL, Silva VN, Kurokawa CS, Rizzo ACB, da Silva CC, Teixeira AS, Corrente JE. The impact of excess body fat on bone remodeling in adolescentes. Osteoporos Int. 2017. 28:1053–1062.

49.Nwosu BU, Maranda L, Cullen K, Ciccarelli C, Lee MM. Vitamin D status is associated with early markers of cardiovascular disease in prepubertal children. J Pediatr Endocrinol Metab. 2013;26(11-12):1067-75. doi: 10.1515/jpem-2013-0086.

50. Fu J, Han L, Zhao Y, Li G, Zhu Y, Li Y, Li M, Gao S, Willi SM. Vitamin D levels are associated with metabolic syndrome in adolescents and young adults: The BCAMS study. Clin Nutr. 2019.38:2161-2167.

References Removed and Table 1

~~31. Levinger I, Brennan-Speranza TC, Zulli A, Parker L, Lewis JR, Yeap BB. Multifaceted interaction of bone, muscle, lifestyle interventions and metabolic and cardiovascular disease: role of osteocalcin. Osteoporos Int. 2017; 28:2265–2273.~~

~~32. Kini U, Nandeesh BN. Physiology of Bone Formation, Remodeling, and Metabolism. In: Fogelman I, Gnanasegaran G, van der Wall H. (eds) Radionuclide and Hybrid Bone Imaging. Springer, Berlin, Heidelberg, 2012.~~

~~33. Ferron M, Hinoi E, Karsenty G, Ducy P. Osteocalcin differentially regulates A-cell and adipocyte gene expression and affects the development of metabolic diseases in wild-type mice. Proc Natl Acad Sci. 2008; US A 105:5266-5270.~~

~~36. Von Muhlen D, Safil S, Jassal SK, Savartberg J, Barrett-Connor E. Associations between the metabolic syndrome and bone health in older men and women: the Rancho Bernardo Study. Osteoporos Int. 2007; 18:1337–1344.~~

~~39. Kurokawa CS, Goldberg TBL, Nóbrega V, Medeiros LTL, Betti IR, Lima GR, Romagnoli GG, Oliveira LR, Gonçalvez B, Rizzo ACB. Excess weight in adolescents is associated with increased levels of inflammatory cytokine. In: 4TH Annual Meeting of the International Cytokine and interferon society (ICIS). San Francisco, USA, 2016;136-137.~~

Table 1. Definition of metabolic syndrome in adolescents aged 10 to 16 years according to International Diabetes Federation criteria.

Waist Circumference	*Triglycerides*	*HDLc*	*Blood pressure*	*Glusose*
≥90th percentile	≥150 mg/dL	<40mg/dL	SBP≥130mmHg or DBP≥85mmHg	≥100 mg/dL or known DM2

WC: waist circumference; HDLc: high-density lipoprotein-cholesterol; SBP, systolic blood pressure; DBP, diastolic blood pressure; DM2: type 2 diabetes mellitus.

Funding: FAPESP (Fundação de Amparo à Pesquisa do Estado de São Paulo) – Grants (07/07731-0, 2011/05991-0 and 2015/04040-2).

Availability of data and materials: Data that support the findings of this study are available from the corresponding author upon reasonable request.

Ethics approval and consent to participate: The study was approved by the Ethics Committee of the Botucatu Medical School (FMB-UNESP) (Approval No. 1.399.709 /2016).

Consent for publication: Prior to data collection, the purpose of the study was explained to the participants and their informed consent was recorded.

Authors' contributions: All authors have read and agreed to the publication of this paper. V.N.S. and T.B.L.G. wrote the first draft and, together with L.N.M.F., C.S.K., C.C.S., and A.C.B.R. contributed equally to this study by providing opinions and constructive criticism regarding the content. José Eduardo Corrente was the statistical consultant.

Competing interests: The authors declare that they have no competing interests.

Table 1. Comparison of adolescents with and without metabolic syndrome according to sex, bone age, tanner stage, anthropometric measures, body mass index, BMD, and transformed BMD of both genders.

**Parameter**		**Female (n=48)**			**Male (n=36)**		
		**MetS (-) (n=24)**	**MetS (+) (n=24)**		**MetS (-) (n=18)**	**MetS (+) (n=18)**	
		**Mean ± SD**		***p* value[a]**	**Mean ± SD**		***p* value[a]**
Age (years) [a]		11.78±1.29	12.58±1.95	0.102	13.03±1.30	13.42±1.23	0.393
Bone age (years)[a]		13.09±1.75	13.40±2.07	0.582	13.73±0.92	13.86±1.88	0.813
Tanner stage [n (%)][b]		***Breast development***			***Genital***		
Early puberty	1<br>2	1 (4)	2 (9)		2 (14)	2 (14)	
Mid-puberty	3	8 (35)	6 (29)	0.690	5 (33)	4 (29)	0.994
Late puberty	4<br>5	14 (61)	13 (62)		8 (53)	8 (57)	
Weight (kg)[a]		58.85±9.46	73.58±13.92	<0.000*	71.32±14.58	81.10±13.44	0.044*
Height (m)[a]		1.54±0.07	1.57±0.07	0.167	1.63±0.09	1.64±0.09	0.728
BMI (kg/m^2)[a]		24.61±2.64	29.32±4.16	0.038*	26.44±4.22	29.61±4.21	0.030*
Lumbar spine BMD (g/cm^2)		0.82±0.15	0.90±0.19	0.123	0.77±0.12	0.85±0.13	0.052
Femoral BMD (g/cm^2)		0.89±0.12	0.95±0.14	0.124	0.95±0.16	1.00±0.14	0.323
Total body BMD (g/cm^2)		0.90±0.09	0.94±0.10	0.162	0.92±0.08	0.94±0.09	0.609
Subtotal body BMD (g/cm^2)		0.81±0.08	0.85±0.09	0.150	0.84±0.09	0.86±0.09	0.560

			BMD transformed for body weight (g/cm^2/kg body weight).			
Lumbar spine BMD	0.0141±0.0021	0.012±0.002	0.005*	0.011±0.002	0.010±0.001	0.416
Femoral BMD	0.0154±0.0024	0.013±0.001	0.001*	0.013±0.002	0.012±0.002	0.153
Total body BMD	0.0156±0.0023	0.013±0.001	<0.000*	0.013±0.002	0.011±0.002	0.042*
Subtotal body BMD	0.0141±0.0020	0.011±0.001	<0.000*	0.012±0.001	0.010±0.002	0.050*

MetS (−): without metabolic syndrome; MetS (+): with metabolic syndrome.
BMI, Body Mass Index.
[a] Student t-test. [b] Chi-square test.

Table 2. Comparison of metabolic syndrome components and bone biomarkers between adolescents with and without MetS according to sex.

Parameter	Female (n=48)			Male (n=36)		
	**MetS (-) (n=24)**	**MetS (+) (n=24)**		**MetS (-) (n=18)**	**MetS (+) (n=18)**	
	Mean ± SD		*p* value[a]	Mean ± SD		*p* value[a]
MetS component						
WC (cm)	83.70±7.86	94.94±7.48	<.000	86.80±7.35	92.68±9.56	0.051
HDL-c (mg/dL)	46.60±10.83	39.62±9.41	0.022	43.77±7.89	36.27±3.21	0.008
SBP (mmHg)	104.70±11.06	117.30±13.64	0.001	116.60±9.84	128.40±13.70	0.005
DBP (mmHg)	63.77±6.79	73.89±8.09	<.000	69.58±5.86	80.79±9.00	0.000
Triglycerides (mg/dL)	88.82±19.80	149.90±52.03	<.000	99.38±41.74	172.60±72.35	0.000
Fasting glucose (mg/dL)	82.78±6.19	86.75±8.96	0.085	87.83±6.90	87.22±7.58	0.891
Bone biomarker						
BAP (U/L)	178.10±46.26	121.50±65.68	0.001	244.10 ±56.96	179.70±77.46	0.008
OC (ng/mL)	39.08±16.10	21.02±12.15	<.000	40.07±12.52	31.03±15.59	0.066
S-CTx (ng/mL)	1.56±0.52	1.22±0.46	0.027	1.93±0.33	1.60±0.53	0.034

(−): MetS components without alteration; (+): MetS components with alteration.

WC, waist circumference; HDL-c, HDL-cholesterol; SBP, systolic blood pressure; DBP, diastolic blood pressure; BAP, bone alkaline phosphatase; OC, osteocalcin; S-CTx, carboxy-terminal telopeptide

[a] Student t-test.

Table 3. Pearson's correlation between bone biomarkers and bone mineral density measured at four sites in female adolescents with and without metabolic syndrome.

**Parameter**		**MetS (-)**			**MetS (+)**		
		**BAP (U/L)**	**OC (ng/mL)**	**S-CTx (ng/mL)**	**BAP (U/L)**	**OC (ng/mL)**	**S-CTx (ng/mL)**
Lumbar Spine BMD (g/cm^2)	*r*	-0.04088	-0.09311	0.04937	-0.71742	-0.53505	-0.51012
	p	0.873	0.665	0.818	<.000	0.007	0.018
Femoral BMD (g/cm^2)	*r*	-0.03186	-0.07059	0.09377	-0.52271	-0.68411	-0.54997
	p	0.882	0.743	0.663	0.008	0.000	0.009
Total body BMD (g/cm^2)	*r*	-0.04088	-0.16939	0.10861	-0.55568	-0.81760	-0.53838
	p	0.8496	0.428	0.613	0.005	<.000	0.011
Subtotal Body BMD (g/cm^2)	*r*	-0.04533	-0.18300	0.12481	-0.79215	-0.47089	-0.54338
	p	0.833	0.392	0.561	<.000	0.023	0.010

MetS (−): without metabolic syndrome; MetS (+): with metabolic syndrome.
BMD, bone mineral density; BAP, bone alkaline phosphatase; OC, osteocalcin; S-CTx, carboxy-terminal telopeptide.
Pearson correlation test.

Table 4. Pearson's correlation between bone biomarkers and bone mineral density measured at four sites in male adolescents with and without metabolic syndrome.

**Parameter**		**MetS (-)**			**MetS (+)**		
		**BAP (U/L)**	**OC (ng/mL)**	**S-CTx (ng/mL)**	**BAP (U/L)**	**OC (ng/mL)**	**S-CTx (ng/mL)**
Lumbar spine BMD	r	-0.42803	0.02406	-0.07669	-0.09165	0.30822	0.01061
(g/cm^2)	*p*	0.076	0.924	0.762	0.726	0.228	0.970
Femoral BMD	r	-0.20502	0.2714	0.22064	-0.03393	0.30356	-0.03041
(g/cm^2)	*p*	0.414	0.274	0.379	0.897	0.236	0.914
Total body BMD	r	-0.31354	0.22305	-0.08190	-0.07856	0.22114	-0.14209
(g/cm^2)	*p*	0.205	0.373	0.746	0.764	0.393	0.613
Subtotal body BMD	r	-0.30173	0.22308	-0.08440	-0.05501	0.15457	-0.08828
(g/cm^2)	*p*	0.223	0.373	0.739	0.8339	0.5536	0.7544

MetS (−): without metabolic syndrome; MetS (+): with metabolic syndrome.
BMD, bone mineral density; BAP, bone alkaline phosphatase; OC, osteocalcin; S-CTx, carboxy-terminal telopeptide.
Pearson correlation test.

Table 5. Pearson's correlation between bone biomarkers and metabolic syndrome components in adolescents of both sexes with and without the syndrome.

MetS component		**MetS (-)**			**MetS (+)**		
		**BAP (U/L)**	**OC (ng/mL)**	**S-CTx (ng/mL)**	**BAP (U/L)**	**OC (ng/mL)**	**S-CTx (ng/mL)**
				Female			
WC (cm)	r	-0.29836	-0.07739	-0.24094	-0.32473	-0.24808	-0.46752
	p	0.177	0.732	0.280	0.121	0.242	**0.032***
HDL-c (mg/dL)	r	0.13497	-0.0581	-0.11359	-0.01574	-0.28168	-0.00502
	p	0.539	0.792	0.605	0.941	0.182	0.982
SBP (mmHg)	r	-0.22598	-0.0187	-0.16776	-0.50237	-0.46993	-0.17346
	p	0.311	0.934	0.455	**0.014***	**0.023***	0.464
DBP (mmHg)	r	-0.21424	0.00212	-0.20241	-0.35271	-0.4547	-0.02847
	p	0.338	0.992	0.366	0.098	**0.029***	0.905
Triglycerides (mg/dL)	r	-0.40542	-0.12222	-0.19898	0.46519	0.20343	0.51017
	p	0.055	0.578	0.362	**0.022***	0.340	**0.018***
Fasting glucose (mg/dL)	r	-0.00579	-0.22448	-0.23428	-0.09644	-0.0954	-0.17617
	p	0.979	0.303	0.281	0.654	0.657	0.444
				Male			
WC(cm)	r	0.45455	0.19324	0.11532	0.14272	0.39229	0.19134
	p	0.058	0.442	0.648	0.598	0.132	0.512
HDL-c (mg/dL)	r	0.00244	0.29945	0.05388	-0.33351	-0.18099	-0.07285
	p	0.992	0.227	0.831	0.190	0.487	0.796
SBP (mmHg)	r	0.02191	0.30439	0.34550	0.15801	0.20069	0.37223
	p	0.931	0.219	0.160	0.558	0.456	0.190
DBP (mmHg)	r	0.30301	0.05582	0.53446	0.24027	0.14589	0.26202
	p	0.221	0.825	**0.022***	0.370	0.589	0.365
Triglycerides (mg/dL)	r	-0.20649	-0.11482	0.22251	-0.22418	-0.48347	-0.15625
	p	0.411	0.650	0.374	0.387	**0.049***	0.578
Fasting glucose (mg/dL)	r	-0.19083	-0.1420	0.05144	0.17677	0.13868	-0.14624
	p	0.448	0.568	0.839	0.497	0.595	0.603

MetS (−): without metabolic syndrome; MetS (+): with metabolic syndrome.

BAP, bone alkaline phosphatase; OC, osteocalcin; S-CTx, carboxy-terminal telopeptide; WC, waist circumference; HDL-c, H[illegible] cholesterol; SBP, systolic blood pressure; DBP, diastolic blood pressure. Pearson correlation test.

To Benjamin Udoka Nwosu, MD

Academic Editor

PLOS ONE

Dear Dr. Udoka Nwosu

First, we are grateful that you considered the topic of this manuscript to be an interesting area of exploration in pediatric bone metabolism and for sending your personal comments and the excellent comments from the reviewers regarding our article "*Impact of metabolic syndrome and its components on bone remodeling in adolescents*". We have carefully read and reread the comments to ensure that we have effectively responded to the suggestions and questions raised. Below are the comments made by the reviewers and, in sequence, our answers, highlighted in ***blue*.** The passages added to the original article are also highlighted in blue.

We would like to take this opportunity to thank you for the possibility of re-presenting the study to PLOS One with the incorporation of the comments made by the reviewers.

Comments:

1. The manuscript is quite voluminous and some of the sections such as the Introduction and Discussion should be edited and shortened considerably for clarity.

Answer: We have reduced the two sections as suggested.

2. Authors should attempt to offer an explanation why all the bone turnover markers were decreased in the cohort with MetS.

Answer: An explanation has been added to the discussion.

3. The authors should also endeavor to explain the reason for the negative correlation of all bone markers (both bone formation and bone resorption markers) with BMD.

Answer: Dear Editor, in previous studies from our group with eutrophic adolescents, we observed negative correlations between bone markers and bone mineral density in females. We have written:

*"Bone remodeling biomarkers (BAP, OC, and S-CTx) presented a significant negative correlation with CA, BA, and pubertal stage. This finding demonstrated that the more mature the participants, the higher their BMD values. Through analyses of biomarkers, that study demonstrates the changes in bone remodeling occurring in the second decade of life**, revealing high marker concentrations in the early adolescence years and significantly reduced concentrations in late adolescence**. **These analyses correlate with the BMD values, which***

***represent bone mass incorporation, and indicate an inversely proportional behavior showing the highest BMD values associated with the lowest concentrations of formation and resorption of biomarkers** (*Fortes *et al.*, 2014*).”*

Thus, this negative correlation represents the expected evolution in eutrophic adolescents and, possibly, in those with MetS. However, we highlight that the concentrations of the markers were even lower in the group with MetS and there were stronger correlations between biomarkers and bone mineral density, as shown in the manuscript. **Although the increase in BMD was** less than expected (Nóbrega da Silva et al., 2014), a negative correlation was found only in girls with bone markers. Similar results were found in a previous study (Yilmaz *et al*., 2005) which reported: "*A negative correlation of BMD at two sites with bone formation markers was significant in girls (p< 0.001), but no correlation could be detected in boys (p> 0.05)".*

<u>References:</u>

Fortes CM, Goldberg TB, Kurokawa CS, Silva CC, Moretto MR, Biason TP, Teixeira AS, Nunes HR. Relationship between chronological and bone ages and pubertal stage of breasts with bone biomarkers and bone mineral density in adolescents. J Pediatr (Rio J). 2014;90(6):624-31.

Nóbrega da Silva V, Goldberg TBL, Mosca LN, Rizzo ACB, Teixeira AS, Corrente JE. Metabolic syndrome reduces bone mineral density in overweight adolescents. Bone. 2014; 66:1-7.

Yilmaz, D., Ersoy, B., Bilgin, E. et al. Bone mineral density in girls and boys at different pubertal stages: relation with gonadal steroids, bone formation markers, and growth parameters. J Bone Miner Metab.2005; 23:476–482.

4. The finding of reduced bone formation markers and BMD in MetS subjects suggests that the underlying mechanism or the reduced biomarkers and BMD is most likely due to increased systemic inflammation which was not explored in this study. To establish this, however, will require a study of the patients' pro-inflammatory and anti-inflammatory cytokines which was not done in this study. Therefore, this should be discussed in the section on limitations of the study.

<u>Answer:</u> You are correct. We have removed the text that introduced this possibility about the inflammatory state and presented it as one of the limitations of the present study.

5. It was not clear why the authors did not evaluate vitamin D status in their subjects which could have shed some light on their findings. This is a significant limitation of the study and should be discussed in the section on limitations.

Answer: We agree that this is another limiting factor in the study because 25-hydroxyvitamin D [25 (OH) D] was not dosed. However, as all the adolescents included and evaluated were overweight and the groups were paired, we believe that both groups had Vitamin D insufficiency/deficiency, whether or not they had MetS.

6. Table 1 should be completely redone. Table 1should present the comparison between Mets patients and non-Mets patients for anthropometry and biochemical parameters.

Answer: Dear Editor, Table 1 corresponded to the IDF criteria for the classification of Metabolic Syndrome and has been removed, and the description of the components of Metabolic Syndrome has been added to the manuscript.

The current table 1 is entitled: *Comparison of adolescents with and without metabolic syndrome according to sex, bone age, tanner stage, anthropometric measures, and body mass index,* and BMD and transformed BMD of both sexes have been added.

The bone markers are included in table 2, entitled: *Comparison of metabolic syndrome components and bone biomarkers between adolescents with and without MetS according to sex.*

7. Please review the comments from the reviewers below and provide the anthropometric and radiological data in z-scores where appropriate.

Answer: Dear Editor, each Reviewer's comment has been carefully analyzed and we have provided detailed answers below.

Review Comments to the Author

1.Reviewer #1: Comment 1: The study had a decent cohort size of adolescents matched by age, bone age, and puberty. In addition, it was stratified by sex. The authors compared adolescents with MetS with adolescents without MetS as controls. It would also be interesting how these findings relate to normal weight adolescents. Though evaluation of obesity and MetS and BMD in pediatrics has been published, the authors took this further to attempt to look at affects of MetS in adolescents with excess weight on bone remodeling markers, which is a very interesting and important concepts.

Answer: Dear Reviewer, we appreciate your encouraging comments and your careful analysis of our study. We have tried to answer each question in order to improve the manuscript.

2. The conclusion, objective in introduction worded strongly. BMD and bone mass used interchangeably in the conclusion. BMD data and analyses not provided between the groups.

Answer: You are absolutely right, we included a table without the reported BMD results evaluated in the various analyzed sites. We have changed table 1 and included the BMD means, as well as the transformed BMDs.

The conclusion has been changed to: *The presence of MetS is detrimental to the bone mineral density (BMD) and biochemical markers of bone formation and resorption in adolescents with excess weight.*

3. BMD data was provided in tables showing r correlations with bone resorption markers only. Can the authors show BMD data and analysis done to draw the main discussion and conclusion points?

Answer: We have added the means and SD of the BMD to Table 1. Tables 3 and 4 show BMD and Biomarker values (OC, BAP, and S-CTx) for boys and girls respectively.

4.Was DXA measurements adjusted for height? please explain why or why not.

Answer: Densitometry was **not** adjusted for height, but for weight. This is the same technique that we used in a manuscript that we previously published in BONE (Nóbrega da Silva *et al.*, 2014) citing the study of Hill *et al*. (2011). As the groups were matched for CA, BA, and pubertal development, they did not differ in height, as can be seen in Table 1.

References:

Nóbrega da Silva V, Goldberg TB, Mosca LN, Bisi Rizzo Ada C, Teixeira Ados S, Corrente JE. Metabolic syndrome reduces bone mineral density in overweight adolescents. Bone. 2014;66:1-7.

Hill KM, Braun MM, Egan KA, Martin BR, McCabe LD, Peacock M, et al. Obesity augments calcium-induced increases in skeletal calcium retention in adolescents. J Clin Endocrinol Metab 2011 ;96:2171–7.

5. Authors report BMI, but no BMI %iles or SDS. In pediatric data report the latter to categorize the cohort.

Answer: You are absolutely right about the percentile assessment. This analysis was performed for each of the individuals included in the study, as we explain in the methods. However, due to the number of words, we did not include it in the text or in a table, as we understood that we

would have a lot of data and that this was not the focus of the study, since we only included overweight individuals. We present the nutritional classification according to percentiles of the groups paired by CA, BA with MeTs - and MeTS + only for information, remembering that adolescents with a BMI between the 85th and 95th percentile were classified as overweight, between the 95th and 99th percentile as obese (Kuczmarski, Ogden, Guo, 2002), and above the 99th percentile as extremely obese (Barlow, 2007).

Nutritional evaluation of adolescents matched and included in the study sample.

	**Female (n=48)**		**Male (n=36)**	
	**MetS (-)**	**MetS (+)**	**MetS (-)**	**MetS (+)**
Overweight	11 (45.9%)	4 (16.7%)	8 (44.4%)	3 (16.7%)
Obese	11 (45.9%)	14 (58.3%)	8 (44.4%)	5 (27.8%)
Severe Obese	2 (8.3%)	6 (25.0%)	2 (11.1%)	10 (55.6%)

References:

Kuczmarski RJ, Ogden CL, Guo SS, Grummer-Strawn LM, Flegal KM, Mei Z, Wei R, Curtin LR, Roche AF, Jhonson CL 2000. CDC Growth Charts for the United States: Methods and Development. Vital Health Stat. 2002; 11 246: 1-190.

Barlow SE, Expert Committee. Expert committee recommendations regarding the prevention, assessment, and treatment of child and adolescent overweight and obesity: summary report. Pediatrics. 2007; 120: Suppl 4:S164-92.

6. In the lines 296-298, the authors state that the study showed a significant reduction in BMD at different sites. It is not clear what the data were. The authors should clarify and show BMD data to support the statement.

Answer: We apologize and have changed Table 1 to present the data.

7. Lines 312-319: The authors have an interesting finding pertaining to the sex differences, they speculate that it may be attributable to the differences in biochemical and anthropometric characteristics. It is not clear what biochemical characteristics are being referred to. Similarly, line 322-323, states males were much more compromised metabolically, however, it is not clear in which sense and what metabolic derangements this assessment is being referred to.

Answer: Dear Reviewer, we are referring to each of the nutritional and biochemical criteria used for the diagnosis of MeTS, in this case among the biochemicals HDLc, triglycerides, and

glycemia. Among girls without MeTS, we were able to match with a group in which few biochemical/metabolic alterations were detected in 42% of them.

In the group without the presence of Metabolic Syndrome, 42% of the patients presented no biochemical alterations, and 45.9% demonstrated weight (kg) and BMI (kg/m²) between ≥85th percentile and <95th percentile when compared to those with the presence of MetS where only 16.7% were between these values.

In contrast, for the boys:
On the other hand, in the group of adolescent males, it was observed, even among those without MetS, that 83% presented biochemical alterations (HDLc, triglycerides, and glycemia) and weight (kg) and BMI (kg/m2) higher or equal to the 95th percentile were observed in 55.5% of them. This confirms that even those without MeTS were much more compromised metabolically and nutritionally than the female adolescents.

8. In the conclusion session, authors mention the reduction in BMD and its effects on peak bone mass, however, there are no data presented on BMD values or Z-scores in this cohort.
Answer: We apologize again that we did not present the data. We have now added the missing information to Table 1.

9.MetS increases bone resorption, however, CTX was lower in MetS group indicating lower bone resorption. It is eluded that most likely due to decrease bone remodeling. However, in chronic inflammation states of MetS and obesity osteoclast and bone resorption activities are significantly elevated as been shown in other adults studies and sites by the authors. In addition, authors showed that BMD negatively correlated with CTX. Can authors elaborate further on these rather unexpected findings?
Answer: We also believed that bone resorption should have increased, reflected by an increase in S-CTX, as documented in studies that evaluated adults. However, we should remember that adolescence is a time for increased bone mass and peak bone mass, as close to 42% of bone mass is added during the second decade of life. Therefore, bone formation should overcome resorption. Herein, we did not observe evolution as shown in adults. There was a reduction in the concentrations of all bone markers analyzed in both sexes among adolescents with MetS, suggesting an aggression to bone formation and resorption, possibly indicating a reduction in remodeling.

10.Table 4 appears to have a small shift in the values, which should be an easy edit.
Answer: Thank you, we have corrected this.

11.Table 5 is difficult to read, p values are missing in the comparison of Lumbar spine BMD with the bone makers; instead, r values are provided twice.
Answer: We appreciate your careful evaluation, and have made the corrections.

12.Table 6 is difficult to read and follow, recommend revision. There are multiple comparisons in the study, especially in table 6. It is not clear if correction for multiple comparisons was done. Can authors comment?
Answer: Dear reviewer, this is now Table 5, and it is possible to observe the linear correlations (*Pearson*) between bone biomarkers and the components of the Syndrome. The correlations demonstrate that each Mets component in isolation does not have a significant correlation with bone biomarkers. Thus, the few significant p values have now been highlighted in bold and with an asterisk.

13.Comment 3: Besides tables and results in the manuscript no additional data were provided. BMD data were not provided.
Answer: Thank you, we have made the corrections.

14.Comment 4: Discussion had comprehensive overview and review of available data on this and relevant topics. Many sentences are too long and difficult to follow. Discussion should be revised and edited for clarity.
Answer: We appreciate the considerations and the discussion has been revised and edited.

Line 59: "non-definitively demonstrated" - consider revision
Answer: We appreciate the comment. In the restructuring of the introduction this paragraph was removed.

14. Sentence in lines 261-268 is not clear and seems ambiguous. Please revise the point.
Answer: We accept your suggestion about ambiguity and have restructured the text.

15.In line 268 authors use BMTs, I am assuming it's a typo and they mean BMD. This should be clarified and corrected. There was another area where BMTs were used in place of intended BMD.

Answer: We apologize, but BMTs were used as an abbreviation for Bone Turnover Markers. However, in the current version the problem does not arise.

16.Lines 303-305: the sentence is incomplete. this needs a grammar check.
Answer**:** You are correct, we have corrected the paragraph.

17.The statements in lines 332-338 need references. The sentence in lines 417-424 is confusing and very long, needs revision.
Answer**:** Dear reviewer, we have added the respective references to lines 321-323 (Fernandes, Gonçalves, Brito, 2017; Cheung *et al*., 2013). In the other section, lines 355-357 have been reformulated: *"However, in the present study there was a reduction in the concentrations of all bone markers analyzed in both sexes among adolescents with MetS, suggesting an aggression to bone formation and resorption, possibly indicating a reduction in remodeling".*

References:

Fernandes TAP, Gonçalves LML, Brito JAA. Relationships between Bone Turnover and Energy Metabolism. Journal of Diabetes Research.2017; 27: 1.

Cheung CL, Tan KC, Lam KS, Cheung BM. The relationship between glucose metabolism, metabolic syndrome, and bone-specific alkaline phosphatase: a structural equation modeling approach. J Clin Endocrinol Metab. 2013.9:3856-63.

Reviewer #2: General Comments

18.This appears to be a sub-study of a larger study looking at the impact of metabolic syndrome and its components on bone remodeling in adolescents. The authors present relevant data and the use of biochemical markers of bone turnover is appreciated given the age group and lack of research using bone markers in adolescents particularly in those who are classified as overweight or obese. This data would be a welcome addition to the literature.
Answer: Dear Reviewer, we appreciate your positive comments regarding our study.

20.There are number of general and specific comments that need to be addressed with respect to the interpretation of the bone remodeling data, editing of wording, and the discussion as it is currently written. For these reasons, a major revision is recommended.

As well, the Authors have a paper published in Bone (2014) examining MetS and its effects on bone mass, similarly using 271 participants and study design. Although that paper did not measure bone markers, it is not included in the current reference list even though sections of the methods could refer to it and make it clear if the 271 participants are similar between both papers. It appears the present study is a subset or additional analysis of a larger one that may have already been published. Are the current 84 participants from the original 271 participants or have any of them been additionally recruited? Can the authors provide any information or discussion on the use of or lack thereof a control group (normal weight non-MetS).

Answer: We apologize if it seemed that we had given prominence to our study, published in Bone (2014), as it appears among other references, in the Introduction (Nóbrega da Silva *et al.*, 2014). There are few studies that address the theme in this age group and the current work is the subset of a larger work previously published, where we only analyzed the involvement of bone mineral density among adolescents with MetS. With respect to the inclusion of a control group with normal weight, it did not seem appropriate to conduct this study, considering the factors that could influence bone markers, when individuals are overweight and have MetS. In a previous publication (Mosca *et al.*, 2017), we used a control group with adequate weight, when analyzing bone markers in obese individuals, without classifying them as MetS + or MetS –.

References:

Nóbrega da Silva V, Goldberg TBL, Mosca LN, Rizzo ACB, Teixeira AS, Corrente JE. Metabolic syndrome reduces bone mineral density in overweight adolescents. Bone. 2014; 66:1-7.

Mosca LN, Goldberg TBL, Silva VN, Kurokawa CS, Rizzo ACB, da Silva CC, Teixeira AS, Corrente JE. The impact of excess body fat on bone remodeling in adolescentes. Osteoporos Int. 2017. 28:1053–1062.

21.Much of the discussion is spent on studies and finding relating to OC, however that is not the only bone marker that was measured in the current study. CTx was mentioned briefly and there was no discussion provided to BAP. In addition, the authors provide meaningful information on types of diet, inflammation and cytokines in the discussion, but fail to use this information to support and make connections to the results of their study. Given that inflammatory cytokines or RANKL were not measured in the current study, I would recommend the authors not include these sections, unless they can connect it with the variables

they did in fact measure. I would suggest speaking more to the specific MetS components, the number of them present and addressing S-CTx and BAP in the discussion.

Answer: We appreciate your suggestions, and have made adjustments to the text.

Specific Comments Abstract

21.1. Numerical values or results in terms of r- or p-values should be presented in the abstract.

Answer: Dear reviewer, we accept your suggestion and the abstract now contains the r and p-values.

Introduction

22.1. Define MetS, the conditions it can include, risk factors and how this relates to and presents in adolescents. How many MetS components do adolescents need to be classified as having MetS?

Answer: Dear Reviewer, the concept of MeTS and the recommendations proposed by the IDF, now read as follows in the methods (lines 162 to 167): *"MetS was defined according to the criteria proposed by the IDF [Zimmet et al., 2007].* A subject was classified as having MetS if he/she presented central obesity, defined by a large waist circumference, and at least two of the four criteria shown in Table 1." In the new version, Table 1 was excluded (on request) and the text containing the explanations has been added to the manuscript.

Reference:

Zimmet P, Alberti KG, Kaufman F, Tajima N, Silink M, Arslanian S, Wong G, Bennett P, Shaw P, Caprio S, IDF CONSENSUS GROUP (2007). The metabolic syndrome in children and adolescents–an IDF consensus report. Pediatr Diabetes 8:299-306.

23.2. In the second paragraph of the introduction (lines 62-69), make reference to bone development papers. Only one reference is used at the end of the paragraph.

Answer: You are absolutely right. In the new version of the manuscript a wide review of the introduction has been carried out and some paragraphs have been adjusted with inclusions and removals.

24.3. Line 66, the use of the word "intense" to describe the imbalance between bone formation vs resorption due to growth should be rephrased.

Answer: Thank you for the suggestion, the alterations have been made.

25.4. Line 67-68, “under normal physiological conditions” and “dependent phenomena”, is this in reference to growth in adolescents or a healthy adult? How bone remodeling is occurring during adolescence as a result of growth and maturation needs to be considered when describing this process.
Answer: Thank you for the suggestion, the alterations have been made.

26.5. Line 74 mentions a systematic review, please provide reference.
Answer: We have added the reference [3], thank you.

Reference:
Silva VND, Fiorelli LNM, Silva CC, Kurokawa CS, Goldberg TBL. Do Metabolic syndrome and its components have an impact on bone mineral density in adolescents? Nutrition Metabolism (Lond). 2017; 14:1-7.

27.6. Line 85, what is meant by “specialized literature”?
Answer: Thank you for the suggestion. We have rewritten the sentence to add clarity.

28.7. It is mentioned, line 80, that components of MetS are negatively correlated with BMD in adolescents. It may be worth including and referencing literature that has demonstrate negative impacts of excess weight in adolescents on BMD, BMC and/or bone strength, in addition to the incidence of increased fracture. There is some literature that has demonstrated negative correlations between BMI or excess weight on marks of bone turnover. This can and should be used to support some of the findings related to MetS and bone properties.
Answer: Dear reviewer, we appreciate your comment. Our group is dedicated to understanding bone markers during adolescence and their relationship with excess weight, which has resulted in some publications.

References:
Nóbrega da Silva V, Goldberg TBL, Mosca LN, Rizzo ACB, Teixeira AS, Corrente JE. Metabolic syndrome reduces bone mineral density in overweight adolescents. Bone. 2014; 66:1-7.
Mosca LN, Goldberg TBL, Silva VN, Kurokawa CS, Rizzo ACB, da Silva CC, Teixeira AS, Corrente JE. The impact of excess body fat on bone remodeling in adolescentes. Osteoporos Int. 2017. 28:1053–1062.

Silva VND, Fiorelli LNM, Silva CC, Kurokawa CS, Goldberg TBL. Do Metabolic syndrome and its components have an impact on bone mineral density in adolescents? Nutrition Metabolism (Lond). 2017; 14:1-7.

The impact of excess body fat on bone remodeling was evaluated in overweight, obese, and extremely obese adolescents. In adolescents with excess weight, it was observed that the higher the bone mineral content and bone mineral density values, the lower the levels of the biomarkers. Nutritional imbalances through excess had a negative effect on bone formation in this stage of life. In girls with excess weight, the biomarkers were higher in the 10 to 13-year age group and no significant differences were observed between groups according to nutritional status. In boys, the levels were higher in those aged 13 to 15 years. According to nutritional status, significant differences were only observed in mean S-CTx for the age groups of 10–15 years, with higher levels between overweight and obese adolescents aged 10–12 years and between obese and extremely obese adolescents aged 13–15 years. In girls, significant negative correlations were observed between lean mass, fat mass, and fat percentage and each of the three bone markers studied. There was no correlation between lean mass or fat mass and the three biomarkers in boys. The biomarker trends demonstrated across the age groups follow the age trends for growth velocity. *In conclusion*, the higher the fat percentage and fat mass in girls, the lower the levels of the biomarkers, indicating that excess body fat has a negative effect on the evolution of these markers during adolescence. (Mosca *et al*., 2017).

Our research group has been investigating explanations about biomarkers and bone remodeling in adolescents with and without MetS, especially considering the lack of data in the literature on this population of adolescents.

29.1. Please refer to general comment as it pertains to previously published paper that could be referenced in this section of the document.
Answer: Dear reviewer, we have added our previous studies, thank you.

References:

Nóbrega da Silva V, Goldberg TBL, Mosca LN, Rizzo ACB, Teixeira AS, Corrente JE. Metabolic syndrome reduces bone mineral density in overweight adolescents. Bone. 2014; 66:1-7.

Mosca LN, Goldberg TBL, Silva VN, Kurokawa CS, Rizzo ACB, da Silva CC, Teixeira AS, Corrente JE. The impact of excess body fat on bone remodeling in adolescentes. Osteoporos Int. 2017. 28:1053–1062.

Silva VND, Fiorelli LNM, Silva CC, Kurokawa CS, Goldberg TBL. Do Metabolic syndrome and its components have an impact on bone mineral density in adolescents? Nutrition Metabolism (Lond). 2017; 14:1-7.

30.2. Final paragraph of section 2.1 (lines 132-136) describe how participants were matched and compare to the 271 subjects. Consider moving this to the first paragraph of section 2.1 to make it clear earlier in the paper how participants were matched for the study analysis.

Answer: Dear reviewer, we appreciate your suggestion, which was fully accepted.

31.3. Why did the authors choose to match participants by chronological age, bone age, and pubertal stage? Please provide reference or justification for using these matching criteria and perhaps for not including anthropometric measures.

Answer: Dear reviewer, the choice to match individuals by these variables resulted from the great differences observed between pubescent children in different stages of growth and physical maturity (early, middle, and late puberty). In addition, regarding the definition of metabolic syndrome in adolescents according to International Diabetes Federation criteria, pubescents aged 10 to 16 **are analyzed as a single group**, with only the percentile of waist circumference varying and the cutoff points for the other biochemical markers being similar for all criteria. In addition, from a statistical point of view, non-matching could generate bias in the explanations about the MetS outcome itself if the sample was not minimally homogenized.

32.4. Are the authors aware and able to report how many of the female participants were pre vs. post-menarcheal, or had all female participants reached menarche?

Answer: Dear reviewer, each individual among these 271 adolescents was enrolled and followed up at our Adolescence Service. So we can assure you that, as explained in the text, they were included in the sample with care.

As more than 60% of the adolescents, both those in the MetS + and MetS- groups, were in late puberty, we can suggest that they had already menstruated (see Table 1 in the new version). However, we do not have these data to confirm our assumption, as it would be necessary to ask each individual included.

33.5. It is not clear how the information that was used for the inclusion criteria was collected. Was this done by interview, survey, or a specific questionnaire. Specifically, how was the nutritional information collected?

Answer: Dear Reviewer, I believe that the answer to the previous question will resolve any doubts. In the text we have written (line 93): "*The sample of adolescents of both sexes with and without MetS and with excess weight was selected based on the availability of care for new cases at the Adolescent Outpatient Clinic of the University Hospital, Botucatu Medical School (SP, Brazil) on the occasion of their first visit. Data were collected from March 2011 to March 2013".*

34.6. Line 141, potential editing error in sentence – "utilized to classify nutritional status".

Answer: Dear reviewer, we accept your suggestion and have edited the sentence.

35.7. Like 143 makes refence to growth curves. Are these based on WHO or CDC curves?

Answer: They are based on CDC growth charts.

Reference:

Kuczmarski RJ, Ogden CL, Guo SS, Grummer-475 Strawn LM, Flegal KM, Mei Z, Wei R, Curtin LR, Roche AF, Jhonson CL 2000. CDC Growth Charts for the United States: Methods and Development. Vital Health Stat. 2002; 11 246: 1-190.

Statistical Analysis/Results

36.1. Given that DXA was used, is it possible for the authors to provide data on lean and fat mass, or as a percentage, for the participants. Is there BMC data that can be reported?

Answer: Dear Reviewer, we have all these data in our parent spreadsheet. However, BMC data was also not presented in the study published in Bone (Nóbrega da Silva *et al*., 2014), as we have already commented to Reviewer 1. In that study, the analysis included all 271 adolescents, as shown in the table below.

Table 4. BMD and transformed BMD compared between adolescents of both genders with and without MetS.

**Parameter**	**Female (n=158)**			**Male (n=113)**		
**BMD**	**MetS (-) (n=137)**	**MetS (+) (n=21)**		**MetS (-) (n=95)**	**MetS (+) (n=18)**	
	Mean±SD		*p* value[a]	Mean±SD		*p*value[a]
BMD (g/cm²)						
BMD of lumbar spine	0.948±0.178	0.934±0.197	0.761	0.834±0.154	0.851±0.131	0.624
BMD of proximal femur	0.966±0.143	0.959±0.145	0.827	0.993±0.164	1.011±0.150	0.651
BMD of total body	0.973±0.102	0.951±0.104	0.387	0.956±0.108	0.941±0.089	0.543
BMD of subtotal body	0.869±0.089	0.862±0.094	0.744	0.874±0.106	0.867±0.095	0.782
BMD (g/cm²/ kg body weight)	**MetS (-)**	**MetS (+)**		**MetS (-)**	**MetS (+)**	
BMD of lumbar spine	0.013±0.002	0.012±0.001	0.003	0.011±0.001	0.010±0.001	0.001
BMD of proximal femur	0.014±0.001	0.012±0.002	0.006	0.013±0.002	0.012±0.002	0.015
BMD of total body	0.014±0.002	0.012±0.001	0.000	0.013±0.002	0.011±0.002	<0.00
BMD of subtotal body	0.012±0.001	0.011±0.001	0.001	0.012±0.001	0.011±0.002	0.033

[a]Student t-test for independent samples.
MetS (-): without metabolic syndrome; MetS (+): with metabolic syndrome.

ACCEPTED MANUSCRIPT

Regarding fat mass and percentage, we have already published the association with bone markers, identifying a negative association, indicating that the greater the fat mass or its percentage, the lower the results of the concentrations of the markers. In the conclusion of this paper we have written: *The higher the fat percentage and fat mass in girls, the lower the levels of the biomarkers, indicating that excess body fat has a negative effect on the evolution of these markers during adolescence.*

References:

Nóbrega da Silva V, Goldberg TBL, Mosca LN, Rizzo ACB, Teixeira AS, Corrente JE. Metabolic syndrome reduces bone mineral density in overweight adolescents. Bone. 2014; 66:1-7.

Mosca LN, Goldberg TBL, Silva VN, Kurokawa CS, Rizzo ACB, da Silva CC, Teixeira AS, Corrente JE. The impact of excess body fat on bone remodeling in adolescents. Osteoporos Int. 2017. 28:1053–1062.

37.2. Table 2, the n-values for the Tanner stages do not add up to be the n-values for the respective group.

Answer: You are right, in some teenagers we did not obtain Tanner stages, however, we obtained bone age from all of them and we know that there is a strong correlation between these events.

38.3. It is not clear what Tanner stages the authors are using to define participants as early, mid or late puberty. Please define this in the maturation section of the methods.

Answer: We used the classification proposed by Marshall & Tanner, however, for the number of adolescents in each group the following were adopted, Breast 1- 2 considered early puberty, Breast 3 mid puberty, and Breast 4 and 5 late puberty, as well as in genitals in male adolescents (Table 1 in this new version of the manuscript).

Reference:

Finkelstein JW. The endocrinology of the adolescent. Symposium of adolescent medicine. Pediatrics Clinic of North America.1980; 27:53-69.

39.4. Statistical analysis section states (Line 215-218) than an ANOVA was conducted to compare bone markers with the number of MetS components, however results are not presented in the document.

Answer: Dear reviewer, you are absolutely right, we have removed this analysis from our study and present it in the new version of the manuscript.

40.5. In terms of analysis, the age range (10-16yrs) in addition to the inclusion of all tanner stages into the analysis may be problematic and contributing to some of the correlations and findings.

Answer: Dear reviewer, we agree and also considered this a problematic situation when we outlined the study. However, the IDF uses this group of individuals to perform the classification of those with or without MetS, in addition to the matching that took into account bone and chronological age and the pubertal stages.

41. A) Bone marker concentrations change with maturity and there have been studies to show varying levels by tanner stage. I would expect to see negative correlations since BMD increases and bone marker concentrations decrease with age/maturation. It is not clear from the data and subsequent discussion why this relationship is significant in females and more so in those that have MetS. Is the number of MetS components, or a specific component, or perhaps their excess weight (percent body fat) a factor? Is it related to their maturation? Looking at the data more females are in late puberty compared to boys. Is the menarcheal status between the female MetS and non-MetS participants similar?

Answer: Dear Reviewer, all your questions are highly thought-provoking and we reflected on a number of them while writing our discussion. Our research group started on the topic by

analyzing bone density and mineral content and bone markers in eutrophic adolescents, male and female. Next, we analyzed the effects of obesity on bone density and bone markers in adolescents. Thus, we are aware of this negative relationship that the reviewer so adequately points out (references already presented). However, in order to avoid doubts that the age effect alone was responsible for the reduction in bone markers evidenced in the current study, we present in the discussion the following extract (line 263- 278):

"*Researchers have highlighted the importance of understanding the evolution of BMD and alterations in the levels of bone biomarkers during adolescence (Silva et al., 2012; Fortes et al., 2014). Fortes et al. (2014), evaluating BMD and its relationship with bone markers in a sample of 101 eutrophic female adolescents of 9 to 20 incomplete years, observed an expressive increase in BMD according to the progression of chronological and bone age. With respect to indicators of bone metabolism, higher mean biomarker levels (BAP=110.12 U/L, OC=26.49ng/mL, S-CTx=1.58 ng/mL) were found when the adolescents were in stage 3 of breast development and in early adolescence (11 to 12 years). The levels of bone biomarkers were lower at the end of adolescence (BAP=31.70 U/L, OC=4.98ng/mL, S-CTx=0.44 ng/mL). Similar results were recently published with the follow-up of 2,416 children and adolescents, from 3 months to 17 years of age, analyzing bone markers Osteocalcin, PINP, and CTX-I, 317 with obesity and the other eutrophic (Geserick et al., 2020).* ***During puberty, the authors demonstrated that the highest levels of bone markers occurred from 10 to 11 years (girls, Tanner 3) and 13 years (boys, Tanner 3–4). Results very close to those presented by Fortes et al. (2014) regarding age and pubertal stage.***

In addition, our current group of teenagers included were between 10-16 years old, with an average CA of around 12 years for girls and 13 years for boys and BA of around 13 years for both.

(Line: 279 – 291): *"In male adolescents, Silva et al. (2012) studied a sample of 87 eutrophic adolescents aged 10 to 18 years and concluded that serum biomarker levels differed significantly among age groups. The highest concentration of the biomarkers was observed between 13 and 15 years of age (BAP = 155.50U/L, OC = 41.63 ng/mL, S-CTx = 2.09 ng/mL) and the lowest concentration between 16 and 18 years (BAP= 79.80U/L, OC = 27.80 ng/mL, S-CTx = 1.60 ng/mL). Within this context, proportionally higher levels of bone biomarkers would be expected in the adolescents in this present study,* ***since the mean CA of girls with or without MetS was around 12 years of age and BA, around 13 years of age, while in boys both CA and BA were around 13 years of age. The concentrations obtained among adolescents without MetS in the present study were close to those observed among the eutrophic individuals analyzed in the studies cited*** *(Silva et al., 2012; Fortes et al., 2014). However, a*

significant reduction in both biomarkers of bone formation and resorption was observed in the group of adolescents with MetS compared to the matched group without MetS".

With respect to the negative correlations, this was part of one of the responses to the Editor (Comment 3):

References:

Silva CC, Kurokawa CS, Nga HS, Moretto MR, Dalmas JC, Goldberg TBL. Bone metabolism biomarkers, body weight, and bone age in healthy Brazilian male adolescents. J Pediatr Endocrinol Metab. 2012; 25: 479-484.

Fortes CMT, Goldberg TBL, Kurokawa CS, Silva CC, Moretto MR, Biason TP, Teixeira AS, Nunes HRC. Relação entre as idades cronológica e óssea e o estágio puberal das mamas com os biomarcadores ósseos e a densidade mineral óssea em adolescentes. J Pediatr (Rio J). 2014; 90:624-631.

Geserick M, Vogel M, Eckelt F, Schlingmann M, Hiemisch A, Baber R, Thiery J, Korner A, Kiess W, Kratzsch J. Children and adolescents with obesity have reduced serum bone turnover markers and 25-hydroxyvitamin D but increased parathyroid hormone concentrations - Results derived from new pediatric reference ranges. Bone. 2020;132:115-124.

42. B) Because of the changing bone marker concentrations with maturation it is not uncommon for studies to separate participants into groups based on tanner staging. Have the authors considered creating 2 tanner groups to help minimize maturation effects that come with having all tanners 1-5 grouped together?

Answer: Dear reviewer, we understand your concern and believe that our comments on the previous questions demonstrate why we did not perform the analysis based on pubertal stages. In addition, there is no recommendation for the classification of MetS by pubertal stages in adolescents, the 10-16 age group being considered as a single group (IDF, 2007).

Reference:

Zimmet P, Alberti KG, Kaufman F, Tajima N, Silink M, Arslanian S, Wong G, Bennett P, Shaw P, Caprio S, IDF CONSENSUS GROUP (2007). The metabolic syndrome in children and adolescents–an IDF consensus report. Pediatr Diabetes 8:299-306.

43.C) As well, given that BMD is affected by body size, have the authors considering or tried adjusting their analysis by controlling for various anthropometric measures (i.e. height)?

Answer: Dear reviewer, reviewer 1 asked a similar question. We ask you to please consider the answer to question 4.

Discussion

44.1. Line 256 – in reference to the "behavior"' of bone biomarkers. Please rephrase to be clearer as to what the results were.

Answer: Dear reviewer, we have made the adjustments in the new version of the manuscript.

45.2. Line 259 – states that BMD was negatively correlated with all bone markers in adolescents with MetS. However, this was only the case with females and not males. Please correct this statement.

Answer: Dear reviewer, you are right, we have added that the correlations were negative only in girls (First paragraph of the discussion).

46.3. Lines 261-268 – authors discuss collinearity of the bone markers. What is the purpose of this finding and how does it relate to the literature and data?

Answer: Dear reviewer, you are right, we have deleted this paragraph.

47. 4. Line 268 – what is BMT?

Answer: Dear reviewer, Bone markers of turnover (BMT). We are sorry we did not write this in full.

48. 5. Provide reference at end of sentence (line 271)

Answer: Dear reviewer, we have added the reference [30] to the end of the sentence in this new version of the manuscript.

Reference:

Weaver CM, Gordon CM, Janz KF, Kalkwarf HJ, Lappe JM, Lewis R, Okarma M, Wallace TC, Zemel BS. The national osteoporosis foundation's position statement on peak bone mass development and lifestyle factors: a systematic review and implementation recommendations. Osteoporos Int. 2016; 27:1281-1386.

49. 6. Paragraph (Line 277-295) – Authors discuss bone marker levels by age and tanner stage. Please be specific if you are referring to males vs. females in those studies. And which of these studies used participants with MetS. As well, how does these studies and their grouping compare given that the current study includes all tanner stages into one group. Please refer to comment 5b in previous section.

Answer: Dear reviewer, the answers to this comment can be found in other questions (41A and 42B). We emphasize that the studies cited were developed for eutrophic girls and boys, independently. We did not detect articles that evaluate bone turnover markers in adolescents with MetS in the literature. Some authors have analyzed Osteocalcin and its relationship with some MetS components, and these are mentioned in the discussion.

50.7. Line 307 – Unclear what the results of Tan et al were and how they compare to the current findings. Please elaborate.

Answer: Dear reviewer, this paragraph has been reviewed and adjusted, highlighting the negative correlation between OC and MetS that occurred for males. Tan et al. (2011) evaluated 2,344 **Chinese men** between 20 and 69 years of age and found a negative association between OC levels and the presence of MetS. These results agree with our findings, however, in our results the reductions in OC were significant and negative only in female adolescents with MetS.

Reference:

Tan A, Gao Y, Yang X, Zhang H, Qin X, Mo L, Peng T, Ning X, Mo Z. Low serum osteocalcin level is a potential marker for metabolic syndrome: results from a Chinese male population survey. Metabolism. 2011; 60:1186-1192.

51. 8. Line 318 – what do the authors mean by “biochemical alterations”?

Answer: We ask the Reviewer to please note the response to Comment 7 to Reviewer 1.

52.9. Paragraph (lines 324-327) – small paragraph talking about OC. Consider reorganizing and placing it within previous paragraph where authors discuss OC. As well, please provide clearer discussion of findings when comparing current study results with study using eutrophic children, when such a group was not used in the current study.

Answer: Dear Reviewer, we appreciate your suggestion and have deleted the paragraph, as the comparison was actually made with eutrophic children.

53.10. Lines 334-335 – please provide references

<u>Answer</u>: Dear Reviewer, we agree and have reorganized the entire paragraph including references.

54.11. It is not clear the connection the authors are trying to make between different types of OC and various other physiological markers, and how this relates to the finding of the current study.

<u>Answer</u>: Dear Reviewer, we agree and have reorganized the text of the discussion in this new version of the manuscript.

55.12. Please provide discussion on limitations for the current study. Please include some methodological limitations of using DXA measurement with children and adolescents.

<u>Answer</u>: Dear Reviewer, we have included the study limitations at the end of the discussion section. Details of the evaluation of bone mineral density are highlighted in the methods section.

56.13. Line 426 and 427 – the authors mention "raising new hypotheses" and suggesting possible "design for future studies", although hypotheses were not presented within the document. Can the authors please include some examples for potential future studies or considerations.

<u>Answer</u>: Dear Reviewer, we have included this information in the new version of the manuscript. Thank you again for the detail of the review and we hope to have met your expectations.
