# Supplementary material for: Impact of metabolic syndrome and its components on bone remodeling in adolescents
Source: PLoS One. 2021 Jul 1;16(7):e0253892. doi: 10.1371/journal.pone.0253892 (PMC8248706; doi:10.1371/journal.pone.0253892)
Supplement: Attachment — Submitted filename: Response to Reviewers.docx [file pone.0253892.s004.docx]

**PONE-D-20-24612R1**

**Impact of metabolic syndrome and its components on bone remodeling in adolescents
PLOS ONE**

**To Benjamin Udoka Nwosu, MD**

**Academic Editor**

**PLOS ONE**

**Dear Professor Udoka Nwosu**

First, we are grateful for the opportunity to send a revised version of our paper and for the excellent comments from the reviewer regarding our article “**Impact of metabolic syndrome and its components on bone remodeling in adolescents”**

**Editor Comments**

Please carefully review the comments below and address each one in a comprehensive manner.

Ensure that height, weight, and BMI are expressed as z-scores in your Table 1 and other appropriate areas.

Dear Prof Udoka Nwosu, we thank you for the suggestion and have included the z-score for height, weight, and BMI.

Please ensure that your BMD data are also reported as z-scores which is the standard method or reporting in children and adolescents. This should be easy to extract from the radiological reports.

Regarding the z-scores of BMDs, we would ask you to understand the reasons we are unable to meet your request. The data referring to Densitometries were obtained between 2011 and 2012 and all anthropometric, biochemical, and densitometric variables were transferred to a spreadsheet. At that time, only the BMDs were transcribed and not the z-scores. The data for the 271 adolescents were published in another journal (BONE) in 2014. At this point, we would like to point out that the Densitometry machine has been deactivated and our University has acquired another machine, of another brand. In addition, the hospital has undergone a digitization process, which took place in 2012. Thus, printed data are extremely difficult to locate after all this time. We would be grateful for any guidance on your part on how to determine the z-score, and compare with which benchmarks, as we do not have Brazilian benchmarks for adolescent densitometry, in addition to the studies cited in the study.

**Review Comments to the Author**

**Reviewer #1:** The authors made major revisions to the manuscript after the first round of reviews. The manuscript is clearer and presents major findings. I would suggest graphs for some of the findings vs. tables.

My comments are incorporated within the comment session and the manuscript in red and are attached to this review.

In brief:

**Abstract: line 48** - consider revision

Thank you for the suggestion, the alterations have been made.

**Methods:**

**lines 166-167:** could authors comment on anyone with impaired glucose tolerance and/or T2DM? This cohort present a more significant metabolic milieu and would be interesting to see the effects on bone and may need to be separated from non-DM. This should be clarified for both cohorts (MetS - and MetS+).

Dear Reviewer, we know how frequent Type 2 Diabetes in the USA is among obese, severely obese, and MetS adolescents, but in our studies, including an article published in 2013, we report that for both sexes, altered glycemia was the least prevalent MetS factor among all factors analyzed. In the general sample of 271 adolescents evaluated, 2% of male adolescents and 3% of females presented glucose intolerance.

When we analyzed this factor in the group of 84 paired adolescents, in the MetS+ group, 3 of 24 female adolescents had glucose intolerance and none among the MetS-. For boys with MetS+, 2 out of 18 had glucose intolerance and none among the MetS-.

Given this information, we do not have enough numbers to analyze your important suggestion.

***Reference***

Rizzo ACB, Goldberg TBL, Silva CC, Kurokawa CS, Nunes HRC, Corrente JE.

[Metabolic syndrome risk factors in overweight, obese, and extremely obese Brazilian adolescents](javascript:void(0)). Nutrition journal 12 (1), 1-7. <https://doi.org/10.1186/1475-2891-12-19>

**session 2.5 of Methods:** BMD is not height adjusted, though groups didn't differ in height. I would include and explain this in the methods.

Results: Also, I would suggest to the authors to include and do analysis of BMD z-scores as BMD done in pediatric population. This is a major point.

Dear Reviewer,

We have answered Prof. Nwosu on this important consideration and the problems involved with the BMD z score.

*“Regarding the z-scores of BMDs, we would ask you to understand the reasons we are unable to meet your request. The data referring to Densitometries were obtained between 2011 and 2012 and all anthropometric, biochemical, and densitometric variables were transferred to a spreadsheet. At that time, only the BMDs were transcribed and not the z-scores. The data for the 271 adolescents were published in another journal (BONE) in 2014. At this point, we would like to point out that the Densitometry machine has been deactivated and our University has acquired another machine, of another brand. In addition, the hospital has undergone a digitization process, which took place in 2012. Thus, printed data are extremely difficult to locate after all this time. We would be grateful for any guidance on your part on how to determine the z-score, and compare with which benchmarks, as we do not have Brazilian benchmarks for adolescent densitometry, in addition to the studies cited in the study.”*

**Table 1** and results section discussing Tanner staging: The authors included Tanner stage 1 as early puberty. Tanner 1 is pre-pubertal stage. I suggest authors to correct this and separate subjects with Tanner stage 1 if there were any.

Thank you for your important suggestion. The problem has been solved.

**Discussion:**

Much of the beginning of the discussion is spent on age and puberty affects on bone mass accrual. However, the study's focus is a cross-sectional assessment of MetS on BMD and bone remodeling of age matched groups. Though the groups are heterogeneous incorporating wide age range (10-16 years) and all pubertal stages, there were no clear analysis of affects of age and puberty on the aims of the study in the current study. The first part of the discussion should be revised and shortened to tie in with the findings and the focus of the study.

We appreciate your recommendations and have made the adjustments.

Also, discussion is focused mainly around bone remodeling markers. There is no discussion of the other findings such as BMD, opposite findings in correlation analyses (Table 3-5), which need to be explained and elaborated on. I suggest major revisions to the discussion session.

**Line 256:** states at the beginning of puberty, which is not entirely correct. In girls, growth velocity peak is late Tanner 2 to 3 of breast development. In boys, growth spurt is mainly TS 3, occasionally to TS 4, which is mid to later puberty. Similarly, bone mass accrual happens later in boys than girls. The authors also define TS 3 as mid puberty, thus, the statement in line 256 should be corrected.

We thank you for your comments and hope we have met your requests.

Line 256: states at the beginning of puberty, which is not entirely correct. In girls, growth velocity peak is late Tanner 2 to 3 of breast development. In boys, growth spurt is mainly TS 3, occasionally to TS 4, which is mid to later puberty. Similarly, bone mass accrual happens later in boys than girls. The authors also define TS 3 as mid puberty, thus, the statement in line 256 should be corrected.

We thank you for your comments and have made the necessary changes.

Line 260-261: Authors refer to international recommendations, but no recommendations are provided or sited. It is not clear what authors are referring to.

Paragraph lines 292-294: is out of place as the discussion in preceding and subsequent paragraphs is about biochemical bone remodeling markers. Though, BMD should be discussed as in earlier suggestion. Please see other comments and questions within the discussion session of the manuscript: highlighted and annotated in red.

Thank you for the generous and detailed review.
