# Supplementary material for: Impact of metabolic syndrome and its components on bone remodeling in adolescents
Source: PLoS One. 2021 Jul 1;16(7):e0253892. doi: 10.1371/journal.pone.0253892 (PMC8248706; doi:10.1371/journal.pone.0253892)
Supplement: Attachment — Submitted filename: Response to Reviewers.docx [file pone.0253892.s005.docx]

To Benjamin Udoka Nwosu, MD

Academic Editor

PLOS ONE

Dear Prof Udoka Nwoso,

We thank you for your important suggestions that helped improve our manuscript.

Below please find our replies to the suggestions of the reviewers. We again thank the reviewers for their important comments and the detailed appraisal of our manuscript.

We hope to have clarified all doubts and remain at your disposal for further clarifications.

Sincerely,

Prof. Tamara Goldberg and authors

**Reviewers' comments:**

Reviewer #1: Author’s response to DXA z-score question:

**Answer**:

Dear Reviewer,

We have answered Prof. Nwosu on this important consideration and the problems involved with the BMD z score.

“Regarding the z-scores of BMDs, we would ask you to understand the reasons we are unable to meet your request. The data referring to Densitometries were obtained between 2011 and 2012 and all anthropometric, biochemical, and densitometric variables were transferred to a spreadsheet. At that time, only the BMDs were transcribed and not the z-scores. The data for the 271 adolescents were published in another journal (BONE) in 2014. At this point, we would like to point out that the Densitometry machine has been deactivated and our University has acquired another machine, of another brand. In addition, the hospital has undergone a digitization process, which took place in 2012. Thus, printed data are extremely difficult to locate after all this time. We would be grateful for any guidance on your part on how to determine the z-score, and compare with which benchmarks, as we do not have Brazilian benchmarks for adolescent densitometry, in addition to the studies cited in the study.”

***Reviewer’s suggestions:***

Lines 180 to 195: DXA methods: Please include a relevant line with the explanation of BMD DXA data and lack of Z-scores in methods as per authors explanation (see above).

Also, can consider extrapolating Z-scores from on-line calculator from CHOP (Zemel, et al. (2011). Revised Reference Curves for Bone Mineral Content and Areal Bone Mineral Density According to Age and Sex for Black and Non-Black Children: Results of the Bone Mineral Density in Childhood Study. J Clin Endocrinol Metab, 96(10), 3160–3169. <https://zscore.research.chop.edu/bmdCalculator.php> ). if model of the machine used is the same. If deemed appropriate to include by the authors and editors, this would need to be clearly explained in the methods

**Answer**: Dear Reviewer, as we explained earlier, we could not redeem the BMD for age Z-score as our densitometry machine has been deactivated. Thank you for sending the references and for the availability of the calculator. Thus, we performed the BMD for age Z-score and, even by the equation proposed by Zemel et al. (2011), contained in the same calculator, from the study “Revised Reference Curves for Bone Mineral Content and Areal Bone Mineral Density According to Age and Sex for Black and Non-Black Children: Results of the Bone Mineral Density in Childhood Study. J Clin Endocrinol Metab, 96(10), 3160–3169, a BMD for height- for- age Z-score (HAZ).

As for the BMD for age Z-score, the results have been added to Table 1. As noted, they do not differ significantly according to sex between those with MetS– and MetS+. In addition, because the height between the matched groups does not differ statistically, as well as the height Z-score (Table 1), we performed the BMD calculations for height- for- age Z-score (HAZ) and, with the exception of the Lumbar BMD for height-for-age Z-score (HAZ), among boys, no other results differed significantly.

Just for your information, we have placed the results in the table below:

Table: Values of BMD for height- for- age Z-score (HAZ)

| **Values of BMD adjusted BMD Z-scores based on HAZ.** | | | | | | | |
| --- | --- | --- | --- | --- | --- | --- | --- |
|  | **Female (n=48)** | | | **Male (n=36)** | | | |
|  | **MetS (-) (n=24)** | **MetS (+) (n=24)** |  | **MetS (-)**  **(n=18)** | **MetS (+)**  **(n=18)** |  |  |
|  | **Mean ±** SD | | **P value** | **Mean ± SD** | | **P value** |  |
| Lumbar BMD | 0.558 ± 1.00 | 0.547 ± 1.22 | 0.97 | -0.511 ± 1.05 | 0.620 ± 0.88 | 0.049* |  |
| Total body BMD | -0.665 ± 1.34 | -0.377 ± 1.10 | 0.43 | -0.740 ± 0.83 | -0.628 ± 0.84 | 0.690 |  |
| Subtotal BMD | -0.684 ± 1.47 | -0.223 ± 0.90 | 0.20 | -0.601 ± 0.75 | -0.474 ± 0.83 | 0.630 |  |
| Lumbar Spine BMD for Age Z-Score adjusted for HAZ (height-for-age Z-score)  Total Body BMD for Age Z-Score adjusted for HAZ (height-for-age Z-score)  Total Body Less Head BMD for Age Z-Score adjusted for HAZ (height-for-age Z-score) | | | | | | | |

And in the study, we detail:

*As our densitometry machine has been deactivated, it was impossible to retrieve the results of the BMD for age Z-score, in view of the long time since the evaluations were performed. Thus, we calculated the Lumbar BMD for age Z-score, Total body BMD, and Subtotal body BMD for age Z-score using the calculator https://zscore.research.chop.edu/bmdCalculator.php [28], to offer a guideline for readers*. *The effect of body size on bone mass can lead to misinterpretations when comparing individuals of different heights and body compositions. In the current study, the statures and height for age Z-scores did not differ between MetS + and MetS- groups for females (p=0.167; p=0.833) and males (p=0.728; p=0.881) respectively, and we sought to minimize body size-induced biases in bone mass estimates by using BMD transformed by body weight (g/cm²/kg body weight) [29]. This transformation can present limitations. Thus, authors use an estimate of volumetric bone mineral apparent density, BMAD (grams per cm^3^), published by Bachrach et al. [30] and adjusted by Zemel et al [31]. Zemel et al. [31] use a complex predictive equation of height-for-age Z-score (HAZ) (results not shown). All assessments were performed by only one technician trained for this purpose who had no knowledge of the research. In addition, the instructions of the manufacturer of the device and the standards recommended by the International Society for Clinical Densitometry [32] were followed.”*

28. Zemel BS, Kalkwarf HJ, Gilsanz V, Lappe JM, Oberfield S, Shepherd JA, Frederick MM, Huang X, Lu M, Mahboubi S, Hangartner T, Winer KK. Revised reference curves for bone mineral content and areal bone mineral density according to age and sex for black and non-black children: results of the bone mineral density in childhood study. J Clin Endocrinol Metab. 2011 Oct;96(10):3160-9.
30. Bachrach LK, Hastie T, Wang MC, Narasimhan B, Marcus R. Bone mineral acquisition in healthy Asian, Hispanic, black, and Caucasian youth: a longitudinal study. J Clin Endocrinol Metab.1999; 84:4702–4712.
31. Zemel BS, Leonard MB, Kelly A, Lappe JM, Gilsanz V, Oberfield S, Mahboubi S, Shepherd JA, Hangartner TN, Frederick MM, Winer KK, Kalkwarf HJ. Height adjustment in assessing dual energy x-ray absorptiometry measurements of bone mass and density in children. J Clin Endocrinol Metab. 2010; 95(3):1265-1273.

Lines 221 to 227: Table 1 results: I suggest including the results of BMD from table 1 here as they are part of the aims.

**Answer:** We appreciate your suggestion and have made the adjustments.

We have added in the manuscript: “*The comparison of lumbar spine, proximal femur, and total and subtotal body BMD values according to sex and the presence of MetS revealed no significant differences. However, when BMD was transformed to BMD per kilogram of body weight, the female adolescents with MetS exhibited significant decreases in BMD at all sites evaluated (p<0,01) and the males for total and subtotal body BMD (p<0.05) (Table 1)*”.

**Discussion:**

Lines 253-259: It is not clear why BMD results in groups with and without MetS are not being discussed. Authors mention that they have previously reported BMD results. Assuming this study includes new results comparing the groups with and without MetS to evaluate the effects of MetS on BMD (Table 1), then they should be discussed in addition to MetS effects on bone remodeling that authors discuss well and in depths.

Authors aims:

“In view of the results already published [3,5,6], the objective of the current study was to evaluate the impact of MetS on BMD and of MetS and each of its components and on biochemical markers of bone formation and resorption in adolescents.”

**Answer:** We are grateful for the suggestion and have adjusted the objectives and also added the information requested in the first paragraph of the discussion, between lines 253-259.

*In view of the results already published [3,5,6], the objective of the current study was to evaluate the impact of MetS on BMD, as well as the impact of MetS and each of its components on biochemical markers of bone formation and resorption in adolescents.*

add to first paragraph of the discussion…..

*Our results showed that adolescents with excess weight and MetS exhibited reduced transformed BMD values at all sites for females, and total and subtotal body densities for males when compared to adolescents without MetS who were matched for chronological age, bone age, and pubertal stage for each sex.*

With regard to the 3rd paragraph of the Discussion, we have added...

“We have previously reported that adolescents with excessive weight and MetS exhibited reduced transformed BMD values at all sites (Lumbar spine (L1-L4), proximal left femur, and total and subtotal) compared to adolescents without MetS [5] and the results of the current study corroborate those previously published. In a previous study [5] female adolescents with a large waist circumference…”

Line 271: reference of previously published data needed.

**Answer:** We thank you and have added the reference [5] in this line as well.

Line 278: newly added discussion on CMR z-score. Please define/describe what it means.

**Answer:** We thank you and have added the meaning of CMR z-score.

"Confirming our evidence, a recent study evaluated 306 female adolescents aged 9 to 12 years, analyzing the isolated and combined effect of cardiometabolic risk factors (CMR) (calculated by the CMR z-score of the included sample where CMR z-scores were created from the logarithmic transformed form of the variables: homeostatic model assessment of insulin resistance (HOMA-IR), C-reactive protein (CPR), triglycerides (TG), high-density lipoprotein cholesterol (HDL-c), low-density lipoprotein cholesterol (LDL-c), and mean arterial blood pressure (MAP)) on bone mass using DXA together with the evaluation of bone geometry, vBMD, and bone strength through the use of peripheral quantitative computed tomography (pQCT) in the tibia and femur.”.

**Reviewer #3:** This is a well written manuscript. The data on bone biomarkers is interesting.

Minor comments:

1. Lines 269- 271: Need reference for the transformed BMD values.

**Answer:** We appreciate your suggestion and have included the reference.

2. Table 1: Transformed BMD values are presented. Please describe the methodology for transforming BMD under statistical methodology.

**Answer:** We appreciate the suggestion and have placed the information where recommended (note: Table 1).

3. Lines 42, 43: Reword as “The adolescents with excess weight and MetS exhibited a significantly lower transformed BMD and reduced concentrations of BAP, …

**Answer**: Thank you very much for your suggestion.

4. Ideally bone mineral content (BMC) (grams) and areal BMD (BMC/bone area g/cm2) are to be adjusted as whole body bone mineral apparent density (BMAD, g/cm3), calculated using the formula, BMC/[whole body mineral area2/body height]. The authors state that both groups had similar heights. It is important to mention the limitations of not reporting the bone mineral apparent density(BMAD) -a size-adjusted measure of DXA BMD.

**Answer:** We appreciate your suggestion and have included this as a limitation.

Although the transformation used is available in the literature [29], it may have limitations with regard to the analysis carried out because, ideally, some authors use an estimate of volumetric bone mineral apparent density, BMAD (grams per cm^3^), disclosed by Bachrach et al. [30] and adjusted by Zemel et al. [31]. In Bachrach's study the expression whole body BMC/height (cm) was calculated to adjust for whole body bone size, with statistical models according to sex and ethnicity. The study by Zemel et al. [31] used a complex predictive equation of height-for-age Z-score (HAZ). Due to the fact that our groups are paired, as previously indicated, with stature and height- for- age Z-score that did not differ statistically, the results are presented only in the Letter.

| **Values of BMD adjusted BMD Z-scores based on HAZ.** | | | | | | | |
| --- | --- | --- | --- | --- | --- | --- | --- |
|  | **Female (n=48)** | | | **Male (n=36)** | | | |
|  | **MetS (-) (n=24)** | **MetS (+) (n=24)** |  | **MetS (-)**  **(n=18)** | **MetS (+)**  **(n=18)** |  |  |
|  | **Mean ±** SD | | **P value** | **Mean ± SD** | | **P value** |  |
| Lumbar BMD | 0.558 ± 1.00 | 0.547 ± 1.22 | 0.97 | -0.511 ± 1.05 | 0.620 ± 0.88 | 0.049* |  |
| Total body BMD | -0.665 ± 1.34 | -0.377 ± 1.10 | 0.43 | -0.740 ± 0.83 | -0.628 ± 0.84 | 0.69 |  |
| Subtotal BMD | -0.684 ± 1.47 | -0.223 ± 0.90 | 0.20 | -0.601 ± 0.75 | -0.474 ± 0.83 | 0.63 |  |
| Lumbar Spine (BMD) for Age Z-Score adjusted for HAZ (height-for-age Z-score)  Total Body BMD for Age Z-Score adjusted for HAZ (height-for-age Z-score)  Total Body Less Head BMD for Age Z-Score adjusted for HAZ (height-for-age Z-score) | | | | | | | |

May add these references:

We thank you for sending the references, they have all been added to the study.

Bachrach LK, Hastie T, Wang MC, Narasimhan B, Marcus R1999Bone mineral acquisition in healthy Asian, Hispanic, black, and Caucasian youth: a longitudinal study. J Clin Endocrinol Metab84:4702–4712.

Height Adjustment in Assessing Dual Energy X-Ray Absorptiometry Measurements of Bone Mass and Density in Children; Babette S. Zemel, Mary B. Leonard, Andrea Kelly, Joan M. Lappe,Vicente Gilsanz, Sharon Oberfield, Soroosh Mahboubi, John A. Shepherd,Thomas N. Hangartner, Margaret M. Frederick, Karen K. Winer,and Heidi J. Kalkwarf.
